# A novel taxane SB-T-101141 triggers a noncanonical ferroptosis to overcome Paclitaxel resistance of breast cancer via iron homeostasis-related KHSRP

**DOI:** 10.1038/s41419-025-07710-0

**Published:** 2025-05-19

**Authors:** Xiaomei Zhang, Ying Fang, Dade Rong, Jie Li, Zhe Li, Huidan Qiu, Qiuxia Chen, Jing Yang, Changwei Wang, Junxiu Huang, Qin Zhao, Shulan Yang, Haihe Wang

**Affiliations:** 1 Centre for Translational Medicine, The First Affiliated Hospital, Sun Yat-sen University, Guangzhou, Guangdong China; 2 Institute of Precision Medicine, The First Affiliated Hospital, Sun Yat-sen University, Guangzhou, Guangdong China; 3 Department of Biochemistry, Zhongshan School of Medicine, Sun Yat-sen University, Guangzhou, Guangdong China; 4 Faculty of Health Sciences, University of Macau, Taipa, Macau China; 5 Engineering Research Center of Tibetan Medicine Detection Technology, School of Medicine, Xizang Minzu University, Xianyang, China; 6 Guangzhou Institutes of Biomedicine and Health, Chinese Academy of Sciences, Guangzhou, Guangdong China; 7 Clinical Medical Research Centre for Plateau Gastroenterological Diseases of Xizang Autonomous Region, Xizang Minzu University, Xianyang, China

**Keywords:** Cell death, Target identification, Pharmacology, Preclinical research

## Abstract

Acquired multidrug resistance impedes the clinical application of paclitaxel. Here, we disclosed that the taxane SB-T-101141 efficiently contributed to a novel ferroptosis-like cell death of Paclitaxel-resistant and parental breast cancer cells. Functionally, SB-T-101141 facilitated the production of iron and ferrous ions along with reactive oxygen species (ROS), composed of lipid ROS and lipid peroxidation-derived aldehydes, including malonaldehyde (MDA), and glutathione (GSH) depletion. Iron chelators and ROS scavengers significantly attenuated cell death, and the inorganic ROS rendered by SB-T-101141. However, the ferroptosis-associated lipid oxide inhibitors could not block the lipid ROS and cell death triggered by SB-T-101141. Meanwhile, via genome-scale CRISPR-Cas9 screening, we uncovered that SB-T-101141 bound to the KH-type splicing regulatory protein (KHSRP) to inhibit the iron-dependent expression of CDGSH iron sulfur domain 1 (CISD1) associated with iron homeostasis, which consequently led to a novel type of ferroptosis of breast tumors. Moreover, RNA deep sequencing indicated that SB-T-101141 synergistically enhanced the iron-dependent activation of JNK and PERK pathways via KHSRP. Altogether, our results here demonstrate the potential clinical application of SB-T-101141 as a novel ferroptosis inducer in Paclitaxel-resistant breast cancer treatment.

## Introduction

Breast cancer is the most prevalent malignancy among women [1]. During breast cancer treatment, patients suffer a high rate of drug toxicity, drug resistance, and recurrence [2]. Breast cancer has a distinct tumor heterogeneity, with multiple subtypes and differences in incidence, treatment options and prognosis for each subtype such as distinct molecular subtypes based on the expression of estrogen receptor (ER), progesterone receptor (PR), and human epidermal growth factor receptor type 2 (HER2) with the use of immunohistochemistry: luminal A, luminal B, HER2 and triple-negative breast cancer (TNBC) [3, 4]. Thus, a full understanding of the biological heterogeneity of breast cancer will lead to the development of more effective therapy concepts in personalized medicine. Paclitaxel is frequently used as the first-line chemotherapeutic drug for breast cancer as well as clinically applied for treating solid tumors such as ovarian cancer, hormone-refractory prostate cancer, and non-small cell lung cancer, which usually lack target-based chemicals [5]. Paclitaxel functions on the stabilization of microtubules and initiates a cascade of signaling pathways resulting in programmed cell death [6, 7]. However, the emergence of drug resistance from multidrug resistance (MDR) and adaptive mutations leads to attenuation or loss of Paclitaxel therapeutic efficacy. To overcome such drug resistance, the semi-synthesis of Paclitaxel derivatives has evolved, among them, Cabazitaxel is superior to Paclitaxel and Docetaxel, and can effectively treat Docetaxel-resistant tumors due to its low affinity to P-gp [8, 9]. Therefore, it is of great interest to produce effective alternatives to Paclitaxel as second-line substitutes to treat various cancers.

Ferroptosis is characterized as an iron-dependent form of programmed cell death, which is distinguished from apoptosis, necrosis, and autophagy in either morphology, biochemistry, or mechanism [10, 11]. Morphologically, ferroptotic cells show significant cell membrane leakage and alteration of mitochondrial morphology, but in the absence of nuclear changes and chromatin condensation [10]. Accumulating evidence indicates that induction of tumor cell ferroptosis will be a new promising strategy for cancer chemotherapy, such as breast cancer treatment [12, 13]. Further study also suggests that breast cancer cells are iron-dependent and more sensitive to ferroptosis inducers [14, 15]. In addition, dead cells resulting from ferroptosis can recruit immune cell infiltration to promote further immunosuppression of tumor cells [16]. Therefore, induction of appropriate ferroptosis is an alternative way to overcome tumor resistance to common therapies that usually induce tumor cell apoptosis. The perquisites of ferroptosis induction are accompanied by three main features, including an imbalance of iron metabolism, massive production of disruptive membrane lipid peroxides, and collapse of the GPX4-GSH system [17]. In addition, multiple factors that modulate iron metabolism and homeostasis are involved in ferroptosis [18]. However, the Paclitaxel derivatives known to induce ferroptosis based on iron homeostasis regulation are rare.

In this study, we demonstrated that the novel taxane SB-T-101141 strongly repressed the growth of breast tumors, especially in inhibiting Paclitaxel-resistant breast tumors and breast cancer organoids. SB-T-101141 resulted in the cell ultrastructural features like ferroptosis and elevated the levels of iron and ferrous ions, ROS, including lipid ROS, lipid peroxidation-derived aldehydes such as MDA, and GSH depletion. Nevertheless, lipid ROS induced by SB-T-101141 was not blocked by ferroptosis inhibitors. Simultaneously, we exhibited that SB-T-101141 triggered a novel ferroptosis to repress breast tumor growth by stably binding to KHSRP to inhibit the expression of CISD1 related to iron homeostasis. Furthermore, SB-T-101141 motivated iron-dependent activation of JNK and PERK pathways by KHSRP, which could be abolished by the inhibitors of JNK and PERK pathways. These together highlight the potential strategy of utilizing SB-T-101141 as a novel ferroptosis inducer for Paclitaxel-resistant cancer therapy.

## Results

### SB-T-101141 effectively inhibits breast tumor growth

**Fig. 1:**
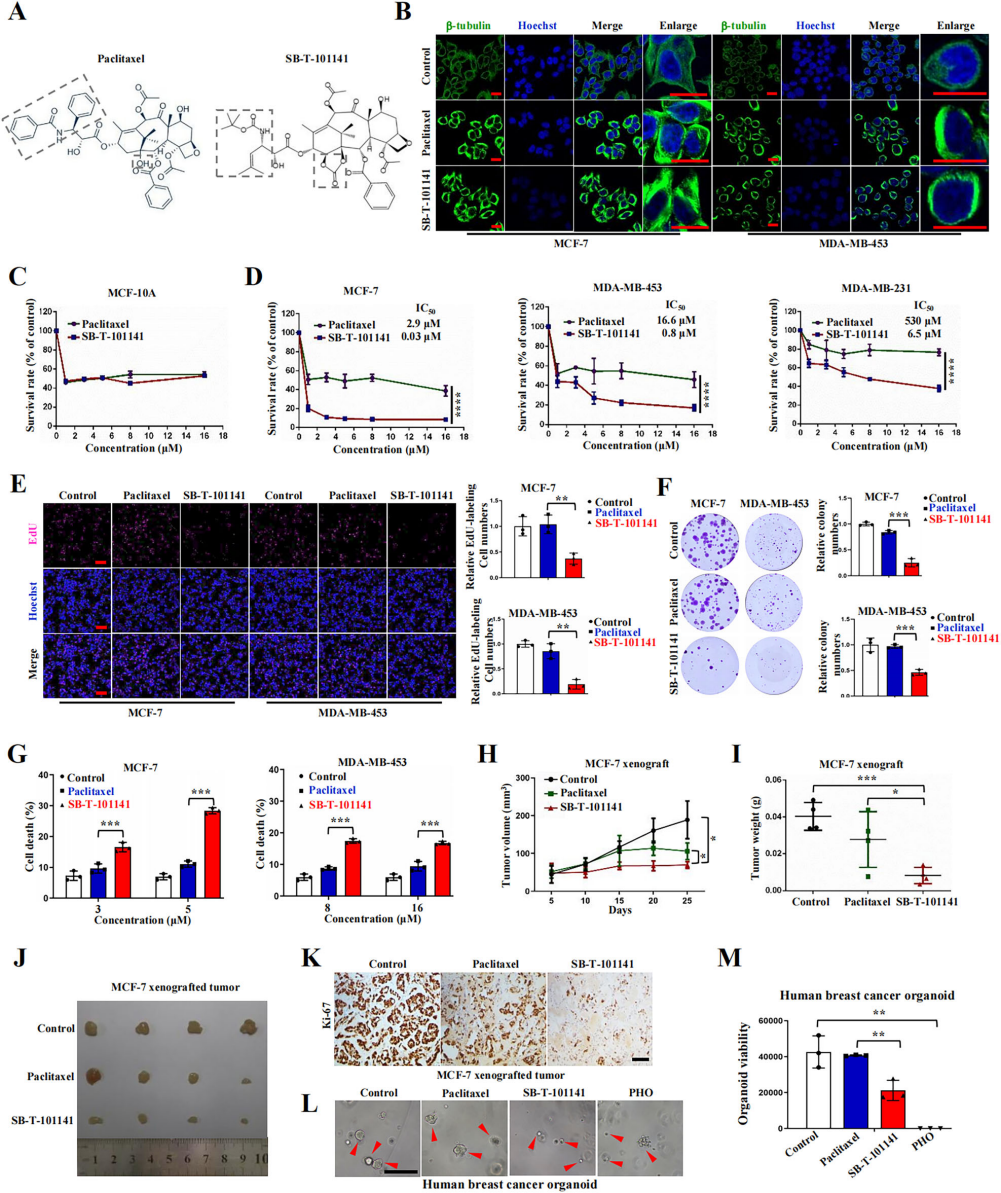
SB-T-101141 markedly inhibits breast tumor growth. **A** The chemical structures of Paclitaxel and SB-T-101141. **B** Immunofluorescence of MCF-7 and MDA-MB-453 cells treated with Paclitaxel (1 μM) and SB-T-101141 (1 μM) for 12 h, respectively. Bar indicates 20 μm. **C**, **D** Cell viability analyses of MCF-10A (**C**), MCF-7, MDA-MB-453 and MDA-MB-231 cells (**D**) at the indicated concentrations of Paclitaxel and SB-T-101141 for 72 h, respectively. A Two-way *ANOVA* test (mean ± SD, n = 3) is used. *P < 0.05, **P < 0.01, ***P < 0.001, and ****P < 0.0001. **E** EdU labeling of MCF-7 and MDA-MB-453 cells. Cells were treated with Paclitaxel (MCF-7, 2 μM; MDA-MB-453, 5 μM) and SB-T-101141 (MCF-7, 2 μM; MDA-MB-453, 5 μM) for 24 h and then labeled with EdU (left panel, bar indicates 100 μm), and relative EdU-labeled cell numbers were normalized and plotted (right panel). The student *t* test is used for statistical analysis (mean ± SD, n = 3). *P < 0.05, **P < 0.01, ***P < 0.001, ****P < 0.0001. **F** Colony formation of MCF-7 and MDA-MB-453 cells upon Paclitaxel (1 nM) and SB-T-101141 (1 nM) treatments, respectively. The colony was visualized with a crystal violet staining (left panel). The statistical results (right panel) were analyzed with the student *t* test (mean ± SD, n = 3). *P < 0.05, **P < 0.01, ***P < 0.001, ****P < 0.0001. **G** PI staining of MCF-7 and MDA-MB-453 cells treated with Paclitaxel and SB-T-101141 for 24 h, respectively. PI-labeled cells were quantified and analyzed with the student *t* test (mean ± SD, n = 3). *P < 0.05, **P < 0.01, ***P < 0.001, and ****P < 0.0001. **H**–**K** Xenografted tumor formation of MCF-7 cells. The mice were treated with Paclitaxel or SB-T-101141. The tumor size (**H**) was monitored (mean ± SD, n = 4; *ANOVA* test), and dissected for weighting (**I**–**J**) (mean ± SD, n = 4; student *t* test). Tumors were subjected to IHC assay (**K**) with the indicated antibody. The bar indicates 100 μm. *P < 0.05, **P < 0.01, ***P < 0.001, and ****P < 0.0001. **L**, **M** Breast cancer organoids viability analysis. Freshly established breast cancer organoids (1000 organoids/well/96 plate) were treated with Paclitaxel (9 μM), SB-T-101141 (9 μM) or positive control phenylarsine oxide (PHO) (1 μM) for 120 h, the organoids were then visualized under a microscope (**L**), the viability of organoids was detected and analyzed (**M**) using a student *t* test (mean ± SD, n = 3) (*P < 0.05, **P < 0.01, ***P < 0.001, and ****P < 0.0001). Bar indicates 50 μm.

The novel 3^rd^-generation taxane, SB-T-101141 derived from DAB and designed from the Ojima laboratory, was synthesized as previously [19] (Fig. 1A). Paclitaxel stabilizes microtubules by promoting the assembly of alpha and beta tubulin subunits [20, 21]. In line with Paclitaxel, SB-T-101141 also efficiently induced microtubule polymerization, compared to the untreated cells with evenly distributed microtubules in the cytoplasm (Fig. 1B). Meanwhile, both Paclitaxel and SB-T-101141 enhanced tubulin expression (Fig. S1A). However, SB-T-101141 presented similar cytotoxicity to Paclitaxel in non-cancerous human mammary MCF-10A cells (Fig. 1C), but exhibited more significant cytotoxicity than Paclitaxel in different breast cancer cells with lower IC_50_ (Fig. 1D). Moreover, SB-T-101141 exerted more strong inhibitory effects than Paclitaxel with decreased cell proliferation, colony formation, and increased cell death in various cancer cells (Figs. 1E–G and S1B, C). In addition, SB-T-101141 could strongly repress tumor growth in xenografted tumor mice inoculated with human MCF-7 breast cancer cells (Fig. 1H–K) and MDA-MB-453 cells (Fig. S1E–G), without markedly affecting mouse body weight (Fig. S1D, S1H), compared with Paclitaxel, respectively. Moreover, SB-T-101141 effectively inhibited the growth of patient breast cancer organoids (Fig. 1L, M). These findings together provide a therapeutic strategy to utilize SB-T-101141 for targeting breast tumors.

### SB-T-101141 triggers ferroptosis via iron-dependent ROS accumulation

**Fig. 2:**
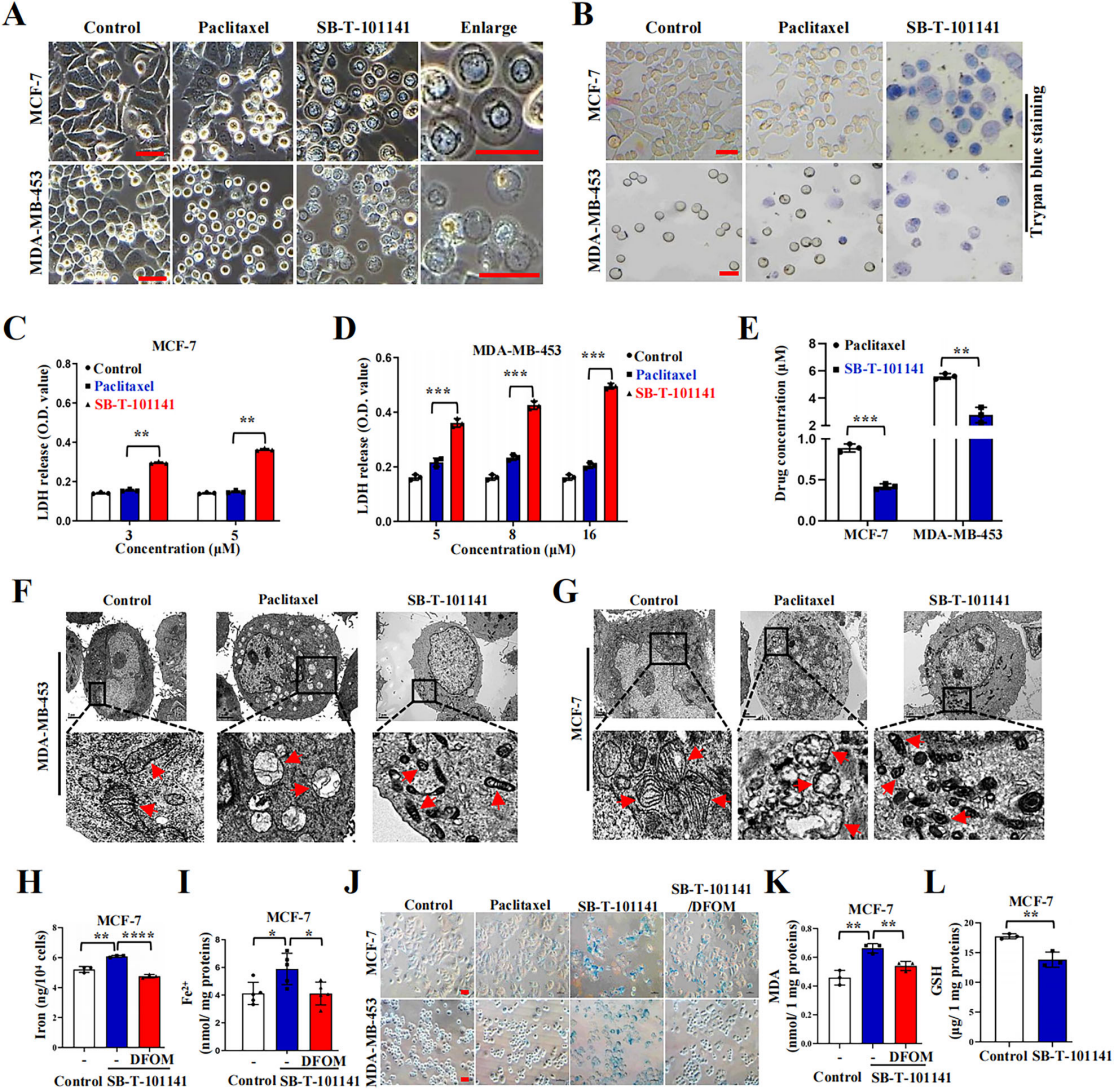
SB-T-101141 induces an iron-dependent cell death. **A** Cell morphology of MCF-7 and MDA-MB-453 cells treated with Paclitaxel (MCF-7, 5 μM; MDA-MB-453, 16 μM) and SB-T-101141 (MCF-7, 5 μM; MDA-MB-453, 16 μM) for 72 h, respectively and visualized under a microscope. Bar indicates 100 μm. **B** Trypan blue staining of MCF-7 and MDA-MB-453 cells treated with Paclitaxel (MCF-7, 5 μM; MDA-MB-453, 16 μM) and SB-T-101141 (MCF-7, 5 μM; MDA-MB-453, 16 μM) for 48 h and visualized under a microscope. Bar indicates 100 μm. **C**, **D** LDH release analysis of MCF-7 (**C**) and MDA-MB-453 (**D**) cells treated with Paclitaxel or SB-T-101141 for 48 h. The cell supernatant was collected and detected. The results were analyzed with the student *t* test (mean ± SD, n = 3). *P < 0.05, **P < 0.01, ***P < 0.001, and ****P < 0.0001. **E** Mass spectrometry of the intracellular amount of Paclitaxel and SB-T-101141 in MCF-7 and MDA-MB 453 cells. Cells were treated with Paclitaxel (MCF-7, 3 μM; MDA-MB-453, 8 μM) and SB-T-101141 (MCF-7, 3 μM; MDA-MB-453, 8 μM) for 4 h. The results were analyzed using the student *t* test (mean ± SD, n = 3) (*P < 0.05, **P < 0.01, ***P < 0.001, and ****P < 0.0001). **F**, **G** Transmission electron microscope analyses of MCF-7 (**F**) and MDA-MB-453 (**G**) ultrastructures upon Paclitaxel (MCF-7, 3 μM; MDA-MB-453, 5 μM) or SB-T-101141 (MCF-7, 3 μM; MDA-MB-453, 5 μM) treatment for 36 h. **H**, **I** Iron (**H**) and ferrous ions (**I**) detections of MCF-7 cells pretreated with DFOM (50 μM) for 1 h, and then cultured with SB-T-101141 (3 μM) for 24 h. Results were analyzed with the student *t* test (mean ± SD, n = 3). *P < 0.05, **P < 0.01, ***P < 0.001, and ****P < 0.0001. **J** Prussian blue staining of MCF-7 and MDA-MB-453 cells pretreated with DFOM (100 μM) for 1 h and then treated with Paclitaxel (MCF-7, 3 μM; MDA-MB-453, 8 μM) or SB-T-101141 (MCF-7, 3 μM; MDA-MB-453, 8 μM) for 12 h (MCF-7) or 24 h (MDA-MB-453). Bar indicates 100 μm. **K**, **L** MDA (**K**) and GSH (**L**) detection of MCF-7 cells. Cells were treated as (**H**, **I**). Results were analyzed with the student *t* test (mean ± SD, n = 3). *P < 0.05, **P < 0.01, ***P < 0.001, and ****P < 0.0001.

Paclitaxel stabilizes microtubules to lead to cell division halted at the G2 or M phase, resulting in apoptosis [20, 21]. To check whether SB-T-101141 can render apoptosis as like as Paclitaxel, we first performed a cell death analysis and found that SB-T-101141 induced similar apoptosis and G2/M phase arrest of breast cancer cells to Paclitaxel at low concentrations (Fig. S2A–C). However, along with increased concentrations, SB-T-101141 contributed less further increased apoptosis than Paclitaxel, but a nonapoptotic cell death morphology with cell swelling, membrane rupture (Fig. 2A), and increased membrane permeability indicated by trypan blue staining and LDH release, respectively (Fig. 2B–D), without either G2/M phase arrest (Fig. S2D, E) or cleavages of PARP and caspase-7 as apoptotic markers (Fig. S2F, G). To determine the cytotoxic effect derived from the intracellular SB-T-101141 amount, we examined the amounts of SB-T-101141 and Paclitaxel in breast cancer cells and observed lower intracellular accumulation of SB-T-101141 than Paclitaxel (Fig. 2E).

To figure out the distinct mechanism of SB-T-101141 underlying the non-apoptotic cell death, we first utilized an apoptosis inhibitor, Z-VAD-FMK, and necrosis inhibitor, Necrostatin-1, to treat cells in combination with SB-T-101141. Results showed that the cell death and cell growth inhibition by SB-T-101141 were not markedly blocked by these inhibitors (Fig. S3A-C). SB-T-101141 only exhibited a mild effect on mitochondria numbers without significant damage to membrane potential of mitochondria, verified by the Mito-Tracker and JC-10 staining, respectively, compared with Paclitaxel (Fig. S3D–F). Similarly, no notable effect of ATP level characterized as a necroptosis outcome was observed either (Fig. S3G, H), indicating the unique cytotoxic manner of SB-T-101141 on cell death.

**Fig. 3:**
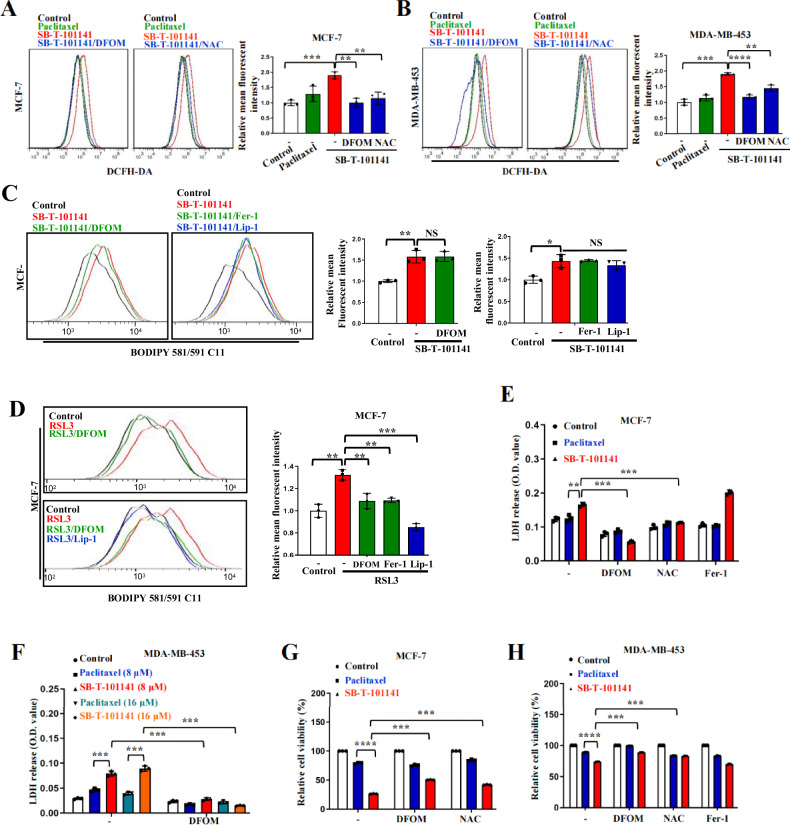
SB-T-101141 induces a noncanonical ferroptosis via iron-dependent ROS. **A**, **B** ROS detection of MCF-7 (**A**, left panel) and MDA-MB-453 (**B**, left panel) cells by a DCFH-DA probe. Cells were pretreated with DFOM (100 μM) or NAC (5 mM) for 1 h, followed by Paclitaxel (MCF-7, 3 μM; MDA-MB-453, 8 μM) or SB-T-101141 (MCF-7, 3 μM; MDA-MB-453, 8 μM) for 3 (MCF-7) or 12 (MDA-MB-453) h. Results were analyzed (MCF-7, **A**, right panel; MDA-MB-453, **B**, right panel) with the student *t* test (mean ± SD, n = 3). *P < 0.05, **P < 0.01, ***P < 0.001, and ****P < 0.0001. **C** Lipid ROS detection of MCF-7 cells with BODIPY 581/591 C11 probe (left panel). Cells were pretreated with DFOM (100 μM), Fer-1 (30 μM), or Lip-1 (5 μM) for 1 h, followed by SB-T-101141 (3 μM) for 16 h. Results were analyzed with the student *t* test (right panel) (mean ± SD, n = 3). *P < 0.05, **P < 0.01, ***P < 0.001, and ****P < 0.0001. **D** Lipid ROS detection of MCF-7 cells with BODIPY 581/591 C11 probe (left panel). Cells were pretreated with DFOM (100 μM), Fer-1 (30 μM), or Lip-1 (5 μM) for 1 h, followed by RSL3 (1 μM) for 16 h. Results were analyzed with the student *t-*test (right panel) (mean ± SD, n = 3). *P < 0.05, **P < 0.01, ***P < 0.001, and ****P < 0.0001. **E**, **F** LDH detection of MCF-7 (**E**) and MDA-MB-453 (**F**) cells. Cells were pretreated with DFOM (100 μM), NAC (5 mM) or Fer-1 (30 μM) for 1 h, followed by Paclitaxel (MCF-7: 3 μM; MDA-MB-453: 8 μM or 16 μM) or SB-T-101141 (MCF-7: 3 μM; MDA-MB-453: 8 μM or 16 μM) for 24 h. Results were analyzed with the student *t* test (mean ± SD, n = 3). *P < 0.05, **P < 0.01, ***P < 0.001, and ****P < 0.0001. **G**, **H** Cell viability analyses of MCF-7 (**G**) and MDA-MB-453 (**H**) cells. Cells were treated with Paclitaxel (MCF-7: 5 μM; MDA-MB-453: 8 μM) or SB-T-101141 (MCF-7: 5 μM; MDA-MB-453: 8 μM) for 36 h (MCF-7) or 24 h (MDA-MB-453) and analyzed using the student *t* test (mean ± SD, n = 3). *P < 0.05, **P < 0.01, ***P < 0.001, and ****P < 0.0001.

Interestingly, in contrast to Paclitaxel, SB-T-101141-treated cells displayed a ferroptosis-like morphology based on the ultrastructures of mitochondrial contraction, increased membrane density, rupture of the outer mitochondrial membrane (OMM), destroyed mitochondrial cristae, and enlarged nuclear, without chromatin condensation (Fig. 2F, G). Ferroptosis induced by ferroptosis agonists erastin and RSL3 occurs in breast cancer with increased ROS, iron, and cell death, consequently breaking lipid membranes and forming lipid peroxidation-derived malondialdehyde (MDA) and 4-hydroxynonenal (4-HNE) regulated by GSH metabolism, and ferroptosis can be in turn blocked by Ferrostain-1 or iron chelator deferoxamine (DFOM) [22]. We thus determined these markers and observed that SB-T-101141 could clearly induce elevated intracellular iron and ferrous ion levels as well as the increased MDA level that could be efficiently attenuated by DFOM (Fig. 2H–K), and reduced GSH level (Fig. 2L), but no obvious effect on GPX4 expression in breast cancer cells (Fig. S3I), which is an enzyme to reduce esterification and oxidation of fatty acids and cholesterol hydroperoxides. Unexpectedly, the increased MDA level caused by SB-T-101141 was not impaired by the ferroptosis inhibitors Ferrostain-1 (Fer-1) and Liproxsrain-1 (Lip-1) (Fig. S3J). DCFH-DA probe-labeled total ROS induced by SB-T-101141 could be efficiently neutralized by DFOM and ROS scavenger N-acetyl-l-cysteine (NAC) (Fig. 3A, B), whereas SB-T-101141-induced intracellular lipid ROS was unable to be attenuated by DFOM, Fer-1, or Lip-1, compared with that induced by the well-known ferroptosis agonist RSL3 (Figs. 3C, D and S3K, L). Consistently, the elevated membrane permeability indicated with LDH release, the impaired cell viability and cell death status induced by SB-T-101141 were markedly attenuated by DFOM and NAC, but not the ferroptosis inhibitors Fer-1 and Lip-1, in comparison with Paclitaxel in various breast cancer cells, respectively (Figs. 3E–H and S4A-G). Furthermore, we observed that in contrast to the ferroptosis agonist RSL3, the reduced cell viability from SB-T-101141 was not reversed by various known ferroptosis inhibitors, except iron chelators DFOM and Ciclopirox (CPX) (Fig. S4H–J). In addition, we also observed that SB-T-101141 evidently suppressed expression of tumor stem-related genes, rather than Paclitaxel (Fig. S4K–L). Therefore, the results here together indicate that SB-T-101141 triggers a novel type of ferroptosis of breast tumor cells.

### SB-T-101141 inhibits Paclitaxel-resistant tumor growth in a noncanonical ferroptosis

**Fig. 4:**
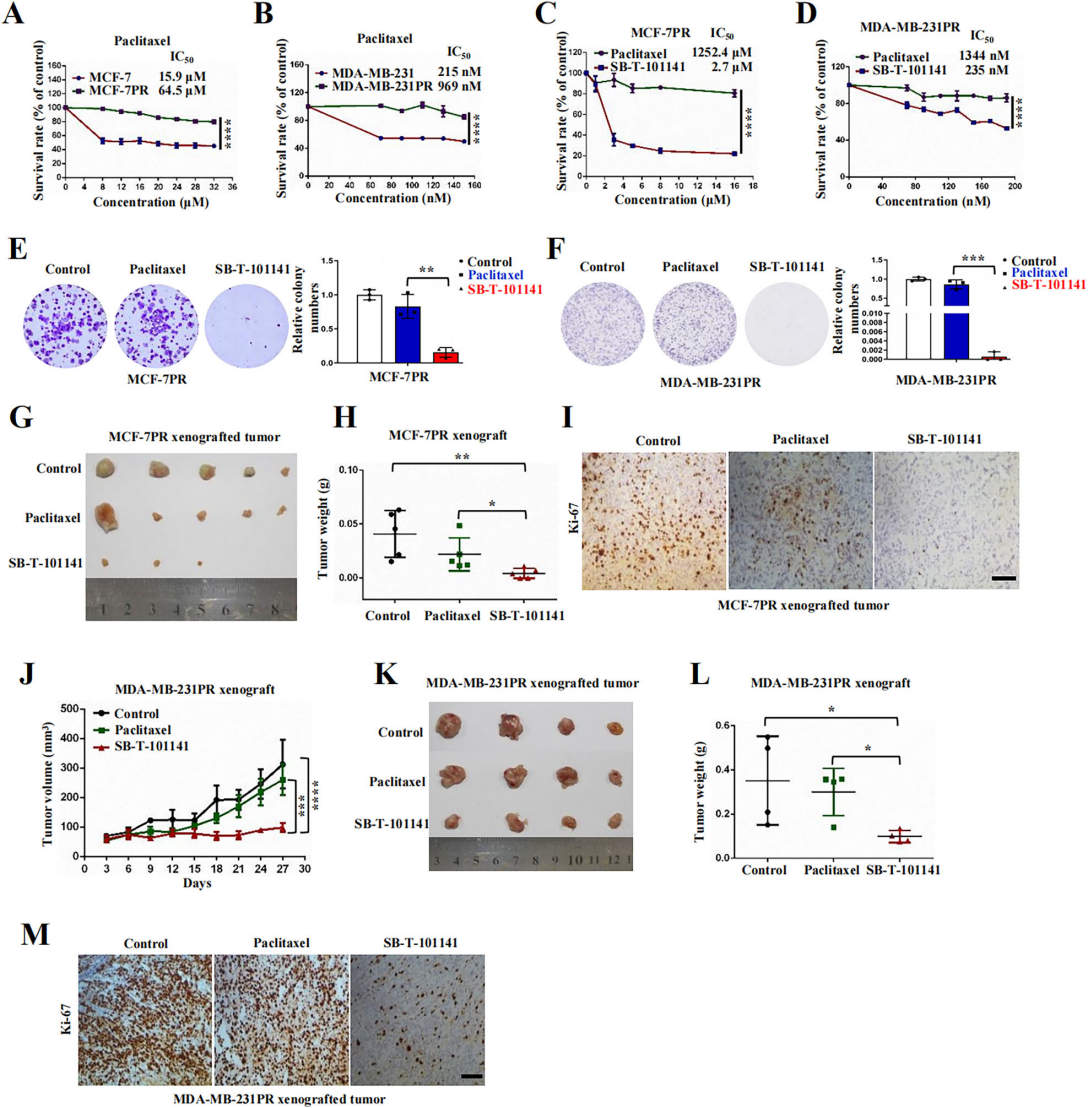
SB-T-101141 represses Paclitaxel-resistant breast tumor growth. **A**–**D** Cell viability analyses of MCF-7 (**A**) and MDA-MB-231 (**B**) with their Paclitaxel-resistant MCF-7PR (**C**) and MDA-MB-231PR (**D**) cells. Cells were treated with different concentrations of Paclitaxel for 72 h. Results were analyzed using a Two-way *ANOVA* test (mean ± SD, n = 3). *P < 0.05, **P < 0.01, ***P < 0.001, and ****P < 0.0001. **E**, **F** Crystal violet staining of formed colonies of MCF-7PR (**E**) and MDA-MB-231PR (**F**) cells (shown in the left panel). Cells were treated with Paclitaxel (MCF-7PR, 3 nM; MDA-MB-231PR, 25 nM) or SB-T-101141 (MCF-7PR, 3 nM; MDA-MB-231PR, 25 nM) for colony formation. Results were analyzed using a student *t* test (mean ± SD, n = 3) (right panel). *P < 0.05, **P < 0.01, ***P < 0.001, ****P < 0.0001. **G**–**I** Xenografted tumor formation of MCF-7PR cells in mice. Mice with xenografted tumors were administrated with Paclitaxel or SB-T-101141. The tumors were dissected and weighed (**G**, **H**) (mean ± SD, n = 4), and analyzed with IHC (**I**) with indicated antibody. *P < 0.05, **P < 0.01, ***P < 0.001, and ****P < 0.0001 (student *t* test). Bar indicates 100 μm. **J**–**M** Xenografted tumor formation of MDA-MB-231PR cells. Mice bearing MDA-MB-231PR xenografts were respectively administrated with Paclitaxel and SB-T-101141. The tumor size (**J**) was monitored (mean ± SD, n = 4) (*P < 0.05, **P < 0.01, ***P < 0.001, and ****P < 0.0001, *ANOVA* test). The tumors were dissected and weighed (**K**, **L**) (mean ±SD, n = 4). *P < 0.05, **P < 0.01, ***P < 0.001, and ****P < 0.0001 (student *t* test). The tumors were subjected to IHC analysis (**M**) with the indicated antibody. Bar indicates 100 μm.

To investigate the effect of SB-T-101141 on Paclitaxel-resistant tumors, we established stable Paclitaxel-resistant breast cancer cell line MCF-7PR by concentration-gradient induction. RNA sequencing indicated that ABC transporter-related pathways were enriched in Paclitaxel-resistant cells, which is associated with multidrug resistance of tumors (Fig. S5A). We then monitored the tolerance of Paclitaxel-resistant MCF-7PR and MDA-MB-231PR cells to Paclitaxel in comparison with their parental cells. Results indicated that MCF-7PR and MDA-MB-231PR cells were highly resistant to Paclitaxel (Fig. 4A, B), but very sensitive to SB-T-101141 indicated with cell survival and colony formation, respectively (Fig. 4C–F). Impressively, SB-T-101141 exhibited a strong antitumor effect on the Paclitaxel-resistant cell xenografted tumor progression in nude mice, without side effects on mouse body weight, compared with Paclitaxel (Figs. 4G–M and S5B). These findings together show a potential strategy to utilize SB-T-101141 to overcome Paclitaxel resistance of breast tumors.

**Fig. 5:**
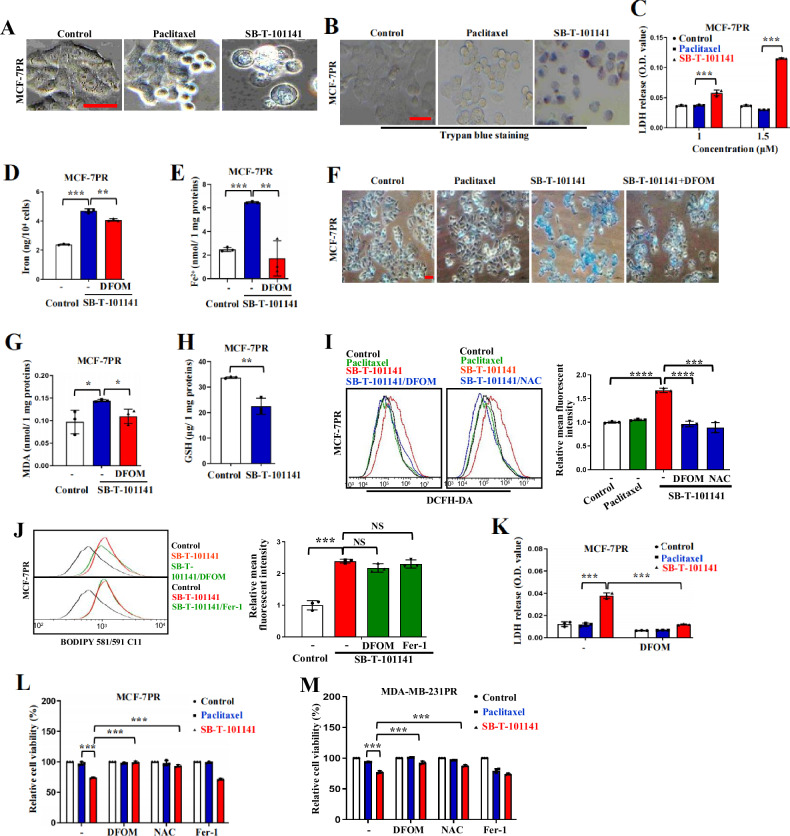
SB-T-101141 induces ferroptotic cell death of Paclitaxel-resistant breast cancer. **A** Cellular morphological analysis of MCF-7PR cells. Cells were respectively treated with Paclitaxel (1.5 μM) or SB-T-101141 (1.5 μM) for 24 h and visualized under a microscope. Bar indicates 100 μm. **B**, **C** Trypan blue staining (**B**) and LDH release (**C**) of MCF-7PR cells. Cells were treated with various concentrations of Paclitaxel (1.5 μM) or SB-T-101141 (1.5 μM) for 48 h. Then the cellular supernatant was collected and detected for LDH release. The results were analyzed using the student *t* test (mean ± SD, n = 3). *P < 0.05, **P < 0.01, ***P < 0.001, and ****P < 0.0001. The cells were stained with trypan blue solution and visualized under a microscope. Bar indicates 100 μm. **D**–**H** Iron (**D**) and ferrous ions (**E**) analyses, Prussian blue staining (**F**), MDA (**G**) and GSH (**H**) detections of MCF-7PR cells pretreated with DFOM (50 μM) for 1 h and then cultured with SB-T-101141 (1.5 μM) for 12 h (**F**) or 24 h (**D**, **E**, **G**, **H**). The results were analyzed using the student *t* test (mean ± SD, n = 3). *P < 0.05, **P < 0.01, ***P < 0.001, and ****P < 0.0001. Bar indicates 100 μm. **I**, **J** Total ROS (**I**, left panel) and lipid ROS (**J**, left panel) detections with either DCFH-DA or BODIPY 581/591 C11 probes of MCF-7PR cells. Cells were pretreated with DFOM (100 μM), NAC (15 mM) or Fer-1 (30 μM) for 1 h, followed by Paclitaxel (1.5 μM) or SB-T-101141(1.5 μM) for 10 h. Results were analyzed (right panel) using the student *t* test (mean ± SD, n = 3). *P < 0.05, **P < 0.01, ***P < 0.001, and ****P < 0.0001. **K**–**M** LDH detection (**K**) and cell viability of MCF-7PR (**L**) or MDA-MB-231PR cells (**M**). Cells were pretreated with DFOM (100 μM), NAC (15 mM), or Fer-1 (30 μM) for 1 h, followed by Paclitaxel (MCF-7PR, 1.5 μM; MDA-MB-231PR, 170 nM) or SB-T-101141 (MCF-7PR, 1.5 μM; MDA-MB-231PR, 170 nM) for 24 h (MCF-7PR) or 48 h (MDA-MB-231PR). Results were analyzed using the student *t* test (mean ± SD, n = 3). *P < 0.05, **P < 0.01, ***P < 0.001, and ****P < 0.0001.

To uncover the mechanism of SB-T-101141 on conquering Paclitaxel resistance, we first checked the microtubule polymerization and cell death of the Paclitaxel-resistant cells and observed that SB-T-101141 could induce microtubule polymerization and promote cell death, but the cell death was not sabotaged by apoptosis inhibitors Z-VAD-FMK and Necrostatin-1 (Fig. S5C, D). We also measured the intracellular SB-T-101141 amount in Paclitaxel-resistant cells by mass spectrometry and found less accumulation of SB-T-101141 than that of Paclitaxel in MCF-7PR cells (Fig. S5E). Moreover, SB-T-101141 caused more prominent membrane rupture and membrane permeability in MCF-7PR cells than Paclitaxel (Figs. 5A–C and S5F). We also observed that SB-T-101141 markedly promoted iron and ferrous ion levels, intracellular iron accumulation, and increased MDA level, which were significantly attenuated by DFOM (Figs. 5D–G and S5G), marked with reduced GSH level (Fig. 5H), but no effect of GPX4 expression in Paclitaxel-resistant breast cancer cells (Fig. S5H). Further study manifested that the increased total ROS from SB-T-101141 was evidently impeded by DFOM and NAC, but not the lipid ROS with DFOM and Fer-1 in Paclitaxel-resistant breast cancer cells (Figs. 5I, J and S5I). Likewise, the increased membrane rupture and reduced cell viability induced by SB-T-101141 were efficiently counteracted by DFOM and NAC, but not ferroptosis inhibitors Fer-1 and Lip-1 (Figs. 5K–M and S5J, K). Unlike ferroptosis agonist RSL3, cell death of the Paclitaxel-resistant cells induced by SB-T-101141 was only efficiently inhibited by iron chelators DFOM and CPX, instead of other various ferroptosis inhibitors (Fig. S5L–O). These findings together show that SB-T-101141 could overcome Paclitaxel resistance in breast tumors by triggering a non-canonical ferroptosis.

### SB-T-101141 suppresses breast tumor growth by binding to KHSRP P572 site

**Fig. 6:**
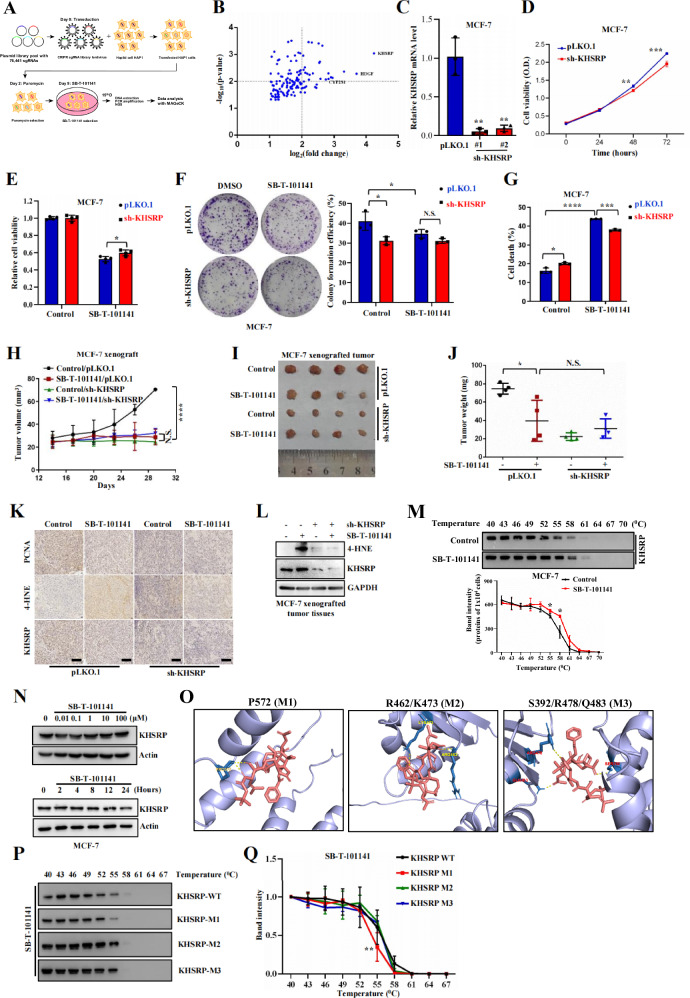
SB-T-101141 inhibits breast tumor growth via KHSRP. **A** Schematic diagram of genome-scale CRISPR-Cas9 knockout screening of the SB-T-101141 target. **B** The volcano plot of the top 133 genes via genome-scale CRISPR-Cas9 knockout screening upon SB-T-101141 treatment. **C** MCF-7 cells were transfected with lentiviruses for knocking down *KHSRP*. The resulting cells were collected and for RNA extraction. Validation of *KHSRP* mRNA expression in stable *KHSRP* knock-down MCF-7 cells. Results were analyzed using student *t* test (mean ± SD, n = 3) (*P < 0.05, **P < 0.01, ***P < 0.001, and ****P < 0.0001). **D** The detection of Cell viability from (**C**) at different time points. Results were plotted and analyzed using student *t* test (mean ± SD, n = 3) (*P < 0.05, **P < 0.01, ***P < 0.001, and ****P < 0.0001). **E**–**G** Cell viability (**E**), colony formation (**F**) and cell death assay with PI staining (**G**) of control MCF-7 cells (pLKO.1) and *KHSRP* knock-down MCF-7 cells (sh-KHSRP) respectively treated with SB-T-101141 (**E**, **G**, 3 μΜ; **F**, 1 nM) for 24 h (**E**, **G**) or 14 days (**F**). Results were analyzed using the student *t* test (mean ± SD, n = 3). *P < 0.05, **P < 0.01, ***P < 0.001, and ****P < 0.0001. **H**–**L** Xenografted tumor formation of MCF-7 cells with *KHSRP* knock-down and their counterparts. Mice with xenografts were administrated with SB-T-101141. The tumor size (**H**) was monitored (mean ± SD, n = 4; *ANOVA* test) and tumors were dissected (**I**) and weighed (**J**) (mean ± SD, n = 4; student *t* test). Tumors were analyzed with IHC (**K**) and IB (**L**) with indicated antibodies. *P < 0.05, **P < 0.01, ***P < 0.001, and ****P < 0.0001; bar indicates 100 μm. **M** Thermal shift assay of KHSRP upon SB-T-101141 binding in MCF-7 cells. Cells were treated with SB-T-101141 (10 μΜ) for 2 h and harvested for IB analysis (top panel) and the intensity of protein bands was measured with densitometry and analyzed with the Two-way *ANOVA* test (bottom panel) (mean ± SD, n = 3). *P < 0.05, **P < 0.01, ***P < 0.001, and ****P < 0.0001. **N** Immunoblots of KHSRP in MCF-7 cells treated with the different concentrations of SB-T-101141 for 24 h (top panel) or SB-T-101141 (1 μΜ) for different time points (bottom panel). **O** Molecular docking of KHSRP with SB-T-101141. The tertiary structure of wild-type or mutant KHSRP was downloaded from the UniProt database (AlphaFoldDB-AF-Q92945-F1), and the docking was performed by AutoDock 4.2. The structure graphics was drawn using PyMOL (version 3.7.7). **P**, **Q** Thermal shift assays of KHSRP mutants in HEK293T cells upon SB-T-101141 (10 μΜ) treatment for 2 h at different temperatures (**P**). KHSRP protein bands intensities (**Q**) were analyzed using the Two-way *ANOVA* test (mean ± SD, n = 3). *P < 0.05, **P < 0.01, ***P < 0.001, and ****P < 0.0001.

To further explore the molecular target of SB-T-101141, we approached the genome-scale CRISPR-Cas9 knockout screening and found the top genes *KHSRP*, *HDGF*, and *CYP2S1* (Fig. 6A, B). Among them, only knocking down *KHSRP* showed more tolerance to SB-T-101141 than knocking down *HDGF* and *CYP2S1* in MCF-7 cells, based on the cell viability, colony formation, cell death and lipid ROS level analyses, despite that knocking down *KHSRP* inhibited the cell growth and survival (Figs. 6C–G and S6A–H). More importantly, we employed the xenografted mice and found that although knocking down *KHSRP* inhibited breast tumor growth, the tumor growth inhibition effect from SB-T-101141 was also abolished in *KHSRP-*depleting cells, indicating that KHSRP acts as at least one of the targets of SB-T-101141 (Figs. 6H–J and S6I). Likely, the increased lipid peroxidation product, aldehyde 4-HNE, was diminished in *KHSRP*-depleted xenografted breast tumors (Figs. 6K, L and S6J).

KHSRP as an RNA-binding protein is involved in pre-mRNA splicing, viral infections, neurons, lipid metabolism, cancer, etc [23]. To dissect the relationship between SB-T-101141 and KHSRP, we conducted cellular thermal shift assay (CETSA) and molecular docking. CETSA is a biophysical assay based on the principle of ligand-induced thermal stabilization of target proteins, which enables direct assessment of drug-target engagement in relevant physiological contexts [24, 25]. Our CETSA showed that SB-T-101141 enhanced the thermal stability of KHSRP protein (Fig. 6M) without influencing KHSRP expression (Fig. 6N), indicating the stable binding between SB-T-101141 and KHSRP. Molecular docking simulation indicated that there are interacting hydrogen bonds between SB-T-101141 and P572, R462, and K473, or S392, R478, and Q483 sites of KHSRP protein (Fig. 6O). To further dissect the specific binding site of KHSRP with SB-T-101141, we carried out serial KHSRP mutants to break the above predicted binding sites of KHSRP (Fig. S6K) and found that only mutation of KHSRP P572 could dramatically attenuate KHSRP thermal stability upon SB-T-101141 treatment, indicating the direct binding of SB-T-101141 with KHSRP at the P572 site (Fig. 6P, Q). These findings together verify that SB-T-101141 induces ferroptosis of breast tumor cells at least via its physical binding with KHSRP.

### SB-T-101141 downregulates the iron homeostasis-related CISD1 expression via KHSRP

**Fig. 7:**
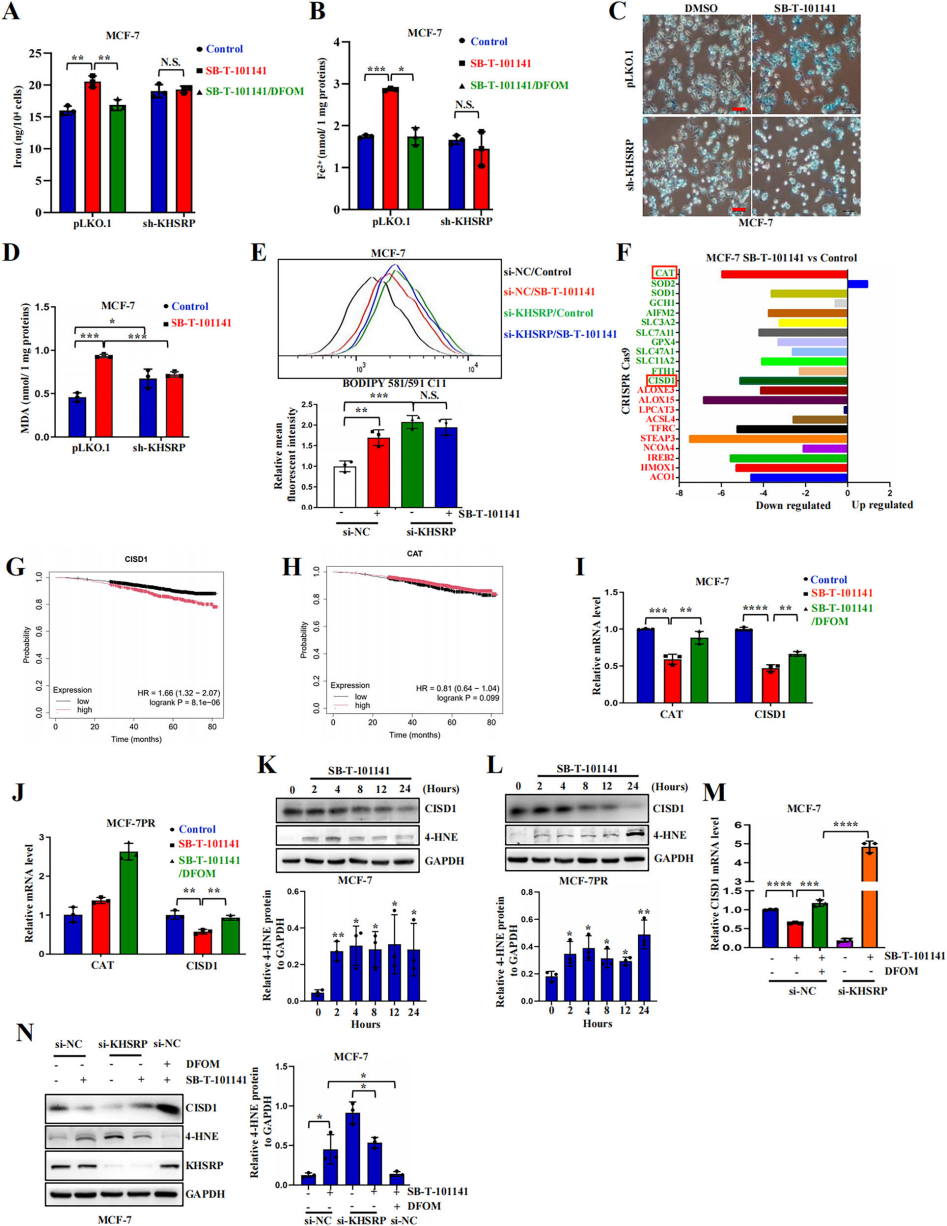
SB-T-101141 promotes lipid peroxidation and inhibits CISD1 expression via KHSRP. **A**–**D** Iron (**A**) and ferrous ions (**B**) detections, Prussian blue staining (**C**), and MDA detection (**D**) in the *KHSRP* knock-down and parental MCF-7 cells. Cells were pretreated with DFOM (50 μΜ) for 1 h, followed by SB-T-101141 (3 μM) treatment for 24 h. Results were analyzed using the student *t* test (mean ± SD, n = 3). *P < 0.05, **P < 0.01, ***P < 0.001, and ****P < 0.0001; bar indicates 100 μm. **E** Lipid ROS detection in MCF-7 cells with the BODIPY 581/591 C11 probes (top panel). Cells were transiently transfected with KHSRP-specific siRNAs (si-KHSRP) for 72 h along with the scramble control (si-NC) and then treated with SB-T-101141 (3 μM) for 16 h. Results were analyzed (bottom panel) using the student *t* test (mean ± SD, n = 3). *P < 0.05, **P < 0.01, ***P < 0.001, and ****P < 0.0001. **F** Analysis of ferroptosis-related genes from the genome-scale CRISPR-Cas9 screening of SB-T-101141 targets in breast cancer cells. The red rectangle indicates the top two genes associated with ferroptosis. **G**, **H** Kaplan–Meier plots of CISD1 (**G**) and CAT (**H**) in the survival of breast cancer patients using the website tool (http://kmplot.com/). The high expression of CISD1 or CAT is in red, and the low expression is in black. **I**, **J** qRT-PCR detection of *CAT* or *CISD1* in MCF-7 (**I**) and MCF-7PR (**J**) cells. Cells were pretreated with DFOM for 1 h, and then with SB-T-101141 (MCF-7: 3 μM; MCF-7PR: 1.5 μM) for 16 h. Results were analyzed using the student *t* test (mean ± SD, n = 3). *P < 0.05, **P < 0.01, ***P < 0.001, and ****P < 0.0001. **K**, **L** Immunoblots of CAT or CISD1 in MCF-7 (**K**, top panel) and MCF-7PR (**L**, top panel) cells treated as (**I**, **J**) at different time points. The results were quantified (bottom panel) and analyzed using the student *t* test (mean ± SD, n = 3). *P < 0.05, **P < 0.01, ***P < 0.001, and ****P < 0.0001. **M** qRT-PCR detection of *CISD1* in *KHSRP* knock-down (si-KHSRP) MCF-7 cells. Cells were transfected with the specific siRNA for 72 h, pretreated with DFOM (100 μM) for 1 h, and then with SB-T-101141 (3 μM) for 16 h. Results were analyzed using the student *t* test (mean ± SD, n = 3). *P < 0.05, **P < 0.01, ***P < 0.001, and ****P < 0.0001. **N** Immunoblots (left panel) of the indicated proteins in *KHSRP* knock-down (si-KHSRP) MCF-7 cells treated as (**M**). The results were quantified (right panel) and analyzed using the student *t* test (mean ± SD, n = 3). *P < 0.05, **P < 0.01, ***P < 0.001, and ****P < 0.0001.

As SB-T-101141 triggered cell death in an iron-dependent manner, we found that SB-T-101141 enhanced cellular iron and ferrous ion levels as well as the MDA and lipid ROS formation, which could be significantly neutralized by *KHSRP* depletion or DFOM in breast cancer cells, despite that knocking down *KHSRP* elevated lipid ROS (Fig. 7A–E), indicating the mediator effector of ferroptosis of KHSRP in SB-T-101141 cytotoxicity. Given the crucial role of iron homeostasis in ferroptosis, we further analyzed the gene expression alteration related to iron homeostasis upon SB-T-101141 treatment with genome-scale CRISPR-Cas9 knockout screening and found that SB-T-101141 inhibited the ferroptosis-resistant gene expression, especially the top two genes, *CISD1* and *CAT* (Fig. 7F). Prognosis analysis of breast cancers showed that *CISD1* expression was negatively associated with clinical outcomes (Fig. 7G, H). CISD1 is a key iron-containing mitochondria outer membrane protein in oxidation modulation and negatively regulates ferroptosis [26, 27], with the function of binding the reactive aldehydes 4-HNE as a redox sensor [28]. We demonstrated that SB-T-101141 effectively downregulated CISD1 at both transcriptional and protein levels, and induced a high level of 4-HNE in both parental and Paclitaxel-resistant cells (Fig. 7I–L). Moreover, decreased CISD1 expression and elevated 4-HNE levels from SB-T-101141 induction were notably abolished by *KHSRP* depletion and DFOM in breast cancer cells (Fig. 7M, N). These findings together implied that SB-T-101141 triggers a novel ferroptosis by binding to KHSRP to repress CISD1 expression.

### SB-T-101141 enhances iron-dependent activation of PERK and JNK pathways by KHSRP

**Fig. 8:**
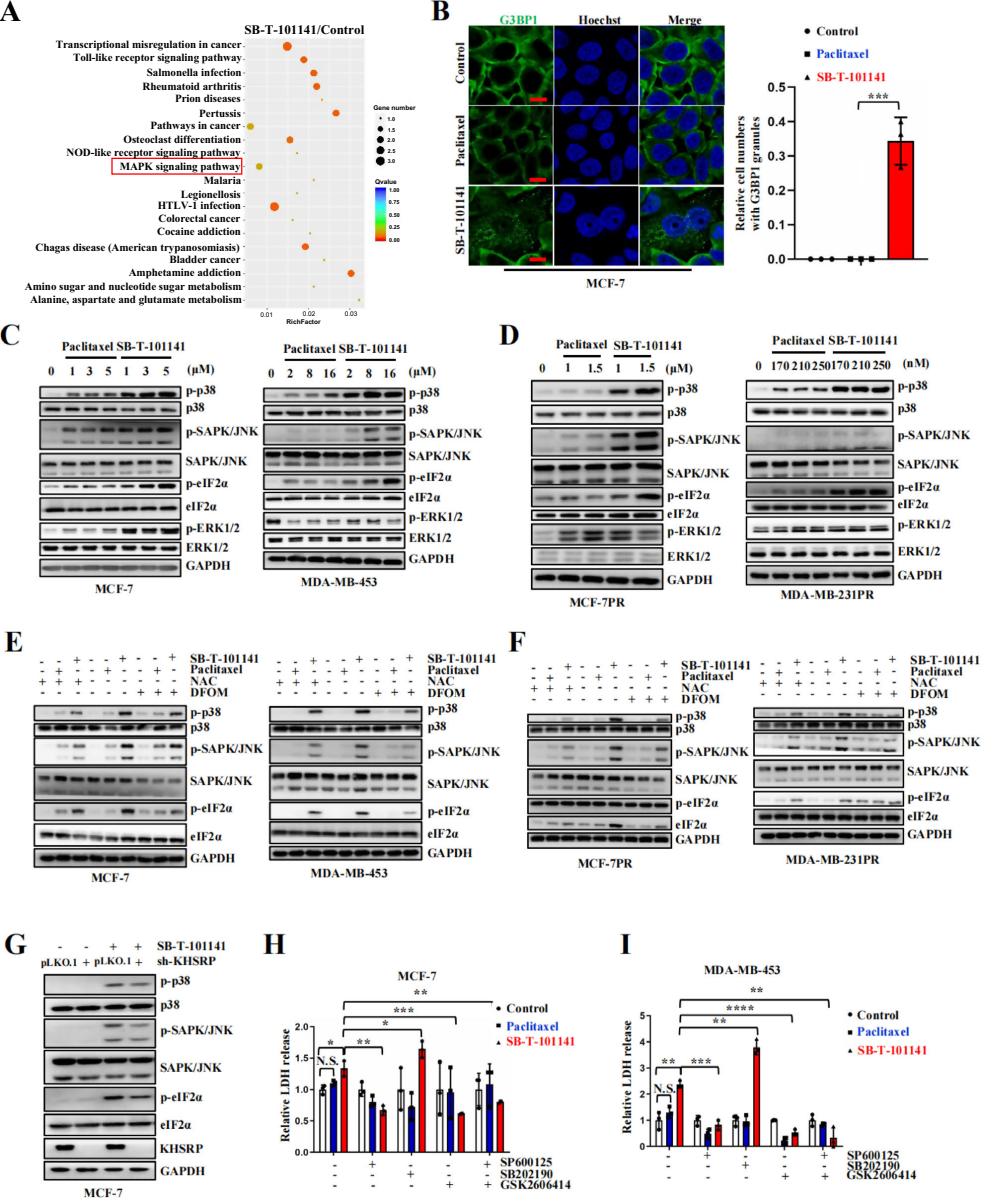
SB-T-101141 induces cell death via the iron-dependent JNK and PERK signaling. **A** The mRNA deep sequencing of MCF-7 upon SB-T-101141 (3 μM) treatment at the indicated time. Red rectangle indicates the MAPK signaling pathway. **B** Immunofluorescence of G3BP1-labeled granules in MCF-7 cells upon Paclitaxel or SB-T-101141 treatment for 4 h (left panel). Bar indicates 10 μm. The results were quantified (right panel) and analyzed using the student *t* test (mean ± SD, n = 3). *P < 0.05, **P < 0.01, ***P < 0.001, and ****P < 0.0001. **C**–**F** Immunoblots of the indicated proteins in MCF-7 and MDA-MB-453 (**C**), MCF-7PR and MDA-MB-231PR (**D**) cells treated with the indicated concentrations of Paclitaxel or SB-T-101141 for 24 h (left panels) or 48 h (right panels). Immunoblots of the indicated proteins in MCF-7 and MDA-MB-453 (**E**), or MCF-7PR and MDA-MB-231PR (**F**) cells. Cells were pretreated with DFOM (**D**, MCF-7PR, 100 μΜ; MDA-MB-231PR, 30 μM) and/or NAC (5 mM) for 1 h, and then with indicated concentrations of Paclitaxel or SB-T-101141 (MCF-7, 3 μM; MDA-MB-453, 8 μM; MCF-7PR, 1.5 μΜ; MDA-MB-231PR, 170 nM) for 24 h (MCF-7PR) or 48 h (MDA-MB-231PR). **G** Immunoblots of MAPK signaling-associated proteins in the *KHSRP* knock-down and its counterpart MCF-7 cells treated with SB-T-101141 (3 μM) for 24 h. **H**, **I** LDH detection of MCF-7 cells (**H**) or MDA-MB-453 (**I**) cells pretreated with SP600125, SB202190 or GSK2606414 for 1 h, followed by Paclitaxel or SB-T-101141 (MCF-7, 3 μM; MDA-MB-453, 8 μM) for 24 h. All LDH release results were analyzed using the student *t* test (mean ± SD, n = 3). *P < 0.05, **P < 0.01, ***P < 0.001, and ****P < 0.0001.

Although KHSRP is a pivotal mediator for SB-T-101141-induced ferroptosis, cell death caused by SB-T-101141 is not completely abolished via *KHSRP* depletion, suggesting that SB-T-101141 would act in multiple ways. We thus conducted an RNA deep sequencing and showed that SB-T-101141 treatment impaired the cancer-related pathways, especially the MAPK pathway (Fig. 8A), suggesting the crucial and complicated roles of KHSRP in SB-T-101141-induced cell death. Thus, we checked the counterpart of MAPK pathway-related cell stress responses and unveiled that SB-T-101141, rather than Paclitaxel, prominently exerted ER stress-related GTPase activating protein (SH3 domain) binding protein 1 (G3BP1) granule aggregation, without affecting G3BP1 expression (Figs. 8B and S7A), suggestion of a partial function of SB-T-101141 on breast tumor cell death via ER stress. ER stress is featured with the formation of G3BP1-containing granules [29], we further evaluated the downstream event of ER stress and observed that SB-T-101141 enhanced phosphorylation of eukaryotic translation initiation factor 2 (eIF2α), a well-known target of the active protein kinase RNA (PKR)-like ER kinase (PERK) upon ER stress [30], along with the JNK/p38MAPK pathway activation in both parental or Paclitaxel-resistant cells, which were efficiently attenuated by DFOM, NAC or *KHSRP* depletion (Figs. 8C–G and S7B, C). Likewise, increased membrane permeability by SB-T-101141 was markedly diminished by the various inhibitors of both JNK and PERK pathways, except the p38MAPK pathway (Figs. 8H, I and S7D–G). These findings indicate that both iron homeostasis and KHSRP play critical roles in SB-T-101141-induced cell death through the activation of JNK and PERK pathways.

## Discussion

Chemoresistance associated with poor response and high tumor relapse is a major obstacle for cancer therapy [31–33]. The innate and acquired resistance to Paclitaxel and other chemo agents is attributed to the dysregulation of signaling pathways, mutations, and epigenetic modulation in breast cancer patients [33–36]. There is an urgent need to develop other agents or strategies [37–39]. Therefore, various taxanes were designed and synthesized based on the structures of the first-generation taxanes, including Paclitaxel and Docetaxel [19, 40, 41]. We previously showed that a taxane derivative presents effective cytotoxicity to Paclitaxel-resistant breast cancer cells, especially the Paclitaxel-resistant TNBC cells [19]. Here, we presented another novel paclitaxel derivative, taxane SB-T-101141, and uncovered its unique ferroptotic effect along with other multiple functions for tumor cell death. Although SB-T-101141 induced microtubule polymerization, even under high concentration, the cell cycle induced by SB-T-101141 was not arrested at G2/M phase, illustrating the extra nonapoptotic effect of SB-T-101141 on cell death. Our results demonstrated that SB-T-101141 presented a better cytotoxic effect on breast cancer cells and similar cytotoxicity with Paclitaxel in non-cancerous human mammary cells. Therefore, chemical modification of chemotherapeutics, including taxanes is still a practical and valuable strategy for developing novel efficient anti-cancer agents.

Apoptosis induced by anti-tumor drugs acts in a major manner in eradicating tumors, nevertheless, acquired resistance to apoptosis in turn becomes a quite common obstacle to chemotherapy. Here, we revealed that SB-T-101141 triggered a novel ferroptotic cell death and thus could efficiently circumvent and overcome Paclitaxel-adapted apoptosis resistance. Therefore, developing novel chemotherapeutic drugs with brand-new cell death induction mechanisms will be a superior strategy to overcome tumor resistance. Meanwhile, we also found that SB-T-101141 evidently inhibited tumor stem-related gene expression. Given that breast cancer stem cells (CSCs) have important roles in breast tumor development, relapse, metastasis, and drug resistance [42], thus the unique effect of SB-T-101141 on eliminating breast CSCs is worth further exploration.

Cells undergoing ferroptosis usually manifest necrosis-like morphological changes [18, 43], featuring the loss of plasma membrane integrity, cytoplasmic swelling (oncosis), swelling of cytoplasmic organelles, and moderate chromatin condensation [18]. Mechanistically, ferroptosis is a ROS-dependent form of cell death accompanied by iron accumulation, lipid peroxidation, and GSH metabolism [18, 22]. Here we showed that SB-T-101141 rendered ferroptosis-like morphological changes in breast cancer cells, with lipid ROS production, iron accumulation, elevation of MDA and 4-HNE levels, and reduced GSH level. However, the lipid-peroxidation product, MDA, and cell death induced by SB-T-101141 could not be blocked by ferroptosis inhibitors Fer-1 and Lip-1, indicating the noncanonical lipid peroxidation process existed in the ferroptotic cells. Thus, the capability and underlying mechanism of SB-T-101141 in inducing noncanonical lipid peroxidation for ferroptosis is worthy of further exploration.

The imbalance of iron metabolism homeostasis is the pivotal initiative in ferroptosis occurrence. Evidence demonstrates that various factors modulate iron metabolism in ferroptosis [18, 44]. Among them, CISD1, an iron-containing protein localized on the outer membrane of mitochondria, plays key roles in regulating ferroptotic cell death and oxidative stress [26, 27, 45]. Generally, CISD1 acts as a redox sensor and the regulator of free iron in mitochondria and binds to 4-HNE produced from the oxidation of polyunsaturated fatty acids upon oxidative stress [28]. Here, we disclosed that SB-T-101141 stably bound to KHSRP, an RNA-binding protein, to regulate CISD1 expression at both transcriptional and translational levels, along with lipid-peroxidation product, 4-HNE, indicating the crucial and unidentified role of KHSRP in iron metabolism and lipid oxidation. CISD1 is highly expressed in breast cancer patients and independently associated with adverse clinical outcomes [46]. Further understanding of the signaling of the KHSRP-CISD1 axis in breast cancer will unveil new targets for ferroptosis induction and novel cancer therapy strategies. Indeed, CISD1 has emerged as the mitochondrial target of thiazolidinedione drugs such as the antidiabetic pioglitazone [47, 48], suggesting the potential therapeutic value of SB-T-101141 for CISD1-positive cancers. Therefore, the interplaying and underlying mechanisms among SB-T-101141, KHSRP-CISD1 axis, and special lipid peroxidation will be further explored.

Our results also showed that SB-T-101141 exerted multiple functions besides KHSRP. Tumor cell survival and death-associated pathways, JNK/p38MAPK and ER stress-related PERK are orchestrated by SB-T-101141 along with iron metabolism in breast cancer cells. ER stress induced by ferroptosis agonists plays a cross-talk role between ferroptosis and other types of cell death [30, 49], and the JNK/p38MAPK signaling influences the susceptibility of cancer cells to ferroptosis [50]. Mechanisms of SB-T-101141 in promoting ER stress and JNK/p38MAPK pathway activation through regulating iron homeostasis and KHSRP will be further dissected. Blocking PERK and JNK pathways attenuated SB-T-101141-induced cell death, while inhibiting the p38MAPK pathway further enhanced SB-T-101141 efficacy in cell death, suggesting the protective function of p38MAPK activation for breast cancer cells to resist SB-T-101141 cytotoxicity. These suggest that the combination of p38MAPK inhibitor and SB-T-101141 will provide a more efficient therapeutic strategy to conquer apoptosis resistance of breast cancer cells or other types of tumor cells via ferroptosis induction, instead of reversing the existing apoptosis resistance.

Taken together, we demonstrated that SB-T-101141 played multiple functions to overcome Paclitaxel resistance, especially through a noncanonical ferroptosis by directly binding to KHSRP to break iron homeostasis. SB-T-101141 will be a novel ferroptosis inducer in both Paclitaxel-resistant cancer therapy and a valuable tool for ferroptosis-related studies.

## Materials and methods

### Reagents

DMEM, RPMI, 0.25% Trypsin, 2.5% Trypsin (EDTA-free), penicillin/streptomycin, and fetal bovine serum (FBS) were purchased from Hyclone. Paclitaxel (catalog no. S1150), Z-VAD-FMK (catalog no. S7023), Liproxstatin-1 (catalog no. S7699), and RSL3 (catalog no. S8155) were purchased from Selleck. SB-T-101141 was synthesized in the laboratory of Dr. Changwei Wang (Guangzhou Institutes of Biomedicine and Health, Chinese Academy of Sciences, China). GSK2606414 (catalog no. HY-18072), SP600125 (catalog no. HY-12041), SB202190 (catalog no. HY-10295), Deferoxamine Mesylate (DFOM) (catalog no. HY-B0988), Ciclopirox (CPX) (catalog no. HY-B0450), Ferrostatin-1 (catalog no. HY-100579), Zileuton (catalog no. HY-14164), Baicalein (catalog no. HY-N0196) and Estradiol cypionate (catalog no. HY-B1100) were purchased from MCE. N-Acetyl-L-cysteine (NAC) (catalog no. A9165), Prussian blue soluble (catalog no. 03899), dimethyl sulfoxide (DMSO) (catalog no. D2650), absolute ethyl alcohol (catalog no. E7023), Kolliphor® EL (catalog no. C5135) and Trypan blue (catalog no. T0776) were purchased from Sigma-Aldrich. Crystal violet (catalog no. 0528) was purchased from Amresco. Cell Counting Kit-8 (CCK-8) (catalog no. CK04) was purchased from Dojindo (Japan). CytoTox 96® Non-Radioactive Cytotoxicity Assay (catalog no. G1780) was purchased from Promega. BODIPY 581/591 C11 (catalog no. D3861) was purchased from Invitrogen. Mito Tracker green (catalog no. BB-41293-1), DCFH-DA (catalog no. BB-47053-1) and JC-10 (catalog no. BB-41052-2) were purchased from Bestbio (China).

### Antibodies

Anti-β-Tubulin antibody (catalog no. 2146), anti-eIF2α antibody (catalog no. 5324), anti-phospho-eIF2α antibody (catalog no. 3398), Phospho-MAPK Family Antibody Sampler Kit (catalog no. 9910), MAPK Family Antibody Sampler Kit (catalog no. 9926), anti-GAPDH antibody (catalog no. 2118), anti-PARP antibody (catalog no. 9542), anti-Caspase-7 antibody (catalog no. 9492), anti-Cyclin B1 antibody (catalog no. 12231), anti-Cdc25c antibody (catalog no. 4688), anti-Cyclin D1 antibody (catalog no. 55506), anti-Cyclin D3 antibody (catalog no. 2936), anti-CDK4 antibody (catalog no. 12790), anti-CDK6 antibody (catalog no. 13331), anti-CDK2 antibody (catalog no. 2546), anti-P21 antibody (catalog no. 2947), anti-P27 antibody (catalog no. 3686), anti-mouse IgG, HRP-linked antibody (catalog no. 7076) and anti-rabbit IgG and HRP-linked antibody (catalog no. 7074) were obtained from Cell Signaling Technology. Anti-KHSRP antibody (catalog no. ab150393), anti-4-HNE antibody (catalog no. ab48506), and anti-PCNA antibody (catalog no. ab29) were purchased from Abcam. Anti-Actin antibody (catalog no. 100166-MM10) was purchased from Sino Biological. Anti-CISD1 antibody (catalog no. sc-517413), anti-G3BP1 antibody (catalog no. sc-365338), and anti-GPX4 antibody (catalog no. sc-166570) were obtained from Santa Cruz Biotechnology. Anti-Cdc2 antibody (catalog no. 610037) was obtained from BD Biosciences. Alexa Fluor 488 donkey anti-mouse IgG (H + L) (catalog no. A-21202) and Alexa Fluor 488 donkey anti-rabbit IgG (H + L) (catalog no. A-21206) were obtained from Life Technologies. Anti-Flag antibody (catalog no. F9291) was obtained from Sigma.

### Establishment of the Paclitaxel-resistant cell line MCF-7PR

Paclitaxel-resistant MCF-7PR cell line was established from its drug-sensitive parental cell line MCF-7 with a stepwise increase of Paclitaxel concentration, including 0.5, 1, 2, 4, 6, 8, 10 nM. The cells were cultured in DMEM supplemented with 10% FBS and penicillin/streptomycin (100 U/ml/50 μg/ml) at 37 °C and 5% CO_2_, containing Paclitaxel in each step for at least 2 weeks with exchanging medium every 2 days. The resistant cell line was established after the continuous induction with Paclitaxel for 12 months. The resulting Paclitaxel-resistant MCF-7 cell line, designated MCF-7PR, was maintained in medium with 10 nM Paclitaxel at alternate passaging. The MCF-7PR cell line was cultured in Paclitaxel-free medium for at least one passage before being used for experiments.

### Cell culture

MDA-MB-453 and MDA-MB-231 were purchased from the American Tissue Culture Collection (ATCC). MCF-7 was a gift from the laboratory of Prof. Jing Tan (Sun Yat-sen University Cancer Center, Guangzhou, China). The Paclitaxel-resistant breast cancer cell line MCF-7PR was established in our laboratory. MDA-MB-231PR was a gift from the laboratory of Prof. Qiang Yu (Genome Institute of Singapore, GIS). HAP1 was a gift from the laboratory of Prof. Qiaoping Wang (School of Pharmaceutical Sciences, Sun Yat-sen University, Shenzhen, China). MCF-7 and MDA-MB-453 cells were cultured in RPMI supplemented with 10% FBS and penicillin/streptomycin (100 U/ml/50 μg/ml) at 37 °C and 5% CO_2_. MDA-MB-231 cells were cultured in DMEM supplemented with 10% FBS and penicillin/streptomycin (100 U/ml/50 μg/ml). MCF-7PR and MDA-MB-231PR cells were cultivated in DMEM supplemented with 10% FBS and penicillin/streptomycin (100 U/ml/50 μg/ml) containing 10 nM and 75 nM Paclitaxel, respectively, at 37 °C and 5% CO_2_. HAP1 cells were cultured in Iscove’s Modified Dulbecco’s Medium (IMDM; 12440053, Gibco) with 10% Fetal Bovine Serum and 1% Penicillin/Streptomycin.

### Measurement of cell growth or cytotoxicity and cell death

Cell proliferation or cytotoxicity was measured by CCK-8 assay according to the manufacturer’s instructions. Cells were seeded in a 96-well plate and incubated with indicated chemical compounds at indicated concentrations for the indicated time. The spectrophotometric absorbance was determined by a microplate reader (Tecan, Switzerland) at 450 nm. Cell death was measured using an Annexin V-FITC/PI Apoptosis Detection Kit (catalog no. A211-01, Vazyme, China) or Annexin V-APC/PI Apoptosis Detection Kit (catalog no. A214-01, Vazyme, China) according to the manufacturer’s instructions. Cells were seeded in a 6-well plate and treated with various chemical compounds at indicated concentrations for the indicated time. Then, cell death was detected using a CytoFLEX S flow cytometer (BECKMAN COULTER, CA, USA).

### Immunofluorescence

The cells were inoculated on the coverslips in a 12-well plate and treated with indicated chemical compounds for indicated time points. The detailed procedures were carried out as the previously described [51]. The resulting cells were incubated with the primary antibodies, including anti-β-tubulin (1:200), anti-G3BP1 antibody (1:200), anti-phospho-JNK antibody (1:200) and anti-phospho-P38 antibody (1:200), or stained with Mito-Tracker Red (catalog no. C1049B, Beyotime, China). All images were collected with a Nikon Eclipse Ni-E confocal laser scanning microscope with the same parameter settings in each experiment.

### Cell cycle

The cells were seeded in 6-well plates and incubated with different chemical compounds for 24 h. Then the cells were treated according to the manufacturer’s protocols of the Cell Cycle Assay Kit (catalog no. BB-4104-3, BestBio, China). The cell cycle was measured using a CytoFLEX flow cytometer (BECKMAN COULTER).

### EdU labeling assay

The cells on the coverslips were cultured with the medium containing 25 μM EdU and indicated chemical compounds. At the indicated time points, the resulting cells were operated per the manufacturer’s instructions for Cell-Light EdU Apollo643 In Vitro Kit (catalog no. C10310-2, Ribobio, China). All images were collected with a Nikon Eclipse Ni-E confocal laser scanning microscope with the same parameter settings in each experiment.

### Colony formation

Cancer cells were seeded in a 6-well plate (1000 cells per well) and incubated with indicated chemical compounds. After 14 days, the colonies were stained with crystal violet after fixation. Then the number of colonies was counted by the GS-800 system (BIO-RAD, CA, USA) and analyzed using Image J software.

### Trypan blue staining

The cells were inoculated in the 12-well plates and cultured with different chemical compounds. At the indicated time points, the cells were incubated for 5 min at RT with an equal volume of 0.4% trypan blue solution and washed twice with PBS. All images were collected with a Nikon ECLIPSE Ti fluorescent microscope.

### LDH measurement

The cells were inoculated in the 96-well plates and incubated with different chemical compounds. At the indicated time points, the 50 μl supernatants from all testing and control wells were transferred to a new 96-well flat clear bottom plate and added with 50 μl of the CytoTox 96® Reagent at RT, avoiding light for 30 min. Then each well was added with 50 μl Stop Solution. The spectrophotometric absorbance was determined by a microplate reader (Tecan, Switzerland) at 490 or 492 nm.

### Measurement of intracellular drug concentration

The detection of intracellular Paclitaxel and SB-T-101141 was performed according to the previously described [19]. Briefly, MCF-7, MDA-MB-453, or MCF-7R cells were seeded into 60 mm plates at 3 × 10^6^ cells/well. The cells were treated with Paclitaxel or SB-T-101141 for 4 h. Cells were harvested, and intracellular compounds were measured using TSQ Quantum Ultra (Thermo, USA). Data were processed using Thermo Xcalibur software. The standard curves of each drug concentration were established based on the area under the typical drug mass detection peak (Y-axis: m/z at 854 for Paclitaxel; m/z at 814 for SB-T-101141) to the serial drug concentration (X-axis). The total intracellular drug amount was calculated by multiplying the detected drug concentration with the sample volume and dilution ratio.

### Ultrastructural analysis

Transmission electron microscopy (TEM) was used to observe the morphologies and sizes of mitochondria. The cells were treated with different chemical compounds at the indicated time points and then fixed using 2.5% glutaraldehyde. Gradient dehydration using alcohol and post-fixation using 1% osmium tetroxide were completed before embedding in resin (Epon 812). Ultrathin sectioning and double staining using uranyl acetate and lead citrate were employed to prepare TEM samples. Finally, the samples were analyzed using a transmission microscope (Tecnai 12; FEI, Lausanne, Switzerland).

### MDA assay

The MDA assay was performed using a Micro Malondialdehyde (MDA) Assay Kit (SH113W-100, G-CLONE, China) according to the manufacturer’s instructions. Cells were treated with indicated chemical compounds for 24 h and harvested by tryptic digestion. The pellet of 5 × 10^6^ cells was resuspended in the extracting solution at 1 ml volume, followed by ultrasonication and centrifuged at 4 °C. The supernatant was collected, and the absorbance at a wavelength of 600 nm was detected using a microplate reader.

### Iron/Fe^2+^ measurement

Cellular iron and Fe^2+^ measurements were performed by Cell Iron Content Assay Kit (BC5315, Solarbio, China) and Ferrous Ion Content Assay Kit (BC5415, Solarbio, China) according to the manufacturer’s instructions, respectively. Cells were treated with indicated chemical compounds for 24 h and harvested by tryptic digestion. The pellet of 10^7^ cells was resuspended in the reagent I at 1 ml volume, followed by ultrasonication and centrifuged at 4 °C. The supernatant was collected for the detection of iron or Fe^2+^ content, and the absorbances at the wavelengths of 510 nm or 593 nm were determined using a microplate reader.

### GSH measurement

GSH detection was performed via Reduced Glutathione (GSH) Content Assay Kit (BC1175, Solarbio, China) according to the manufacturer’s instructions. Cells were treated with indicated chemical compounds for 24 h and harvested by tryptic digestion. The pellet of 10^7^ cells was resuspended in the reagent I at 1 ml volume, followed by freeze-thaw using liquid nitrogen and 37 °C water bath, and centrifuged at 4 °C. The supernatant was collected, and the absorbance at a wavelength of 412 nm was determined using a microplate reader.

### Prussian blue staining

The cells were treated with different chemical compounds. Then the cells were fixed in 4% paraformaldehyde for 30 min and then were washed 3 times with PBS. Next, the cells were incubated with Prussian blue (10 mg/ml) for 30 min and then washed three times with PBS. Labeled cells were examined under a light microscope to determine intracellular iron oxide distribution.

### Measurement of ROS, MTP, and mitochondrial mass

The cells were treated with different chemical compounds. At the indicated time points, cells were collected and stained with different probes according to the manufacturer’s instructions, and analyzed using CytoFLEX flow cytometer (BECKMAN COULTER). Cellular ROS levels were detected with the probes of DCFH-DA and BODIPY 581/591 C11. MTP was detected with a JC-10 probe. The mitochondrial mass was analyzed by the probe of Mito Tracker Green.

### ATP measurement

The detection of ATP levels was performed via the Enhanced ATP Assay Kit (Beyotime, S0027) according to the manufacturer’s instructions. Cells were lysed in ATP lysis buffer, and the supernatant was remained. ATP testing agent was added to the supernatant, and then the RLU value was detected using a luminometer.

### Genome-scale CRISPR-Cas9 knockout screening

To ensure that most cells received only one stably integrated RNA guide, the lentiviral library was transduced at a low MOI (approximately 0.3). HAP1 cells at a density of 70–80% were transduced with lentiviral library produced as described above and polybrene (working concentration 8 μg/ml), and the medium was changed with IMDM complete medium 12 h post-transduction. After cultured for 24 h, the HAP1 cells were added with puromycin (1 μg/ml) and maintained for 7 days. At the same time, 3.82 × 10^7^ cells were passaged as a group to maintain the integrity of the lentiviral library. Transduced HAP1 cells were treated with SB-T-101141 (15 nM) and equivalent DMSO as control. Two replicates were for each group. The cell pools were passaged or changed with fresh medium every 2–3 days. After being treated for 15 days, living cells were harvested for genomic DNA sequencing. Genomic DNA was extracted using TIANamp Genomic DNA kits (DP304, TIANGEN) and amplified using High-Fidelity 2×PCR Master Mix (M0541L, NEB) according to the manufacturer’s instructions. PCR products were purified through agarose gel electrophoresis and gel extraction, and then sequenced using Illumina (PE150) by Novogene Technology (Beijing, China). The enrichment of sgRNAs and genes was analyzed using MAGeCK (Version 0.5.9.2).

### ShRNAs, siRNAs, and plasmids

Short hairpin RNAs (shRNAs) by targeting the *KHSRP*, *HDGF*, or *CYP2S1* transcript were inserted into the vector pLKO.1. The target sequences were shown in Supplementary Table S1, which were the same as the sequences targeted by small interfering RNAs (siRNAs) synthesized by Suzhou Genepharma Co., Ltd (China). The fragments *KHSRP* and mutants were respectively cloned and inserted into the vectors pcDNA3.1. All plasmids were generated using the QuikChange XL Site-Directed Mutagenesis Kit (Stratagene) according to the manufacturer’s instructions.

### Quantitative real-time PCR

After treatment with various chemical compounds for indicated time points, cells were collected in Trizol reagent and total RNA was extracted. The reverse transcription was carried out following the manufacturer’s protocols using HiScript II Q RT SuperMix for qPCR (+gDNA wiper) (catalog no. R223-01, Vazyme, China). Then, quantitative real-time PCR was performed using ChamQ SYBR qPCR Master Mix (catalog no. Q311-02, Vazyme, China) by a real-time PCR measurement system CFX96 touch (Bio-Rad, CA, USA). The primers were shown in Supplementary Table S2.

### Western Blot

Cells treated with different chemical compounds at the indicated time points were washed twice with PBS and collected. The pelleted cells were lysed on ice for 30 min in RIPA lysis buffer (catalog no. P0013B, Beyotime) supplemented with phosphatase inhibitor cocktail tablets (one tablet/10 ml; catalog no. 26920800, Roche), 1 mM PMSF (catalog no. B111-01, GeneStar). Protein bands were visualized with a chemiluminescence system (ECL; catalog no. WBKLS0500, Millipore) according to the manufacturer’s instructions using the ChemiDoc^TM^ Touch imaging system (BIO-RAD).

### Molecular docking

The tertiary structure of KHSRP was downloaded from the UniProt database (AlphaFoldDB-AF-Q92945-F1). Molecular docking of KHSRP and SB-T-101141 was done by AutoDock 4.2. KHSRP was used to be the receptor after adding all hydrogens, and SB-T-101141 was regarded as the ligand after energy minimization and adding all hydrogen. The docking procedure molecular interaction analyses were referred to in the official guide of AutoDock. The structure graphics was done using PyMOL (version 3.7.7).

### Cellular thermal shift assay (CETSA)

The detailed experimental procedures were described as previously [52]. MCF-7 cells were cultured at a density of 80% in a 10 cm dish and treated with SB-T-101141 (10 μM) or equivalent DMSO for 2 h. Three replicates were for each group. The resulting cells were collected and washed using PBS and then resuspended in 1 ml PBS. Cells were divided equally for 10 parts, following heated with the temperature gradient (40–67 °C) for 5 min in the Veriti 96-well thermal cycler (4375303, Applied BiosystemsTM), incubated at RT for 5 min and snap-freezing in liquid nitrogen. The heat-treated cells were lysed with freezing-thawing cycles with liquid nitrogen and a thermal cycler set at 25 °C. The cell lysate was centrifuged at 20,000 × *g* for 20 min at 4 °C. The supernatant was mixed with the reducing loading buffer and heated at 70 °C for 10 min. Prepared samples were detected via western blot and analyzed using Image J software.

### Immunohistochemistry (IHC)

The detailed procedures were performed as previously reported [51]. The sections of xenografted tumor tissues were incubated with an anti-Ki-67 antibody, anti-PCNA antibody, anti-4-HNE antibody and anti-KHSRP antibody.

### Establishment and viability detection of patient-derived breast cancer organoids

Breast cancer organoids were established by using the Breast Cancer Organoid Kit (K2147-BC, bioGenous, Suzhou, China) following the manufacturer’s protocol. Briefly, a small portion of human breast cancer tissues were collected from the First Affiliated Hospital of Sun Yat-sen University in compliance with the medical ethical standards and procedures and sliced into small pieces with scalpels. The small tissues were digested and filtered, and then removed the red blood cells. The resulting cells were cultured in the matrigel with the organoid culture medium. The medium was changed every 3–4 days until the formation of patient-derived breast cancer organoids. Organoids were seeded into 96-well plates and treated with the indicated concentration of Paclitaxel and SB-T-101141 for the shown periods. The treated organoids were examined using the kit of CellTiter-Glo Luminescent Cell Viability Assay (G7571, Promega) following the manufacturer’s instructions and analyzed by Varioskan LUX Multimode Microplate Reader (Thermo Scientific).

### Xenografted mouse assays

The 4-week-old female immune-deficient BALB/c nude mice (Beijing Vital River Laboratory Animal Technology Co., Ltd, China) were quarantined for a week. Before injection with breast cancer cells, the nude mice were subcutaneously injected with estradiol cypionate (1.5 mg/kg). Estradiol cypionate was administered once every 7 days until the experimental endpoint. The 5 × 10^6^ MCF-7 cells or 1 × 10^7^ MDA-MB-453 cells were resuspended in the mix of 50% PBS and 50% matrigel (catalog no. 354262, BD) at a volume of 0.15 ml, respectively, then were subcutaneously injected to the flank of nude mice. Both Paclitaxel and SB-T-101141 were formulated in 50% absolute ethyl alcohol and 50% Kolliphor® EL at a concentration of 30 mg/ml. Until palpable MCF-7 or MDA-MB-453 xenografted tumor formation, the xenografted mice were administered intraperitoneally with Paclitaxel and SB-T-101141 once every three days at a dose of 5 mg/kg, respectively. The 6 × 10^6^ *KHSRP* knock-down MCF-7 cells and wild-type MCF-7 cells were subcutaneously injected into the flank of nude mice. Until the palpable xenografted tumor formation, the xenografted mice were administered intraperitoneally with SB-T-101141 once every three days at a dose of 5 mg/kg. The body weight of mice and tumor volume, which was calculated as 0.5 × length × width^2^, were measured once every 3 or 4 days. At the experimental endpoints, mice were euthanatized and tumor weights were measured after resection. All animal experiments were proved by the Institutional Animal Care and Use Committee, Sun Yat-sen University.

### Quantification and statistical analyses

GraphPad Prism version 8.0 and SPSS statistic 19.0 were used for statistical analyses. For all experiments, data were analyzed by a Two-tailed Student’s *t* test or Two-way analysis of variance (*ANOVA*) test. N represents repeats of the experiment. Results were considered significant at *P < 0.05, **P < 0.01, ***P < 0.001, and ****P < 0.0001.

### Supplementary information

Supplementary information, including supplemental Figs. S1–7 (PDF format) and supplementary Tables 1–2 (Docx format), respectively.

## Supplementary information

Supplementary Figures 1–7 and supplementary Tables 1–2

Original Data

## Data Availability

The original uncropped western blots and quantitative RT-PCR data are supplied in the Original Data file, which can be viewed online, respectively. Genome-scale CRISPR-Cas9 knockout screening data (bioProject accession: HRA010857) have been submitted to GSA-Human database.
