# Supplementary material for: A novel taxane SB-T-101141 triggers a noncanonical ferroptosis to overcome Paclitaxel resistance of breast cancer via iron homeostasis-related KHSRP
Source: Cell Death Dis. 2025 May 19;16(1):403. doi: 10.1038/s41419-025-07710-0 (PMC12089390; doi:10.1038/s41419-025-07710-0)
Supplement: Supplementary file 1 — Supplementary Figures 1–7 and supplementary Tables 1–2 [file 41419_2025_7710_MOESM1_ESM.pdf]

Supplemental Figures 1-7

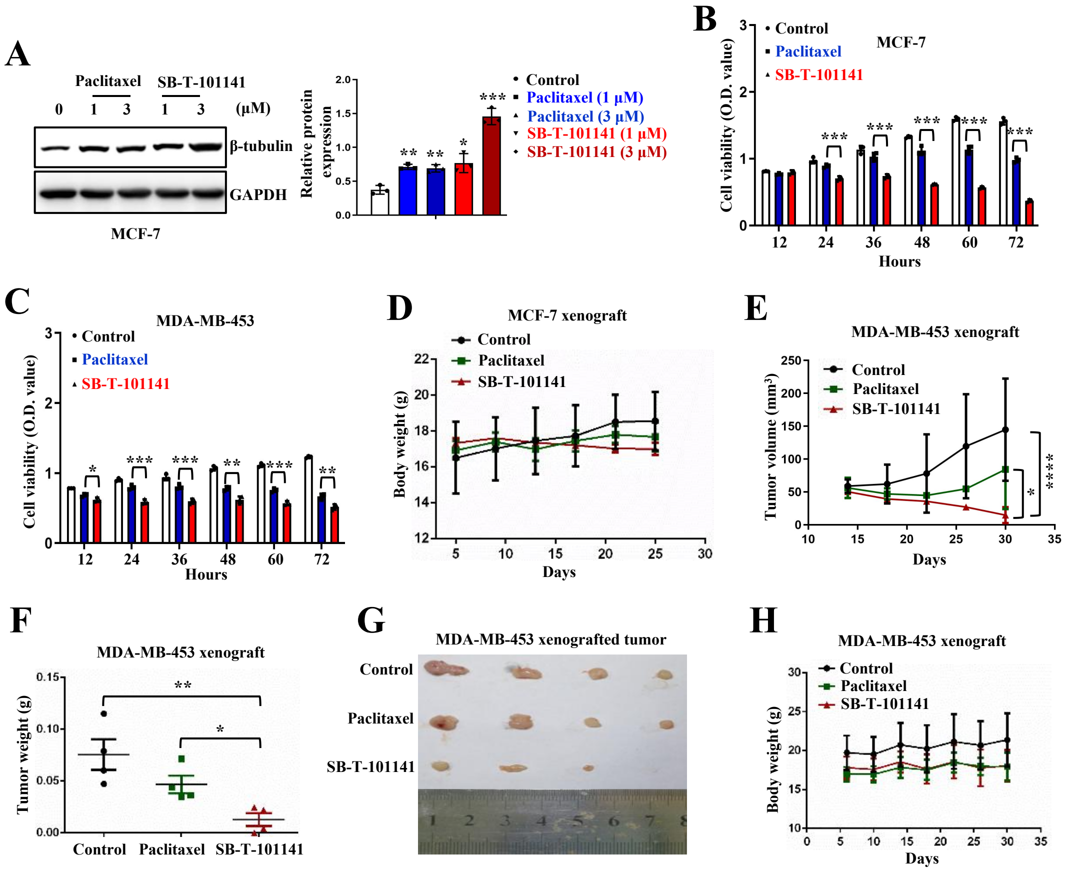

Figure S1. SB-T-101141 markedly represses tumor growth

(A) Immunoblots of the indicated proteins in MCF-7 cells treated with different concentrations of Paclitaxel and SB-T-101141 for 12 hours, respectively (left panel). The results were quantified (right panel) and analyzed using the student *t*-test (mean+/-SD, n = 3). *P < 0.05, **P < 0.01, ***P < 0.001, and ****P < 0.0001. **(B, C)** Cell viability analysis of MCF-7 (B) and MDA-MB-453 (C) cells respectively treated with Paclitaxel (MCF-7, 3 μM; MDA-MB-453, 8 μM) and SB-T-101141 (MCF-7, 3 μM; MDA-MB-453, 8 μM) at different times. Results were analyzed using the student *t*-test (mean+/-SD, n = 3) (*P < 0.05, **P < 0.01, ***P < 0.001, and ****P < 0.0001). **(D)** Xenograft tumorigenesis of MCF-7 cells in nude mice. Mice bearing xenografts were individually treated with Paclitaxel or SB-T-101141. The body weight of mice was monitored (mean+/-SD, n = 4) (*P < 0.05, **P < 0.01, ***P < 0.001, and ****P < 0.0001, *ANOVA* test). **(E-H)** Xenograft tumorigenesis of MDA-MB-453 cells. Mice bearing MDA-MB-453 xenografts were treated with Paclitaxel or SB-T-101141. The tumor size (E) and body weight of mice (H) were monitored (mean+/-SD, n = 4) (*P < 0.05, **P < 0.01, ***P < 0.001, and ****P < 0.0001, *ANOVA* test). The tumors were dissected and weighed (F, G) (mean+/-SD, n = 4). *P < 0.05, **P < 0.01, ***P < 0.001, and ****P < 0.0001 (student *t* test).

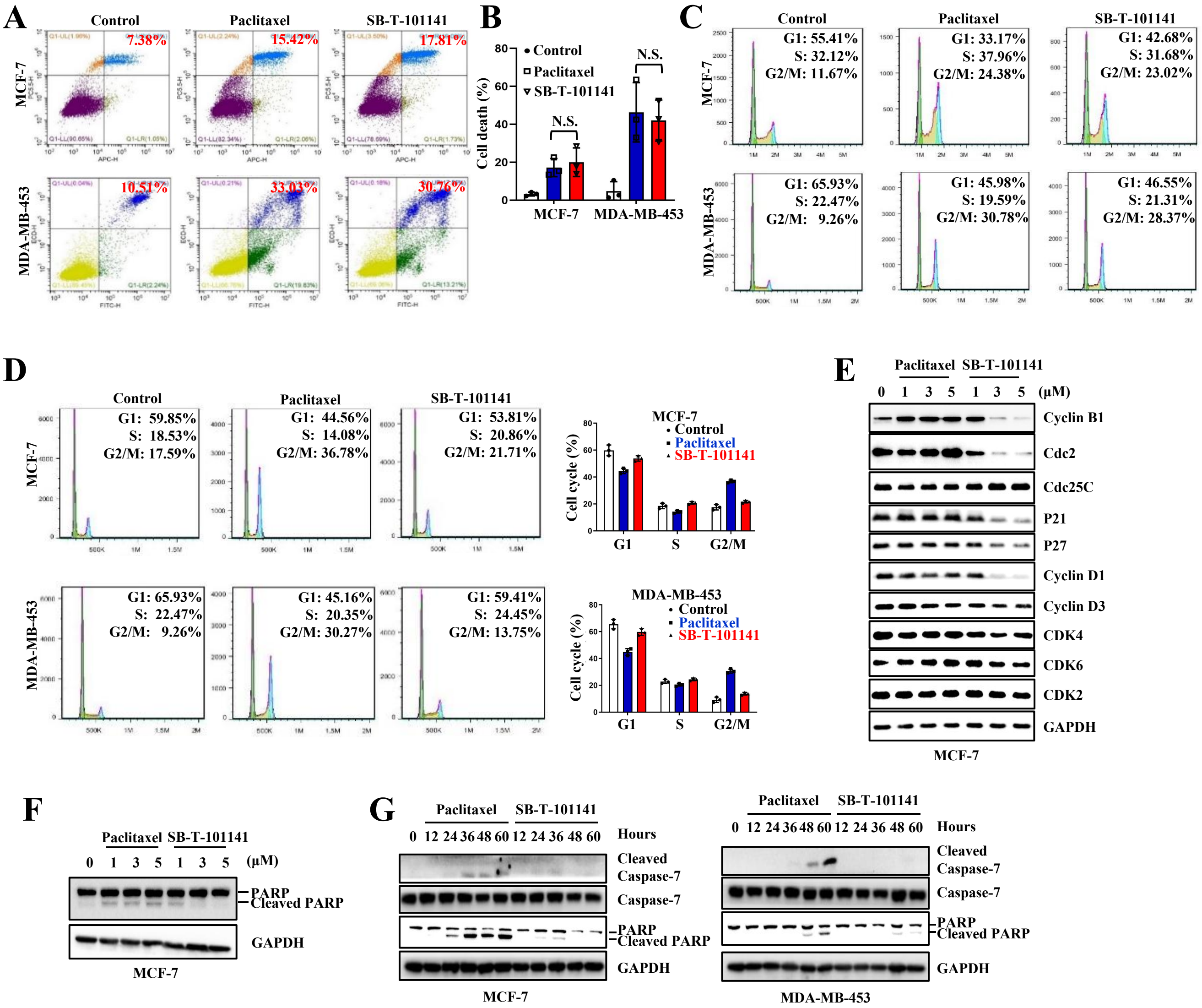

Figure S2. SB-T-101141 has no facilitating role in inducing apoptosis in breast cancer cells compared with Paclitaxel

(A) Cell apoptosis analyses of MCF-7 and MDA-MB-453 cells treated with Paclitaxel (MCF-7, 1 μM; MDA-MB-453, 1 μM) and SB-T-101141 (MCF-7, 1 μM; MDA-MB-453, 1 μM) for 48 hours, respectively. **(B)** The results (A) were quantified and analyzed using the student *t*-test (mean+/-SD, n = 3). *P < 0.05, **P < 0.01, ***P < 0.001, and ****P < 0.0001. **(C)** Cell cycle analyses of MCF-7 and MDA-MB-453 cells treated with Paclitaxel (MCF-7, 1 μM; MDA-MB-453, 1 μM) and SB-T-101141 (MCF-7, 1 μM; MDA-MB-453, 1 μM) for 24 hours, respectively. **(D)** Cell cycle analyses (left panel) of MCF-7 and MDA-MB-453 cells individually treated with Paclitaxel (MCF-7, 3 μM; MDA-MB-453, 8 μM) and SB-T-101141 (MCF-7, 3 μM; MDA-MB-453, 8 μM) for 24 hours. The histogram is plotted (right panel). **(E)** Immunoblots of the indicated proteins in MCF-7 cells treated with different concentrations of Paclitaxel and SB-T-101141 for 24 hours, respectively. **(F)** Immunoblots of proteins in MCF-7 cells as (E). **(G)** Immunoblots of MCF-7 and MDA-MB-453 cells with the indicated antibodies. Cells were treated with Paclitaxel (MCF-7, 3 μM; MDA-MB-453, 8 μM) and SB-T-101141 (MCF-7, 3 μM; MDA-MB-453, 8 μM) for different time points.

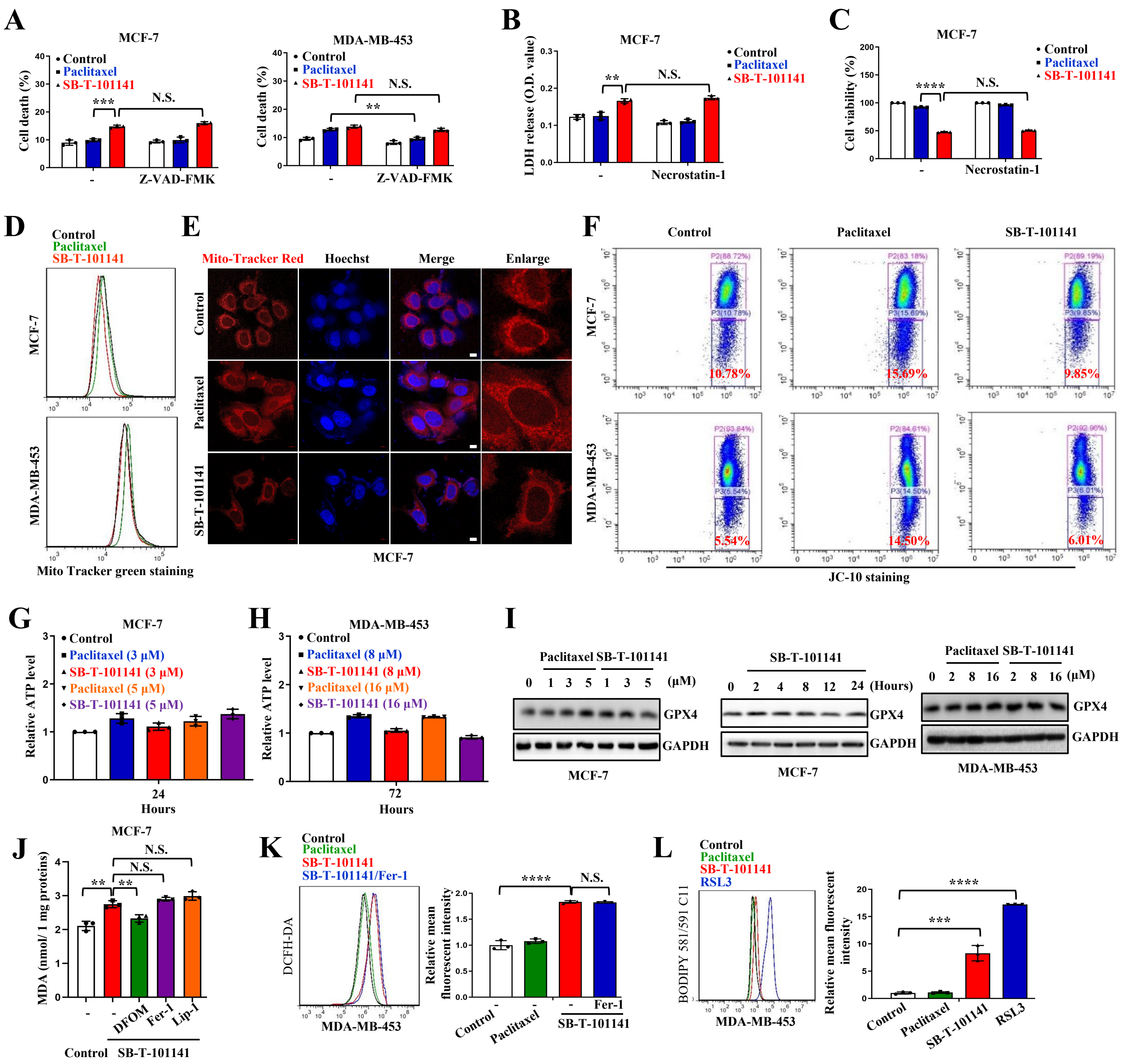

Figure S3. SB-T-101141 induces a novel ferroptosis in breast cancer cells

(A) PI staining of MCF-7 and MDA-MB-453 cells pretreated with Z-VAD-FMK (20 μM), followed by Paclitaxel (MCF-7, 3 μM; MDA-MB-453, 8 μM) or SB-T-101141 (MCF-7, 3 μM; MDA-MB-453, 8 μM) for 24 hours. The results were plotted and analyzed using the student *t*-test (mean+/-SD, n = 3) (*P < 0.05, **P < 0.01, ***P < 0.001, and ****P < 0.0001). **(B, C)** LDH detection (B) and cell viability assay (C) of MCF-7 cells pretreated with Necrostatin-1 and then with Paclitaxel (3 μM) and SB-T-101141 (3 μM) for 24 hours. **(D, E)** Detection of mitochondria of MCF-7 and MDA-MB-453 cells using Mito Tracker green (D) or Mito-Tracker Red CMXRos (E), respectively. Cell were treated with Paclitaxel (MCF-7, 3 μM; MDA-MB-453, 8 μM) and SB-T-101141 (MCF-7, 3 μM; MDA-MB-453, 8 μM) for 24 hours (MCF-7) or 48 hours (MDA-MB-453). Bar indicates 10 μm.

(F) Detections of mitochondria membrane potential of MCF-7 and MDA-MB-453 cells with JC-10 staining, respectively. Cells were treated with Paclitaxel (MCF-7, 3 μM; MDA-MB-453, 8 μM) and SB-T-101141 (MCF-7, 3 μM; MDA-MB-453, 8 μM) for 12 hours (MCF-7) or 48 hours (MDA-MB-453). **(G, H)** ATP level detection of MCF-7 (G) and MDA-MB-453 (H) cells treated with the indicated concentrations of Paclitaxel and SB-T-101141 at different time. **(I)** Immunoblots of MCF-7 and MDA-MB-453 cells treated with different concentrations of Paclitaxel and SB-T-101141 for 24 hours (MCF-7) or 48 hours (MDA-MB-453) . **(J)** MDA detection of MCF-7 cells pretreated with DFOM (50 μM), Fer-1 (30 μM) or Lip-1 (5 μM) for 1 hour and then with SB-T-101141 (3 μM) for 24 hours. **(K, L)** ROS (K) and lipid ROS (L) detections of MDA-MB-453 using a DCFH-DA probe and BODIPY 581/591 C11 probe, respectively. Cells were either pretreated with Fer-1 (30 μM) for 1 hour, or followed with Paclitaxel (8 μM), SB-T-101141 (8 μM) or RSL3 (1 μM) for 12 hours. All results statistically shown above were repeated and analyzed using student *t*-test (mean+/-SD, n = 3) (*P < 0.05, **P < 0.01, ***P < 0.001, and ****P < 0.0001).

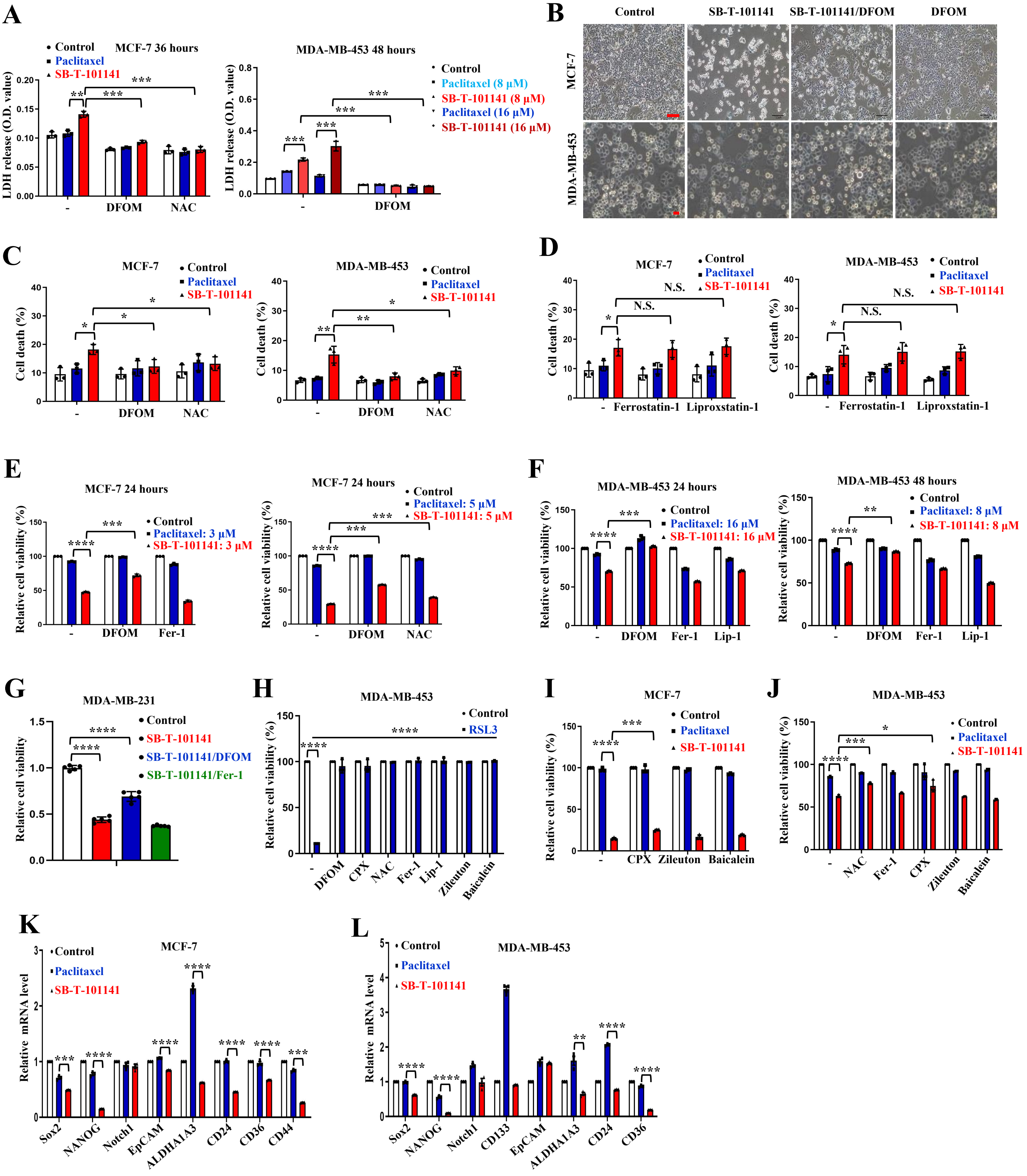

Figure S4. SB-T-101141 induces iron-dependent cell death in breast cancer cells

(A) LDH detections of MCF-7 and MDA-MB-453 cells pretreated with DFOM (100 μM) or NAC (5 mM) for 1 hour and then respectively treated with Paclitaxel (MCF-7, 3 μM; MDA-MB-453, 8 or 16 μM) and SB-T-101141 (MCF-7, 3 μM; MDA-MB-453, 8 or 16 μM) for indicated time points (MCF-7, 36 hours; MDA-MB-453, 48 hours). **(B)** Cell morphology of MCF-7 and MDA-MB-453 cells pretreated with DFOM (100 μM) for 1 hour, and then with Paclitaxel (MCF-7, 3 μM; MDA-MB-453, 8 μM) and SB-T-101141 (MCF-7, 3 μM; MDA-MB-453, 8 μM) for 24 hours, respectively, and visualized under a microscope. Bar indicates 100 μm.

(C) PI staining of MCF-7 and MDA-MB-453 cells pretreated with DFOM (100 μM) and NAC (5 mM) for 1 hour and with Paclitaxel (MCF-7, 3 μM; MDA-MB-453, 8 μM) and SB-T-101141 (MCF-7, 3 μM; MDA-MB-453, 8 μM) for 24 hours, respectively. **(D)** PI staining of MCF-7 and MDA-MB-453 cells pretreated with Fer-1 (30 μM) or Lip-1 (5 μM) and then with with Paclitaxel (MCF-7, 3 μM; MDA-MB-453, 8 μM) and SB-T-101141 (MCF-7, 3 μM; MDA-MB-453, 8 μM) for 24 hours, respectively, and analyzed as (D). **(E)** Cell viability analysis of MCF-7 cells pretreated with DFOM (100 μM), NAC (5 mM) or Fer-1 (30 μM) for 1 hour and then respectively treated with Paclitaxel (MCF-7, 3 or 5 μM) and SB-T-101141 (MCF-7, 3 or 5 μM) for 24 hours. **(F)** Cell viability analysis of MDA-MB-453 cells pretreated with DFOM (100 μM) , Fer-1 (30 μM) or Lip-1 (5 μM) for 1 hour and then respectively treated with Paclitaxel (MDA-MB-453, 8 or 16 μM) and SB-T-101141 (MDA-MB-453, 8 or 16 μM) for 24 or 48 hours. **(G)** Cell viability analysis of MDA-MB-231 cells pretreated with DFOM or Fer-1 for 1 hour and then treated with SB-T-101141 for 48 hours. **(H)** Cell viability analysis of MDA-MB-453 cells pretreated with DFOM, CPX, NAC, Fer-1, Lip-1, Zileuton or Baicalein for 1 hour and then with RSL3 (1 μM) for 48 hours. **(I, J)** Cell viability of MCF-7 (I) and MDA-MB-453 (J) cells pretreated with NAC, Fer-1, CPX, Zileuton or Baicalein for 1 hour and then respectively disposed with Paclitaxel (MCF-7, 3 μM; MDA-MB-453, 8 μM) and SB-T-101141 (MCF-7, 3 μM; MDA-MB-453, 8 μM) for 24 hours (MCF-7) or 48 hours (MDA-MB-453). **(K, L)** qRT-PCR analyses of the indicated genes of MCF-7 (K) and MDA-MB-453 (L) cells incubated with Paclitaxel (MCF-7, 3 μM; MDA-MB-453, 8 μM) or SB-T-101141 (MCF-7, 3 μM; MDA-MB-453, 8 μM) for 24 hours. All results statistically shown above were repeated and analyzed using student *t*-test (mean+/-SD, n = 3) (*P < 0.05, **P < 0.01, ***P < 0.001, and ****P < 0.0001).

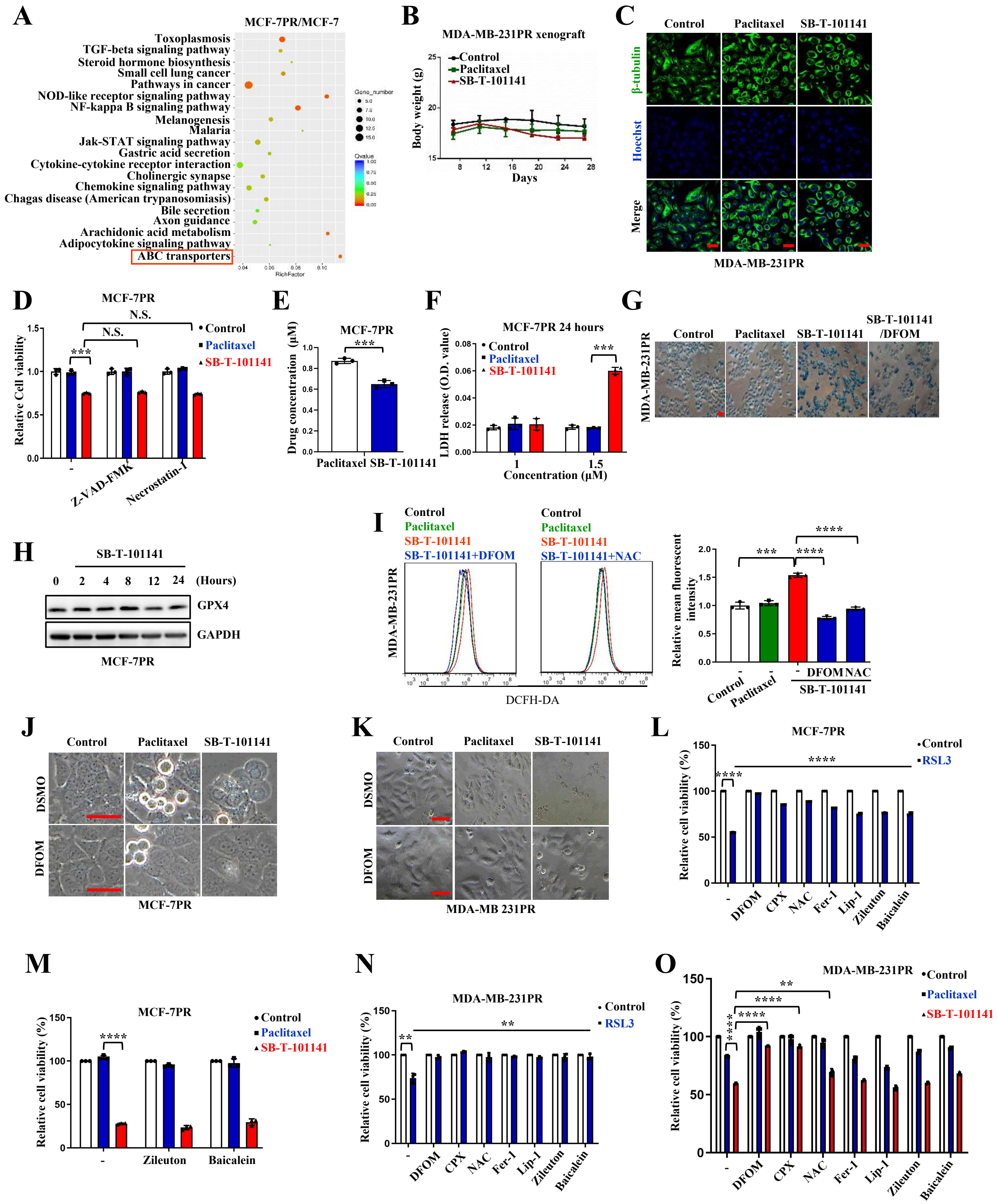

Figure S5. SB-T-101141 induces a novel ferroptosis in Paclitaxel-resistant breast cancer cells

(A) RNA sequencing of MCF-7 and Paclitaxel-resistant MCF-7 (MCF-7PR) cells. Red rectangle indicates ABC transporter pathway. **(B)** Xenograft tumor formation of MDA-MB-231PR cells. Mice bearing tumors were administrated with Paclitaxel or SB-T-101141. The body weight of mice was monitored (mean+/-SD, n = 4) (*P < 0.05, **P < 0.01, ***P < 0.001, and ****P < 0.0001, *ANOVA* test). **(C)** Immunofluorescence of MDA-MB-231PR treated with Paclitaxel (110 nM) or SB-T-101141 (110 nM) for the indicated time point. Bar indicates 20 μm.

(D) Cell viability of MCF-7PR cells pretreated with Z-VAD-FMK or Necrostatin-1 for 1 hour and then with Paclitaxel (1.5 μM) and SB-T-101141 (1.5 μM) for 24 hours. **(E)** Mass spectrometry of the intracellular drug concentration of the indicated drugs in MCF-7PR cells after incubation with Paclitaxel (1.5 μM) and SB-T-101141 (1.5 μM) for 4 hours. **(F)** LDH detection of MCF-7PR cells treated with varied concentrations of Paclitaxel or SB-T-101141 for 24 hours. **(G)** Prussian blue staining of MDA-MB-231PR cells pretreated with DFOM for 1 hour and then with Paclitaxel (170 nM) or SB-T-101141 (170 nM) for 24 hours. Bar indicates 100 μm. **(H)** Immunoblots of MCF-7PR cells treated with SB-T-101141 (1.5 μM) for different time points. **(I)** ROS detection of MDA-MB-231PR cells with the DCFH-DA probe (left panel). Cells were treated with DFOM or NAC for 1 hour, and with Paclitaxel (170 nM) or SB-T-101141 (170 nM) for 24 hours. **(J, K)** Cell morphology of MCF-7PR (J) and MDA-MB-231PR (K) under microscope. Cells were pretreated with DFOM for 1 hour and then with Paclitaxel (MCF-7PR, 1.5 μM; MDA-MB-231PR, 170 nM) and SB-T-101141 Paclitaxel (MCF-7PR, 1.5 μM; MDA-MB-231PR, 170 nM) for 24 hours (MCF-7PR) or 48 hours (MDA-MB-231PR). Bar indicates 100 μm. **(L)** Cell viability of MCF-7PR cells pretreated with DFOM, CPX, NAC, Fer-1, Lip-1, Zileuton or Baicalein for 1 hour and then with RSL3 (1 μM) for 24 hours. **(M)** Cell viability of MCF-7PR cells individually pretreated with Zileuton or Baicalein for 1 hour and then respectively with Paclitaxel (1.5 μM) and SB-T-101141 (1.5 μM) for 24 hours. **(N)** Cell viability of MDA-MB-231PR cells pretreated with DFOM, CPX, NAC, Fer-1, Lip-1, Zileuton or Baicalein for 1 hour and then with RSL3 (1 μM) for 48 hours. **(O)** Cell viability of MDA-MB-231PR cells individually pretreated as (M) and then with either Paclitaxel (250 nM) or SB-T-101141 (250 nM) for 48 hours. All results statistically shown above were repeated and analyzed using student *t*-test (mean+/-SD, n = 3) (*P < 0.05, **P < 0.01, ***P < 0.001, and ****P < 0.0001).

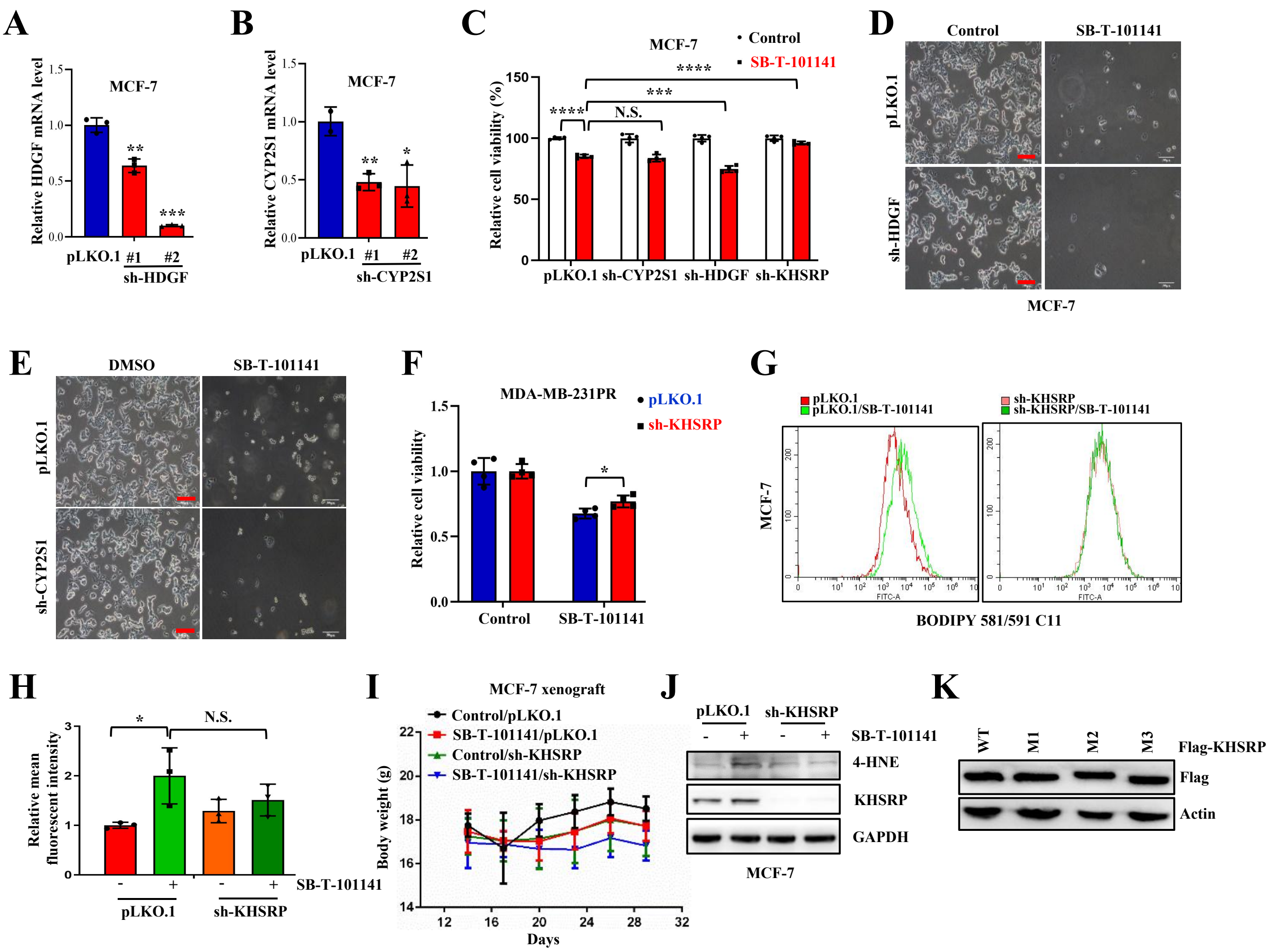

Figure S6. SB-T-101141 represses the growth of breast cancer cells by KHSRP

(A-E) qRT-PCR (A, B), cell viability (C) and microscope (D, E) analyses or MCF-7 cells. Cells were respectively infected with lentiviruses encoding sh-HDGF, sh-CYP2S1 or sh-KHSRP and then selected with puromycin (1 μg/ml). The cells were disposed with SB-T-101141 (3 μM) for 24 hours. Results were analyzed using student *t* test (mean+/-SD, n = 3) (*P < 0.05, **P < 0.01, ***P < 0.001, and ****P < 0.0001). **(F)** Cell viability analysis of MDA-MB-231PR cells infected with lentiviruses encoding sh-KHSRP, and then selected with puromycin (1 μg/ml). The selected cells were treated with SB-T-101141 (250 nM) for 48 hours. Results were analyzed using student *t* test (mean+/-SD, n = 3) (*P < 0.05, **P < 0.01, ***P < 0.001, and ****P < 0.0001). **(G, H)** Lipid ROS detection of *KHSRP* knock-down and its counterpart MCF-7 cells using the BODIPY 581/591 C11 probe (G). Cells were incubated with SB-T-101141 (3 μM) for 16 hours. Results were analyzed using student *t* test (H) (mean+/-SD, n = 3) (*P < 0.05, **P < 0.01, ***P < 0.001, and ****P < 0.0001). **(I)** Xenograft tumor formation of MCF-7 cells with *KHSRP* knock-down. Mice bearing tumors with sh-KHSRP or control were respectively administrated with SB-T-101141. The body weight of mouse was monitored (mean+/-SD, n = 4) (*P < 0.05, **P < 0.01, ***P < 0.001, and ****P < 0.0001, *ANOVA* test). **(J)** Immunoblots the indicated proteins in the *KHSRP* knock-down and parental MCF-7 cells. **(K)** Immunoblots of proteins in HEK293T cells transfected with the plasmid wild-type or mutant KHSRP, respectively.

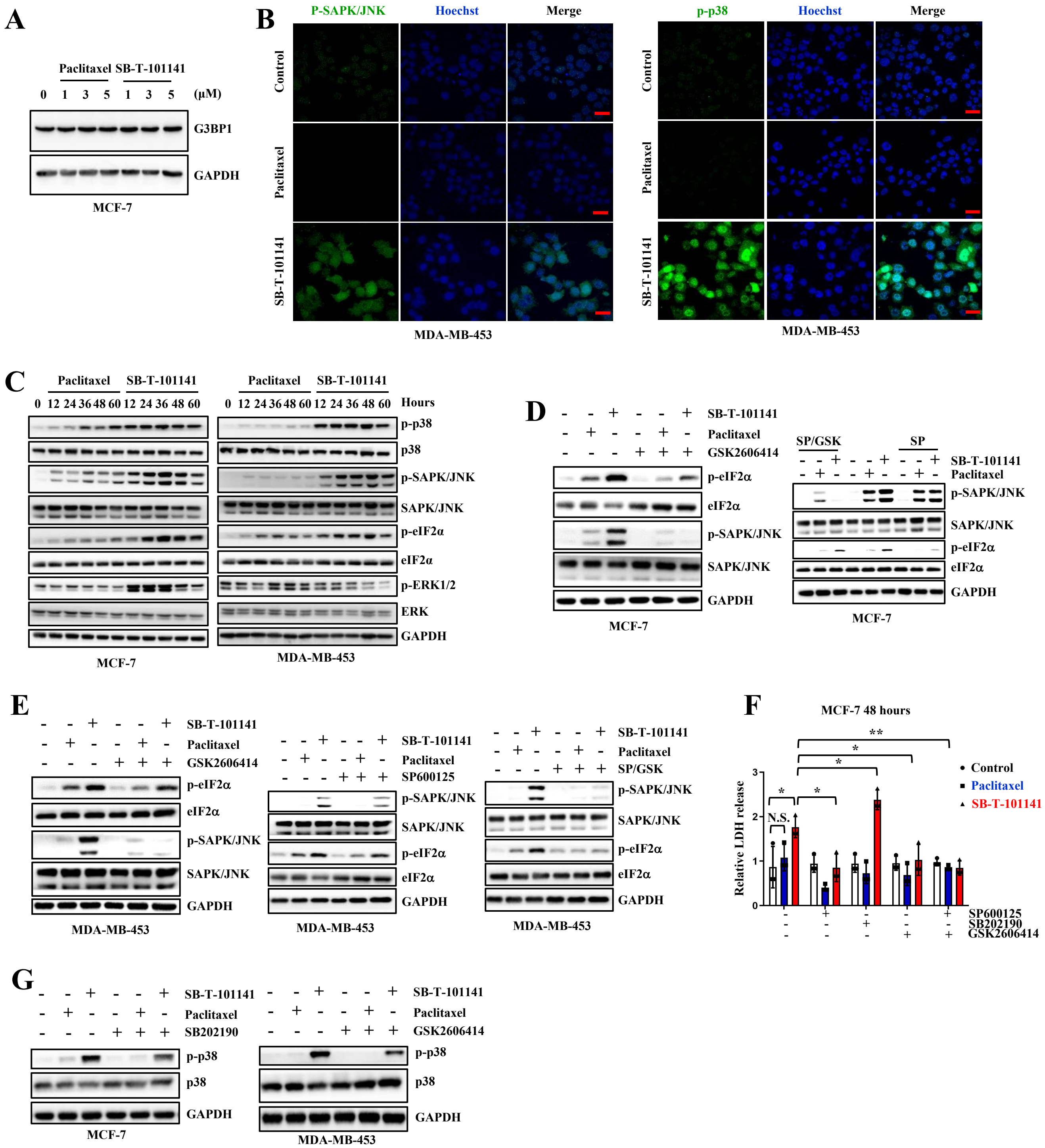

Figure S7. SB-T-101141 induces cell death by activating JNK and PERK pathways

(A) Immunoblots of the indicated proteins in MCF-7 cells treated with different concentrations of Paclitaxel and SB-T-101141 for 4 hours. **(B)** Immunofluorescence of MDA-MB-453 cells individually treated with with Paclitaxel (8 μM) and SB-T-101141 (8 μM) for 24 hours. Bar indicates 20 μm. **(C)** Immunoblots of the indicated proteins in MCF-7 and MDA-MB-453 cells. Cells were treated with Paclitaxel (MCF-7, 3 μM; MDA-MB-453, 8 μM) or SB-T-101141 (MCF-7, 3 μM; MDA-MB-453, 8 μM) at different time points. **(D, E)** Immunoblots of MCF-7 cells (D) or MDA-MB-453 cells (E) pretreated with SP600125, SB202190 or GSK2606414 for 1 hour, followed by Paclitaxel (MCF-7, 3 μM; MDA-MB-453, 8 μM) and SB-T-101141 (MCF-7, 3 μM; MDA-MB-453, 8 μM) for 24 hours.

(F) LDH detection of MCF-7 cells respectively pretreated with SP600125, SB202190 or GSK2606414 for 1 hour and then disposed with Paclitaxel (5 μM) and SB-T-101141 (5 μM) for 48 hours. The results were analyzed using student *t* test (mean+/-SD, n = 3) (*P < 0.05, **P < 0.01, ***P < 0.001, and ****P < 0.0001). **(G)** Immunoblots of MCF-7 and MDA-MB-453 cells pretreated with SB202190 for 1 hour and then disposed with Paclitaxel (MCF-7, 3 μM; MDA-MB-453, 8 μM) and SB-T-101141 (MCF-7, 3 μM; MDA-MB-453, 8 μM) for indicated time points.

Supplemental Table S1-2

Table S1 The target sequences of shRNA and siRNA

Gene	No.	Target Sequence 5' to 3'
KHSRP	1	GACTTCAATGACAGAAGAGTA
	2	CCCGAGAAGATTGCTCATATA
HDGF	1	CTTCCCTTACGAGGAATCCAA
	2	GAACGAGAAAGGAGCGTTGAA
CYP2S1	1	GAAGTTTACCATGCTTGCTCT
	2	CCTGATGAAATACCCTCATGT

Table S2 The sequences of primers used for Quantitative real-time PCR

Gene	Forward, 5' to 3'	Reverse, 5' to 3'
Actin	CATGTACGTTGCTATCCAGGC	CTCCTTAATGTCACGCACGAT
CAT	TGGAGCTGGTAACCCAGTAGG	CCTTTGCCTTGGAGTATTTGGTA
CISD1	GATCGCAGCAGTTACCATTGC	GCATGTACTATCTTGGGGTTGTC
GAPDH	AATGAAGGGGTCATTGATGG	AAGGTGAAGGTCGGAGTCAA
Sox2	TACAGCATGTCCTACTCGCAG	GAGGAAGAGGTAACCACAGGG
Nanog	TTTGTGGGCCTGAAGAAAACT	AGGGCTGTCCTGAATAAGCAG
Notch1	GAGGCGTGGCAGACTATGC	CTTGTACTCCGTCAGCGTGA
CD133	TTCTTGACCGACTGAGACCCA	TCATGTTCTCCAACGCCTCTT
EpCAM	ATAACCTGCTCTGAGCGAGTG	TGCAGTCCGCAAACTTTTACTA
ALDHA1A3	TGAATGGCACGAATCCAAGAG	CACGTCGGGCTTATCTCCT
CD24	CTCCTACCCACGCAGATTTATTC	AGAGTGAGACCACGAAGAGAC
CD44	CTGCCGCTTTGCAGGTGTA	CATTGTGGGCAAGGTGCTATT
CD36	CTTTGGCTTAATGAGACTGGGAC	GCAACAAACATCACCACACCA
