# Supplementary material for: A novel taxane SB-T-101141 triggers a noncanonical ferroptosis to overcome Paclitaxel resistance of breast cancer via iron homeostasis-related KHSRP
Source: Cell Death Dis. 2025 May 19;16(1):403. doi: 10.1038/s41419-025-07710-0 (PMC12089390; doi:10.1038/s41419-025-07710-0)
Supplement: Supplementary file 2 — Original Data [file 41419_2025_7710_MOESM2_ESM.pdf]

Figure 6L

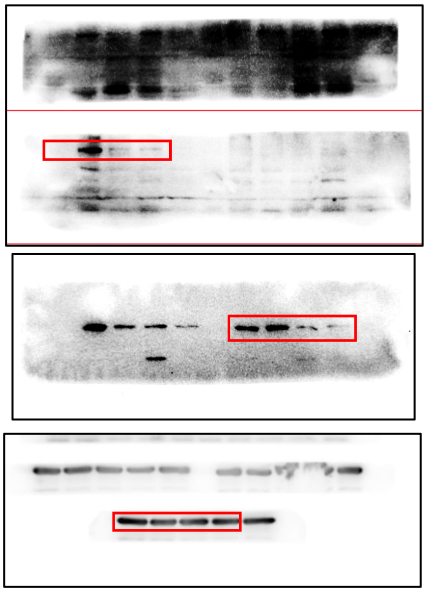

Figure 6M

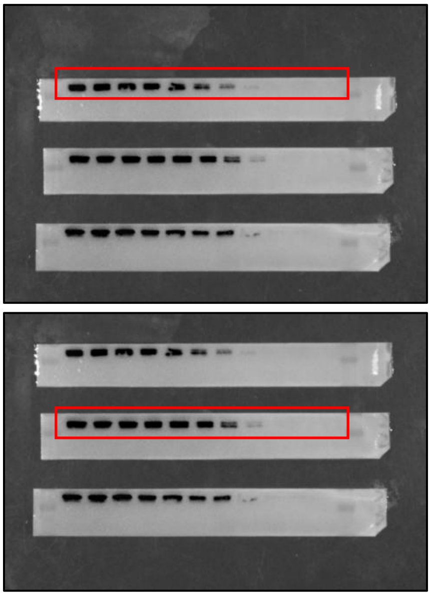

Figure 6N

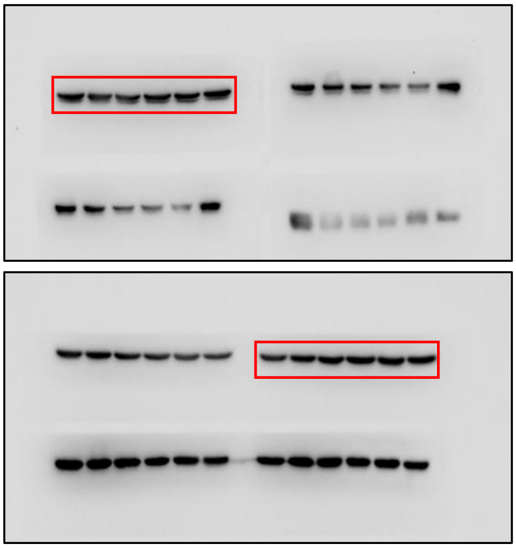

Figure 6N

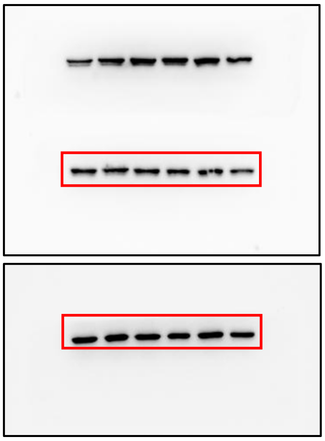

Figure 6P

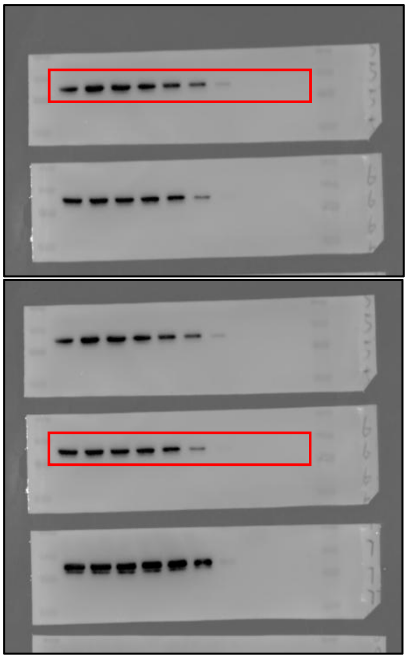

Figure 6P

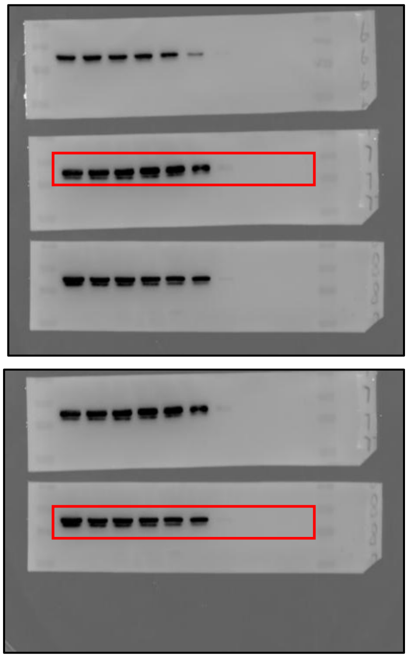

Figure 7K

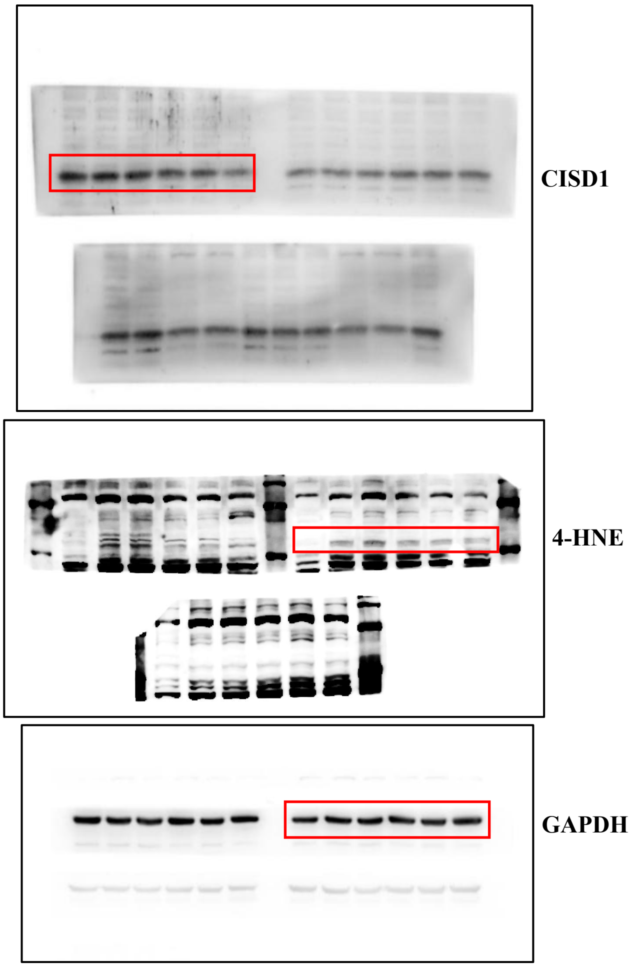

Figure 7L

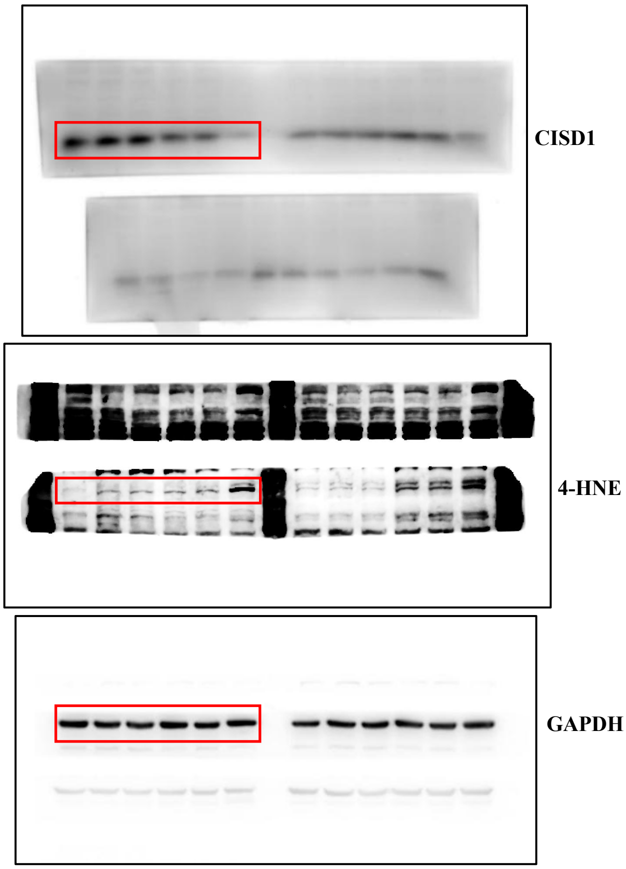

Figure 7N

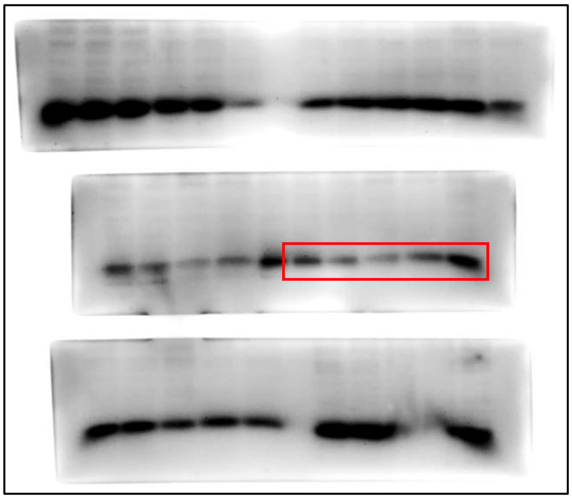

Figure 7N

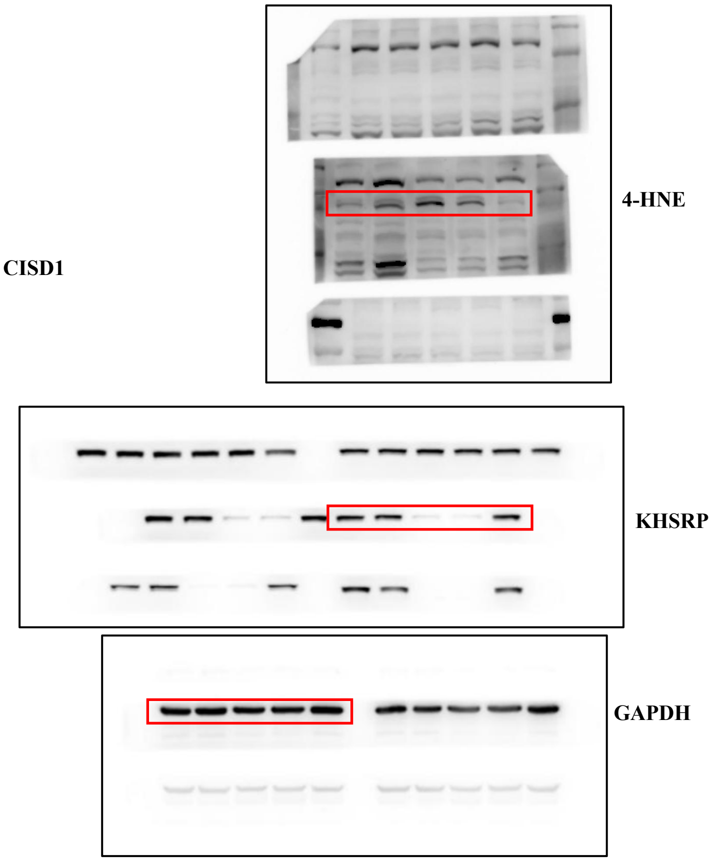

Figure 8C

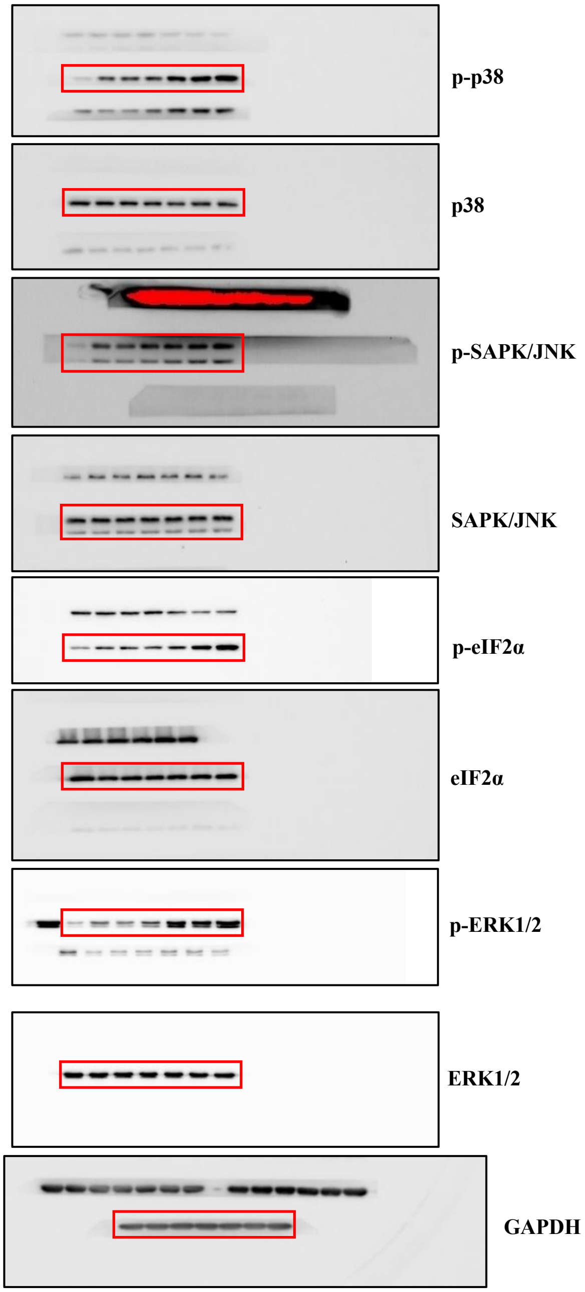

Figure 8C

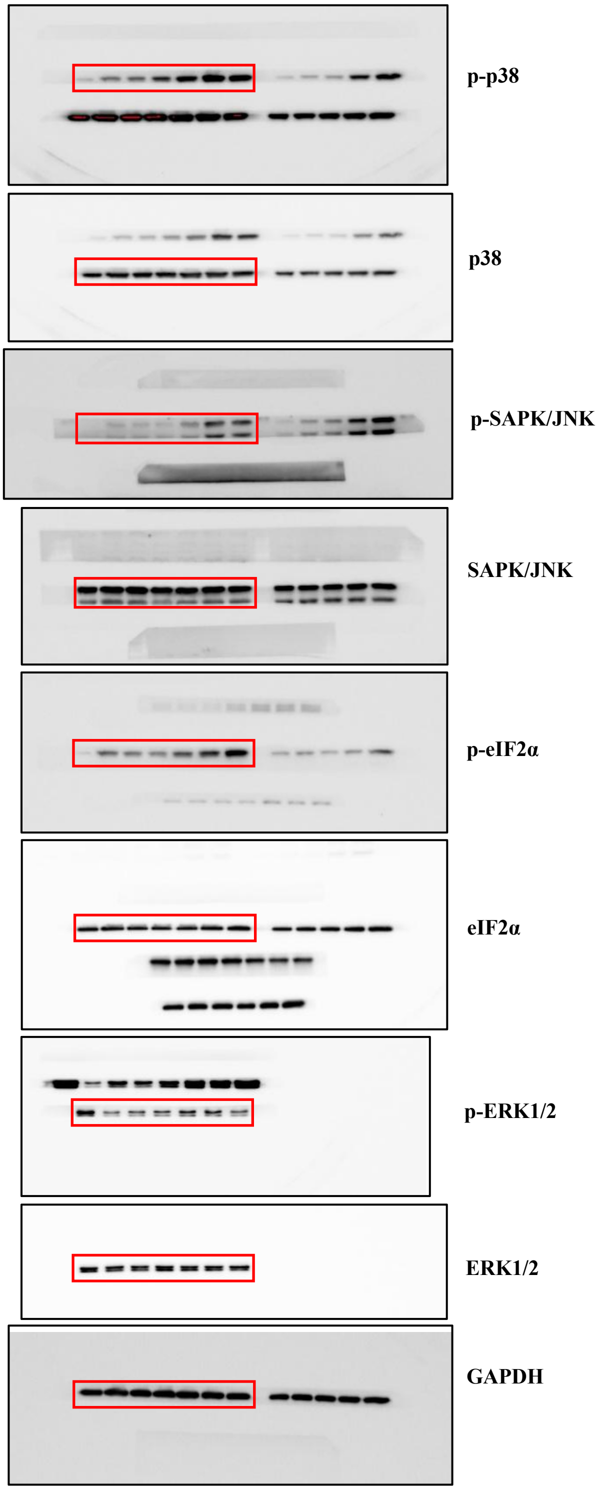

Figure 8D

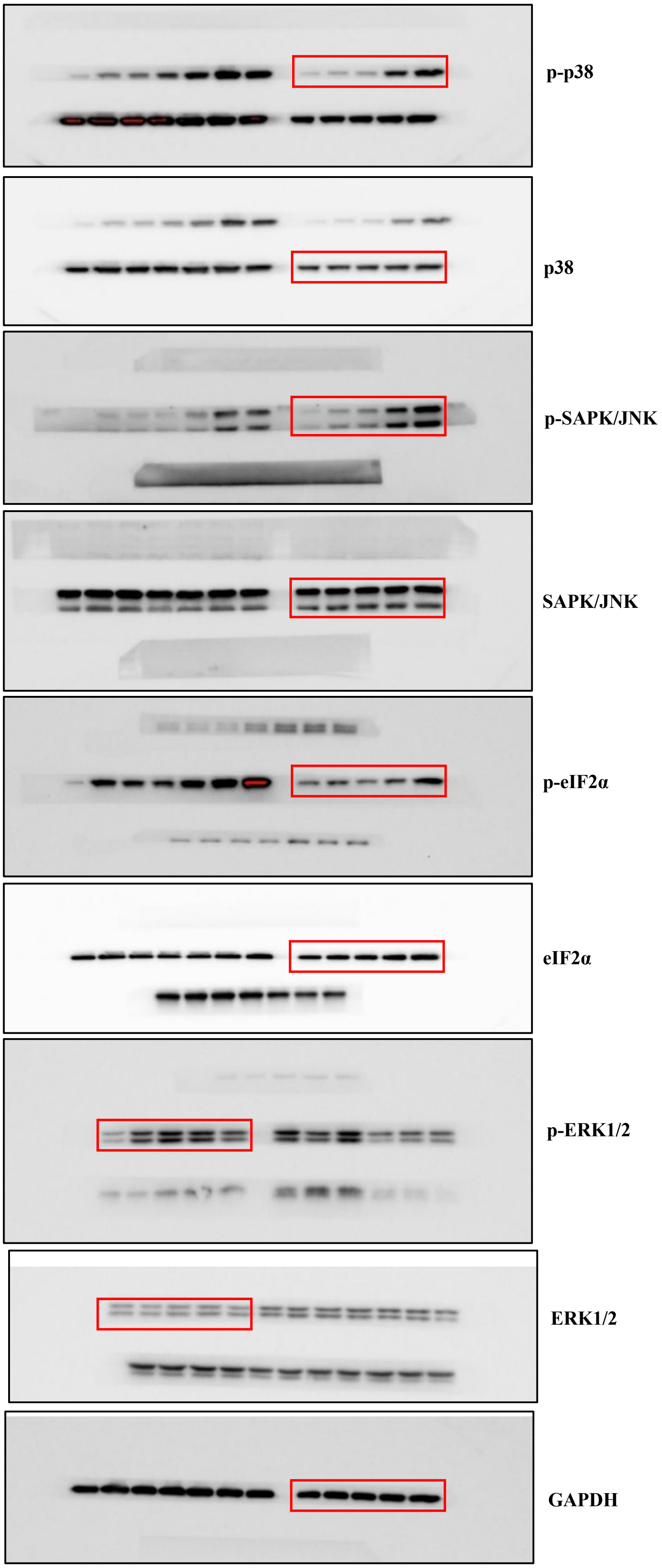

Figure 8D

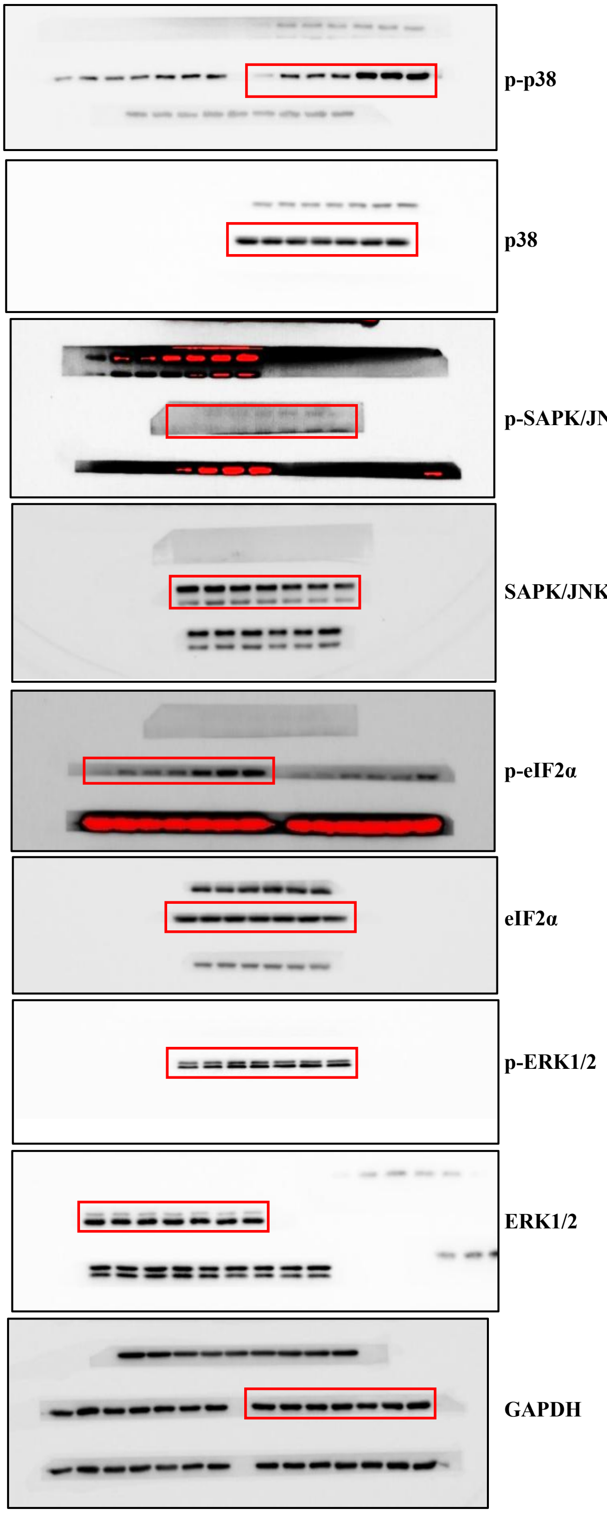

Figure 8E

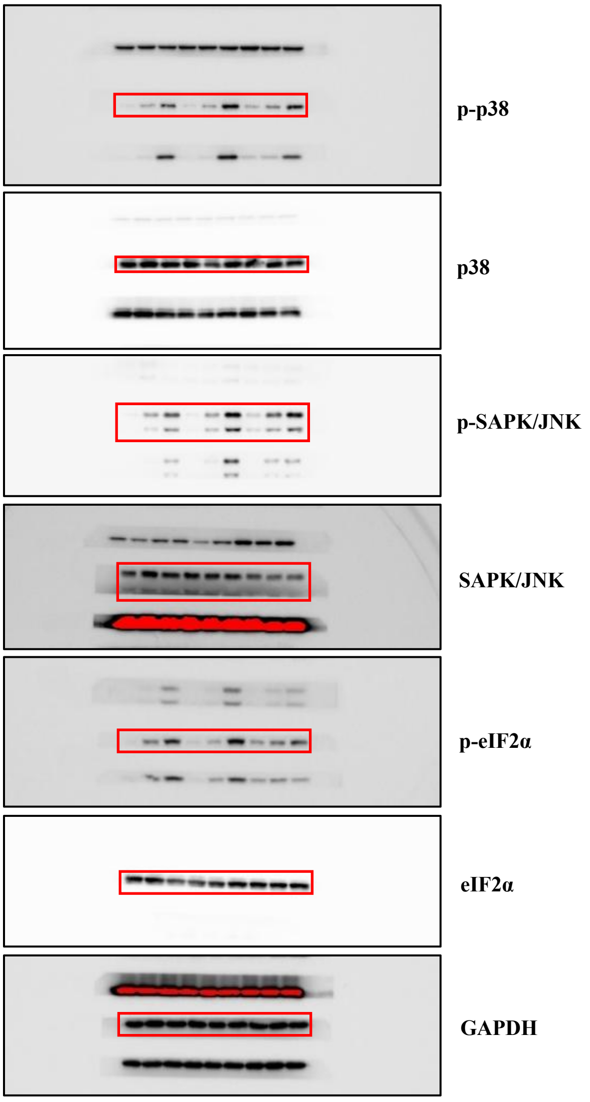

Figure 8E

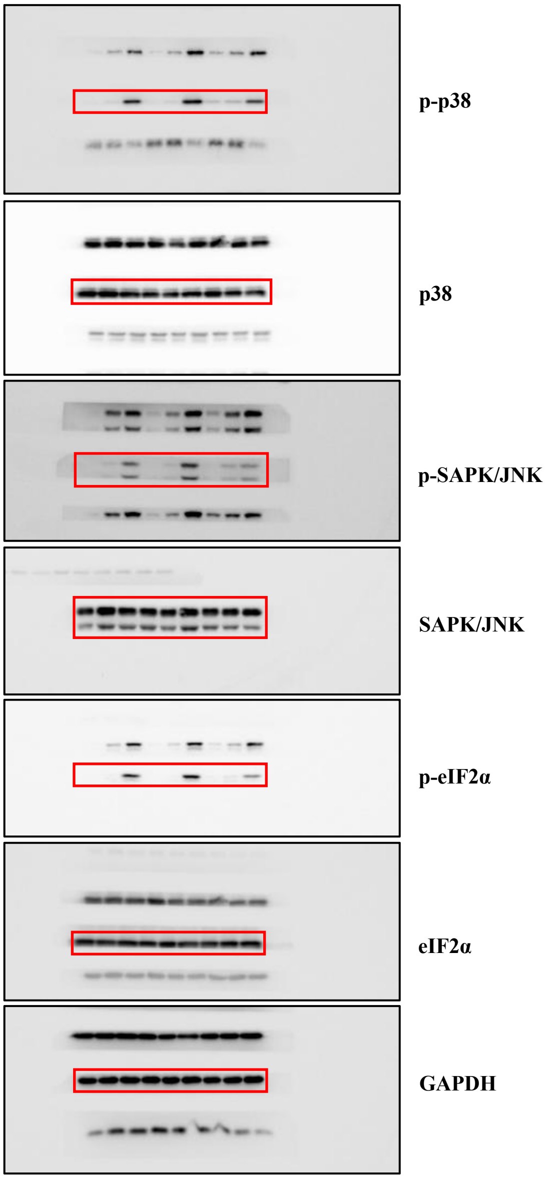

Figure 8F

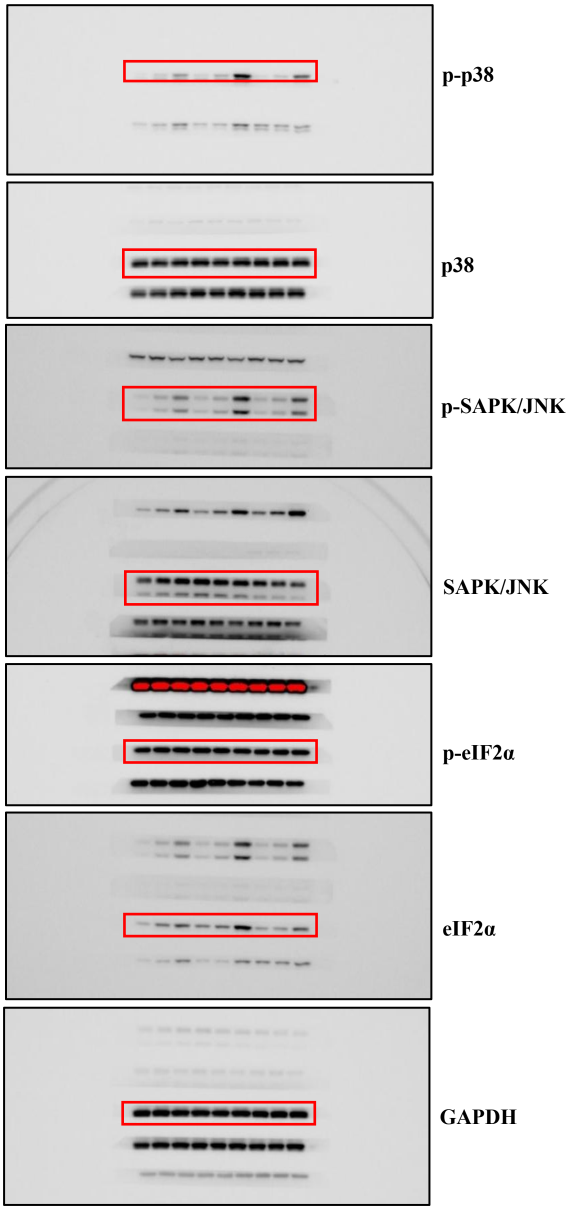

Figure 8F

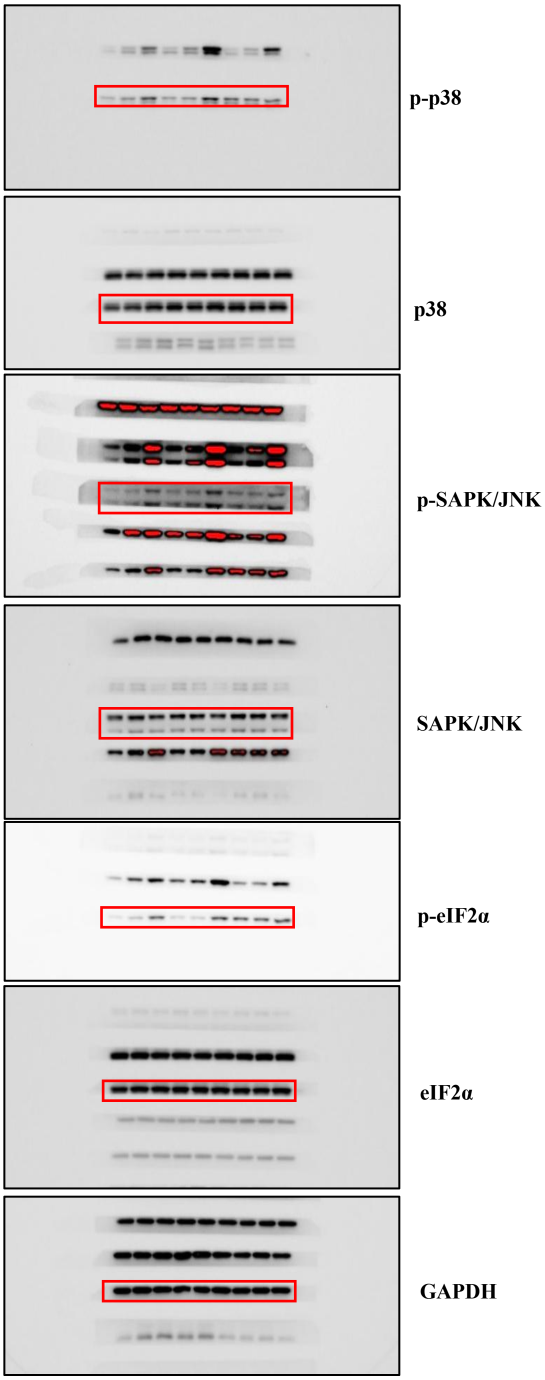

Figure 8G

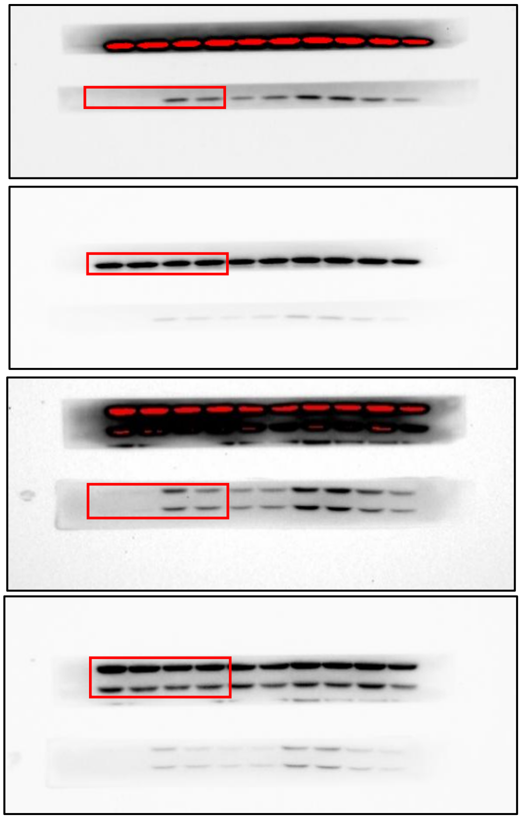

p-p38

p38

p-SAPK/JNK

SAPK/JNK

Figure 8G

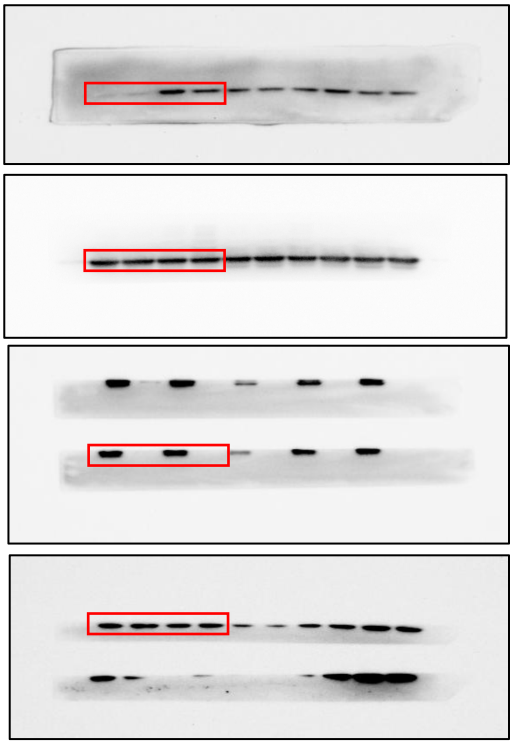

p-eIF2α

eIF2α

KHSRP

GAPDH

Figure S1A

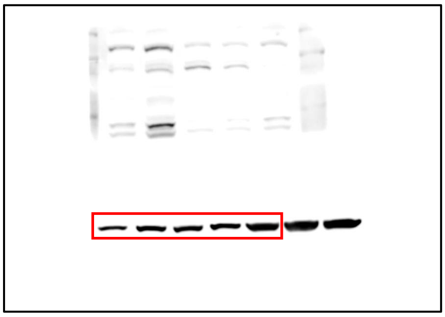

β-tubulin

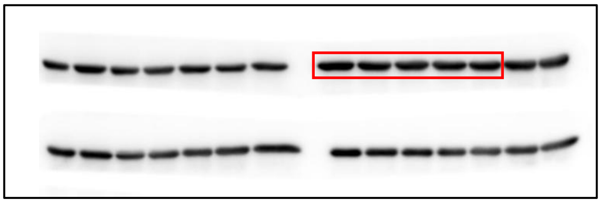

GAPDH

Figure S2E

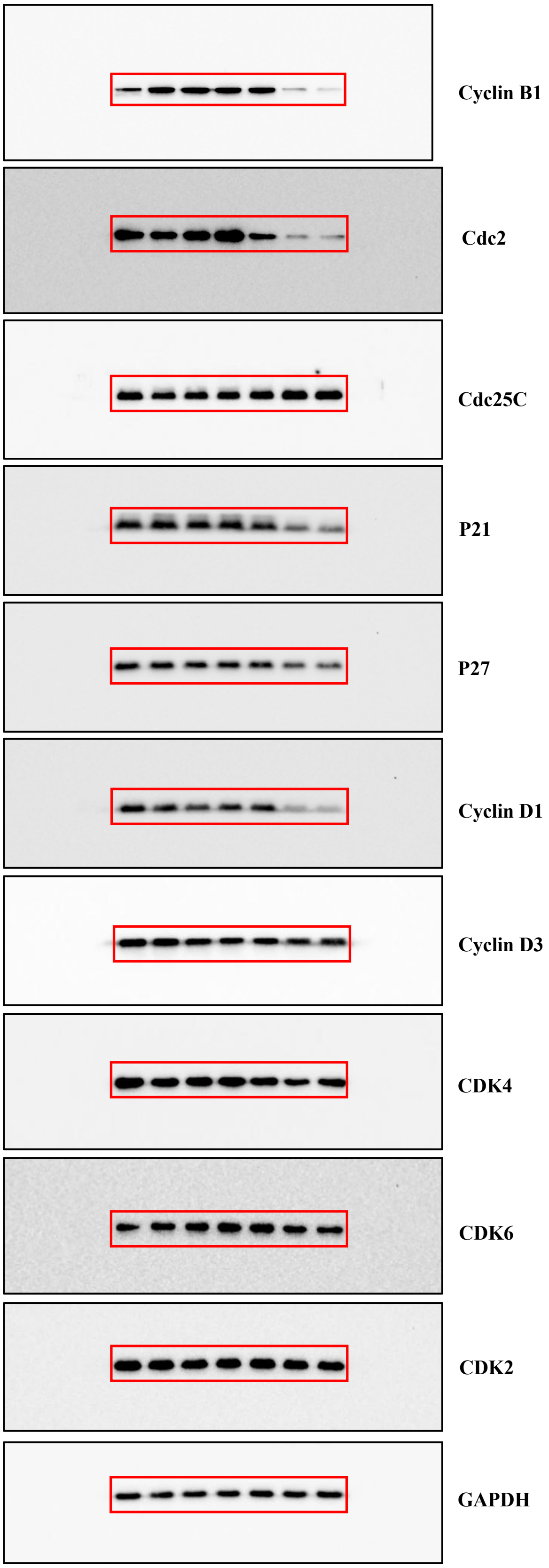

Figure S2F

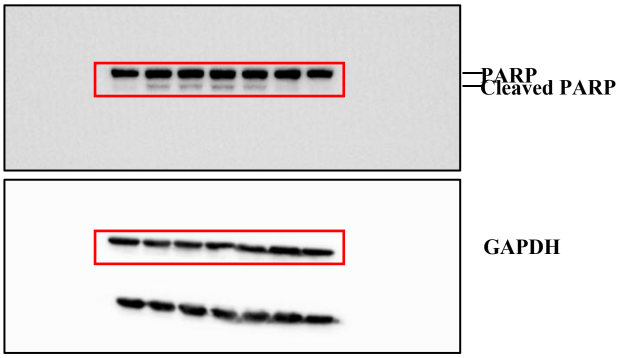

Figure S2G

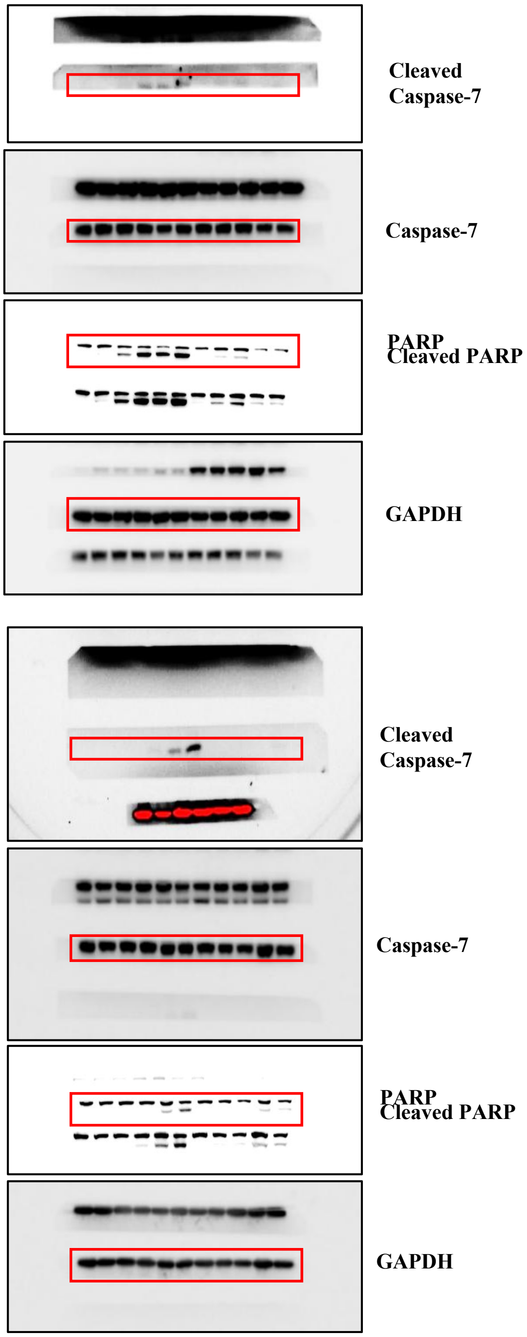

Figure S3I

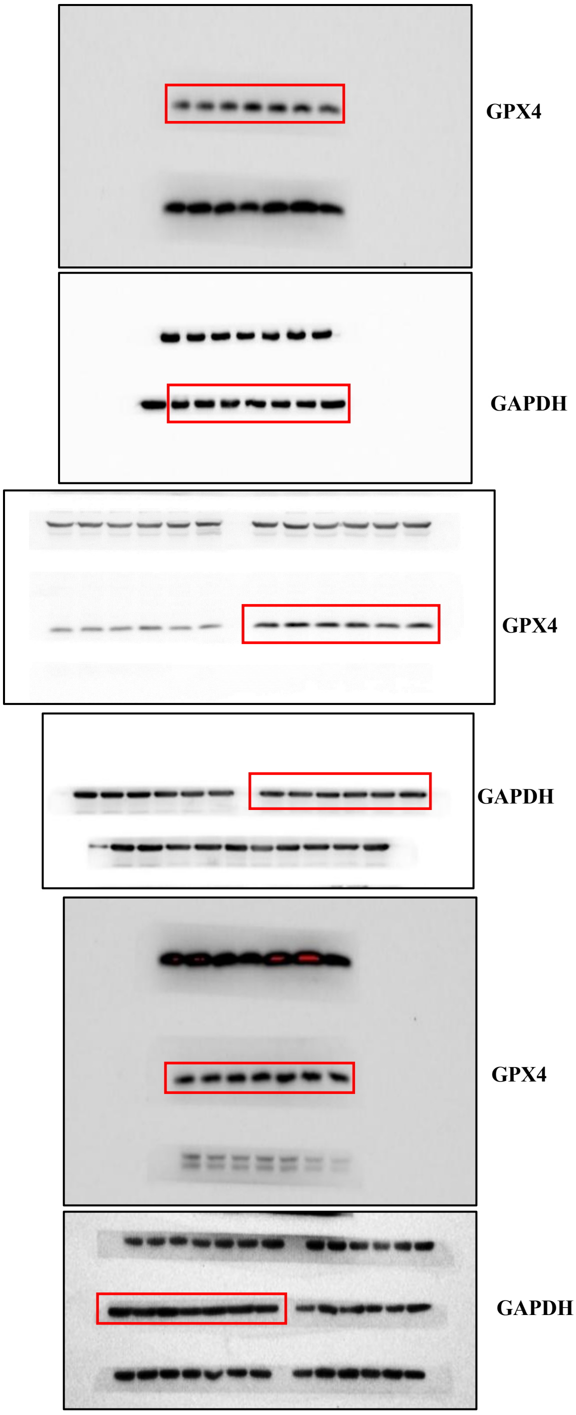

GPX4

GAPDH

GPX4

GAPDH

GPX4

GAPDH

Figure S5H

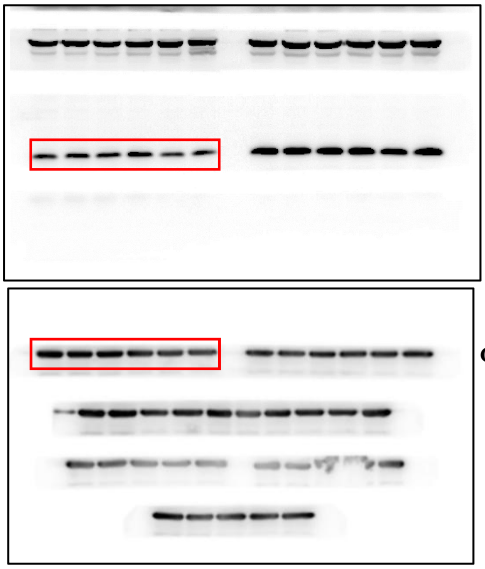

GPX4

GAPDH

Figure S6J

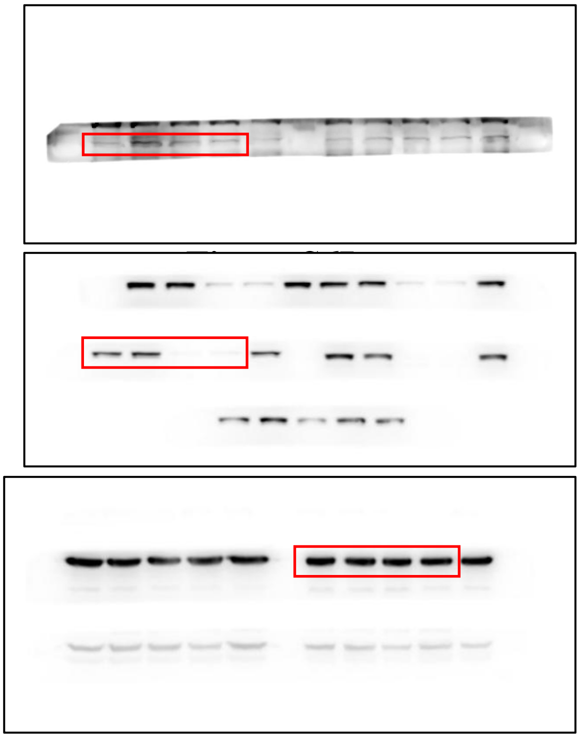

4-HNE

KHSR P

GAPDH

Figure S6K

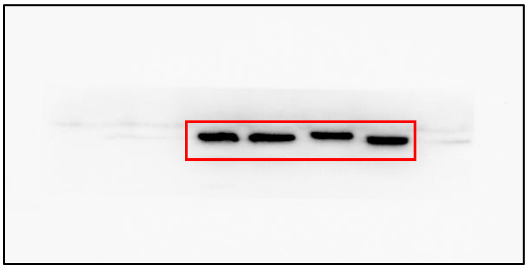

Flag

Figure S6K

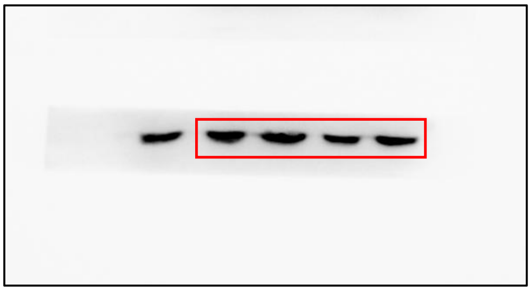

Actin

Figure S7A

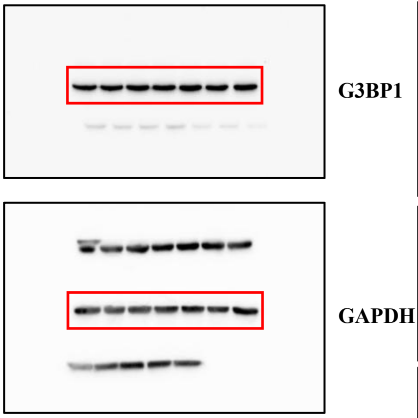

Figure S7C

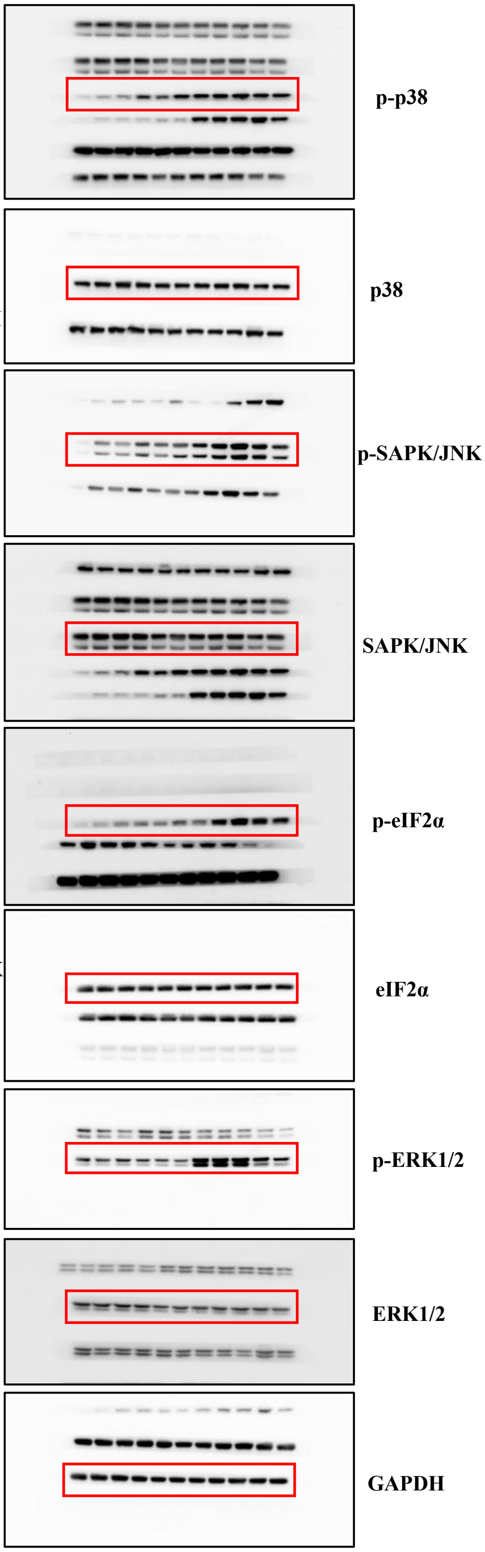

Figure S7C

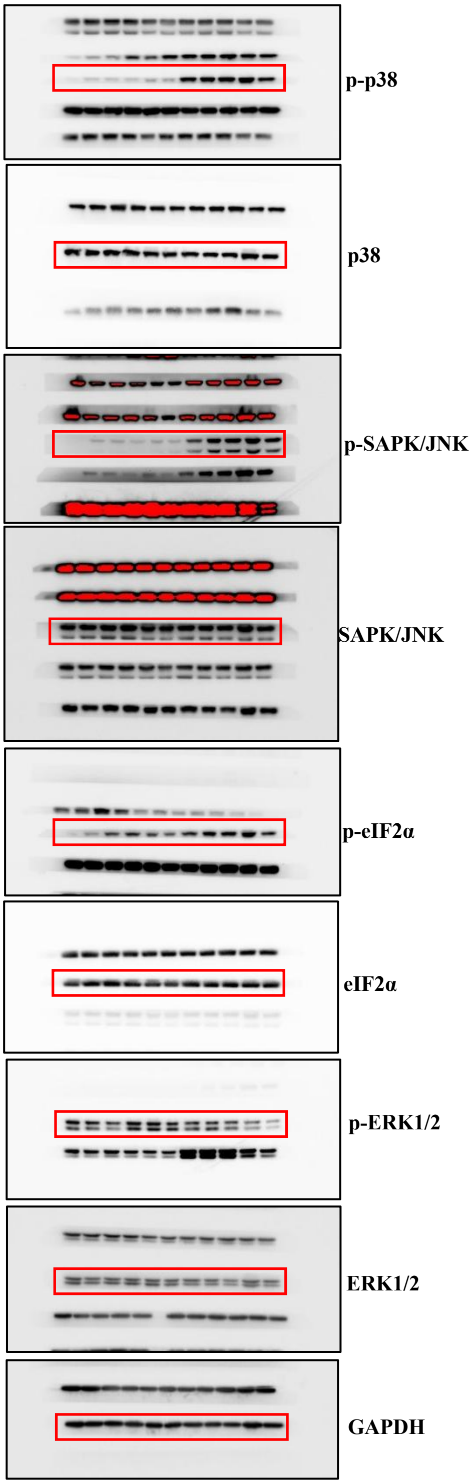

Figure S7D

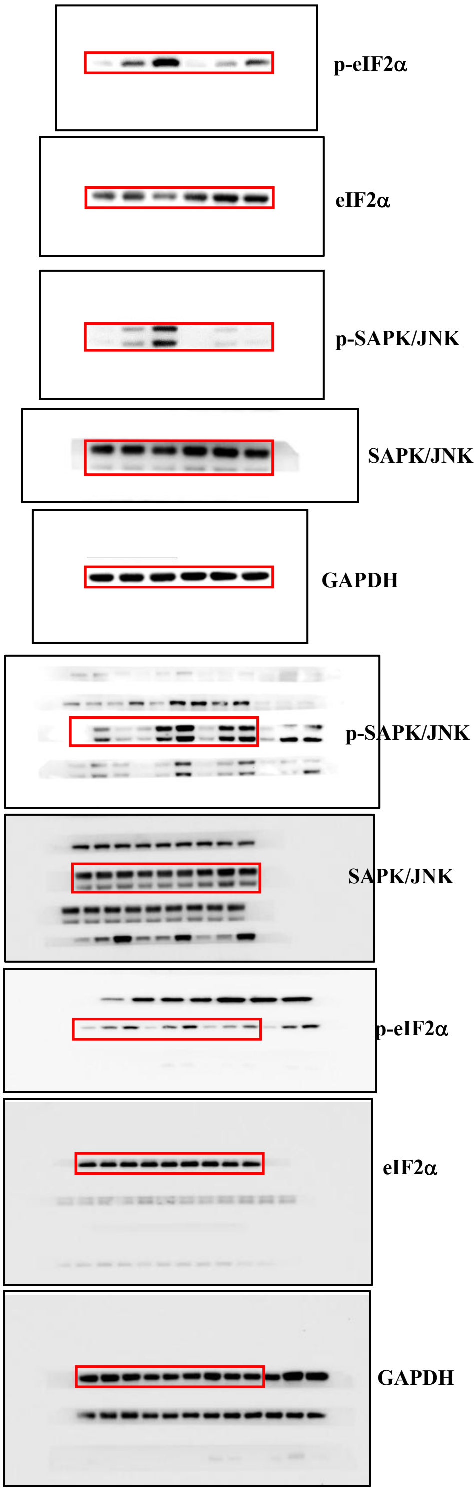

Figure S7E

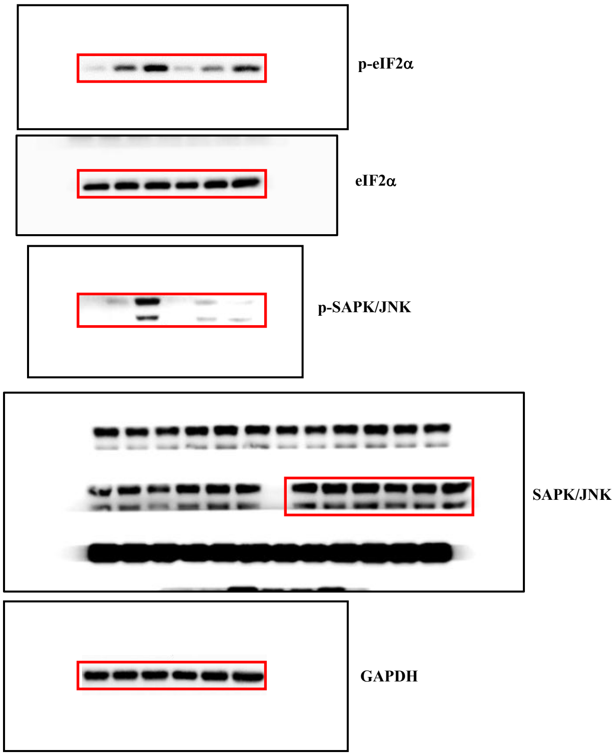

Figure S7E

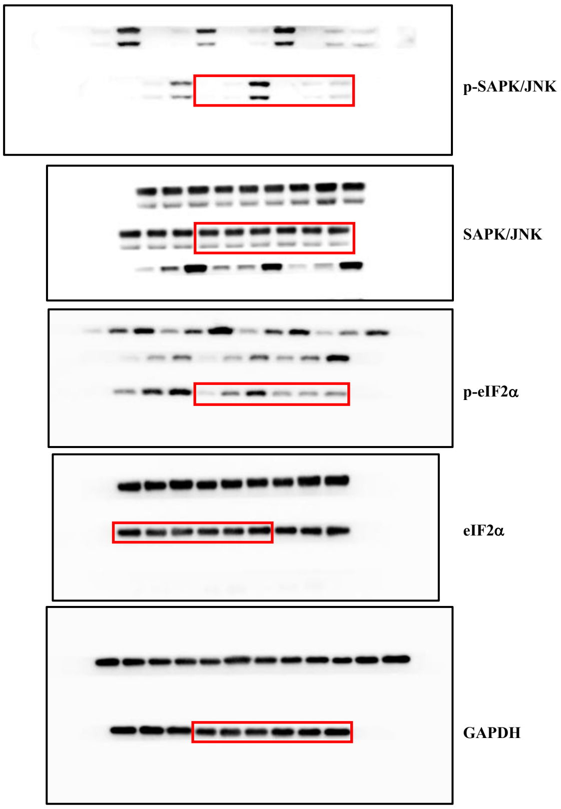

Figure S7E

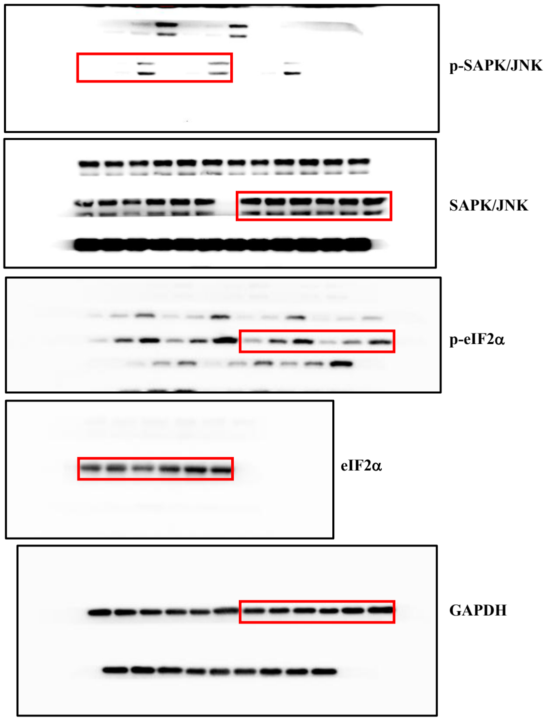

Figure S7G

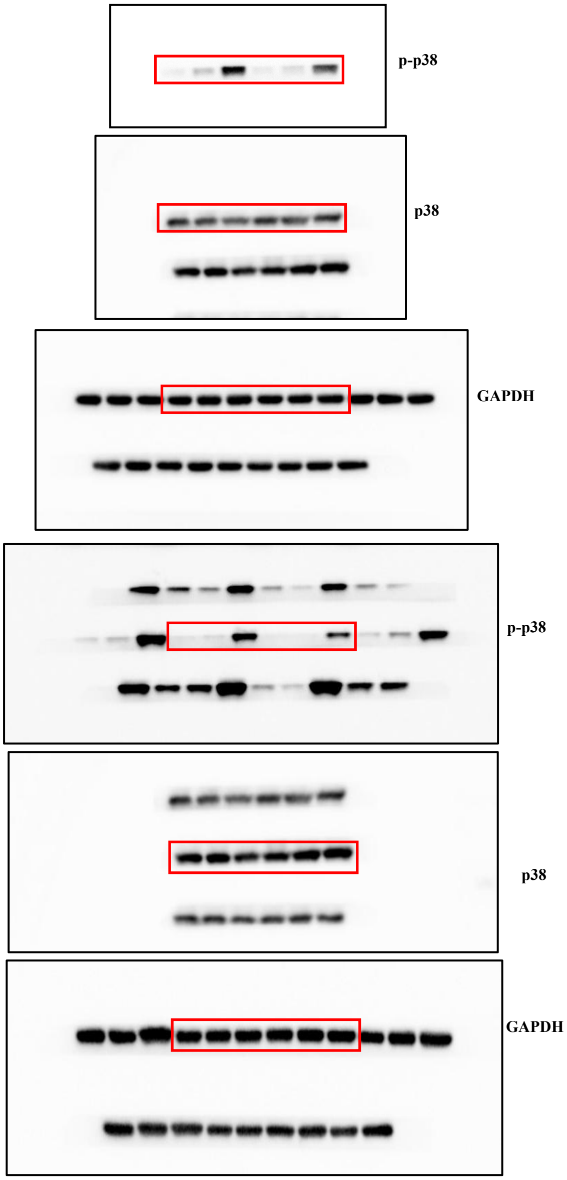

	Sample	Target	CT value
**Fig.6C**	PLKO.1	KHSRP	34.10999077
	PLKO.1	KHSRP	33.56572903
	PLKO.1	KHSRP	33.73112277
	sh-KHSRP-1	KHSRP	39.72787369
	sh-KHSRP-1	KHSRP	37.561542
	sh-KHSRP-1	KHSRP	38.55174922
	sh-KHSRP-2	KHSRP	36.23601333
	sh-KHSRP-2	KHSRP	37.38631059
	sh-KHSRP-2	KHSRP	35.73417056
	PLKO.1	GAPDH	23.22926478
	PLKO.1	GAPDH	23.37154448
	PLKO.1	GAPDH	23.1767095
	sh-KHSRP-1	GAPDH	23.69543114
	sh-KHSRP-1	GAPDH	23.57055201
	sh-KHSRP-1	GAPDH	23.70066003
	sh-KHSRP-2	GAPDH	22.39396767
	sh-KHSRP-2	GAPDH	22.56436816
	sh-KHSRP-2	GAPDH	22.25372345
**Fig.7I**	control	actin	16.757084
	control	actin	16.736863
	control	actin	16.757084
	101141	actin	16.566809
	101141	actin	16.5832
	101141	actin	16.566809
	101141-dfom	actin	17.315304
	101141-dfom	actin	17.16338
	101141-dfom	actin	17.16338
	control	CAT	25.924637
	control	CAT	25.911915
	control	CAT	25.911915
	101141	CAT	26.406229
	101141	CAT	26.406229
	101141	CAT	26.694305
	101141-dfom	CAT	26.52079
	101141-dfom	CAT	26.645076
	101141-dfom	CAT	26.52079
	control	CTSD1	23.721962
	control	CTSD1	23.643856
	control	CTSD1	23.721962
	101141	CTSD1	24.52572
	101141	CTSD1	24.52572
	101141	CTSD1	24.769894
	101141-dfom	CTSD1	24.772831
	101141-dfom	CTSD1	24.737457
	101141-dfom	CTSD1	24.737457
**Fig.7J**	control	actin	18.116047
	control	actin	18.370333
	control	actin	18.116047
	101141	actin	17.199383
	101141	actin	17.298158
	101141	actin	17.199383
	101141-dfom	actin	18.182726
	101141-dfom	actin	18.008158
	101141-dfom	actin	18.008158
	control	CAT	27.68715

	control	CAT	27.386312
	control	CAT	27.386312
	101141	CAT	26.09831
	101141	CAT	26.037544
	101141	CAT	26.037544
	101141-dfom	CAT	25.94544
	101141-dfom	CAT	25.9909
	101141-dfom	CAT	25.94544
	control	CTSD1	23.72994
	control	CTSD1	23.709686
	control	CTSD1	23.709686
	101141	CTSD1	23.487835
	101141	CTSD1	23.487835
	101141	CTSD1	23.68553
	101141-dfom	CTSD1	23.692142
	101141-dfom	CTSD1	23.68033
	101141-dfom	CTSD1	23.68033
**Fig.7M**	siNC	actin	17.271753
	siNC	actin	17.302202
	siNC	actin	17.302202
	siNC-101141	actin	16.574345
	siNC-101141	actin	16.65729
	siNC-101141	actin	16.574345
	siNC-101141-DFOM	actin	17.209785
	siNC-101141-DFOM	actin	17.295303
	siNC-101141-DFOM	actin	17.209785
	siKHSRP	actin	15.481748
	siKHSRP	actin	15.481748
	siKHSRP	actin	14.829265
	siKHSRP-101141	actin	23.277725
	siKHSRP-101141	actin	23.277725
	siKHSRP-101141	actin	23.053734
	siNC	CTSD1	22.721008
	siNC	CTSD1	22.719925
	siNC	CTSD1	22.719925
	siNC-101141	CTSD1	22.645786
	siNC-101141	CTSD1	22.679333
	siNC-101141	CTSD1	22.645786
	siNC-101141-DFOM	CTSD1	22.50381
	siNC-101141-DFOM	CTSD1	22.50381
	siNC-101141-DFOM	CTSD1	22.295296
	siKHSRP	CTSD1	23.171322
	siKHSRP	CTSD1	23.12947
	siKHSRP	CTSD1	23.12947
	siKHSRP-101141	CTSD1	26.378424
	siKHSRP-101141	CTSD1	26.378424
	siKHSRP-101141	CTSD1	26.313717
**Fig.S4K**	MCF-7 con	GAPDH	15.48828537
	MCF-7 con	GAPDH	15.4561839
	MCF-7 con	GAPDH	15.4561839
	MCF-7 pac	GAPDH	15.10095124
	MCF-7 pac	GAPDH	15.07240725
	MCF-7 pac	GAPDH	15.10422395
	MCF-7 101141	GAPDH	14.89141387
	MCF-7 101141	GAPDH	14.89141387
	MCF-7 101141	GAPDH	14.87617263

MCF-7 con	SOX2	24.7527785
MCF-7 con	SOX2	24.7671849
MCF-7 con	SOX2	24.7527785
MCF-7 pac	SOX2	24.94651324
MCF-7 pac	SOX2	24.80496039
MCF-7 pac	SOX2	24.88663823
MCF-7 101141	SOX2	25.21449187
MCF-7 101141	SOX2	25.21449187
MCF-7 101141	SOX2	25.20657034
MCF-7 con	NANOG	28.07038123
MCF-7 con	NANOG	28.07038123
MCF-7 con	NANOG	28.03760168
MCF-7 pac	NANOG	27.97923472
MCF-7 pac	NANOG	28.03551045
MCF-7 pac	NANOG	28.13478313
MCF-7 101141	NANOG	30.2365083
MCF-7 101141	NANOG	30.2365083
MCF-7 101141	NANOG	30.1494485
MCF-7 con	NOTCH1	27.4880923
MCF-7 con	NOTCH1	27.31117427
MCF-7 con	NOTCH1	27.4880923
MCF-7 pac	NOTCH1	27.14424611
MCF-7 pac	NOTCH1	27.14902773
MCF-7 pac	NOTCH1	27.15679659
MCF-7 101141	NOTCH1	26.96946899
MCF-7 101141	NOTCH1	27.00035609
MCF-7 101141	NOTCH1	26.96946899
control	GAPDH	16.47779152
control	GAPDH	16.47351301
control	GAPDH	16.48822257
pac	GAPDH	16.33811029
pac	GAPDH	16.33811029
pac	GAPDH	16.33708494
101141	GAPDH	15.89878432
101141	GAPDH	15.93359871
101141	GAPDH	15.93359871
control	EpCAM	18.06930931
control	EpCAM	18.02433714
control	EpCAM	18.02433714
pac	EpCAM	17.84445615
pac	EpCAM	17.79544838
pac	EpCAM	17.79350863
101141	EpCAM	17.74464324
101141	EpCAM	17.71312753
101141	EpCAM	17.71312753
control	ALDHA1A3	25.4325322
control	ALDHA1A3	25.38837982
control	ALDHA1A3	25.38837982
pac	ALDHA1A3	24.03608723
pac	ALDHA1A3	24.03608723
pac	ALDHA1A3	24.07318758
101141	ALDHA1A3	25.52373846
101141	ALDHA1A3	25.53671265
101141	ALDHA1A3	25.52373846
control	CD24	18.25532371
control	CD24	18.23254934
control	CD24	18.25532371
pac	CD24	18.12727853

	pac	CD24	18.04554722
	pac	CD24	18.12685372
	101141	CD24	18.81526315
	101141	CD24	18.81526315
	101141	CD24	18.82841975
	control	CD44	21.29918929
	control	CD44	21.30548619
	control	CD44	21.30548619
	pac	CD44	21.11880771
	pac	CD44	21.24454888
	pac	CD44	21.24454888
	101141	CD44	21.29613282
	101141	CD44	21.33219528
	101141	CD44	21.35324336
	control	CD36	21.9328474
	control	CD36	21.89527879
	control	CD36	21.89527879
	pac	CD36	22.01375112
	pac	CD36	22.01375112
	pac	CD36	22.03971372
	101141	CD36	23.26938218
	101141	CD36	23.26938218
	101141	CD36	23.31044653
**Fig.S4L**	MB453 con	GAPDH	16.10017382
	MB453 con	GAPDH	16.10067337
	MB453 con	GAPDH	16.10067337
	MB453 pac	GAPDH	16.29027453
	MB453 pac	GAPDH	16.25020074
	MB453 pac	GAPDH	16.28768313
	MB453 101141	GAPDH	16.39702965
	MB453 101141	GAPDH	16.41032343
	MB453 101141	GAPDH	16.39702965
	MB453 con	SOX2	29.03507221
	MB453 con	SOX2	29.06309806
	MB453 con	SOX2	29.0887718
	MB453 pac	SOX2	29.26372588
	MB453 pac	SOX2	29.26028033
	MB453 pac	SOX2	29.26028033
	MB453 101141	SOX2	30.03421403
	MB453 101141	SOX2	30.03421403
	MB453 101141	SOX2	30.09366567
	MB453 con	NANOG	25.1031414
	MB453 con	NANOG	25.06716232
	MB453 con	NANOG	25.1031414
	MB453 pac	NANOG	26.05105376
	MB453 pac	NANOG	26.18548897
	MB453 pac	NANOG	26.13361088
	MB453 101141	NANOG	28.86106261
	MB453 101141	NANOG	28.75755789
	MB453 101141	NANOG	28.68820087
	MB453 con	NOTCH1	26.75160794
	MB453 con	NOTCH1	26.75160794
	MB453 con	NOTCH1	26.70246401
	MB453 pac	NOTCH1	26.40294783
	MB453 pac	NOTCH1	26.40294783
	MB453 pac	NOTCH1	26.25546541
	MB453 101141	NOTCH1	26.87252515

	MB453 101141	NOTCH1	26.93783741
	MB453 101141	NOTCH1	26.87252515
	MB453 con	CD133	29.07845718
	MB453 con	CD133	29.14961969
	MB453 con	CD133	29.21080126
	MB453 pac	CD133	27.36291427
	MB453 pac	CD133	27.48765964
	MB453 pac	CD133	27.48657945
	MB453 101141	CD133	29.51037583
	MB453 101141	CD133	29.62833991
	MB453 101141	CD133	29.62833991
	MB453 con	CD24	19.06413175
	MB453 con	CD24	19.06413175
	MB453 con	CD24	19.06413175
	MB453 pac	CD24	18.19232589
	MB453 pac	CD24	18.19232589
	MB453 pac	CD24	18.20434713
	MB453 101141	CD24	19.76701463
	MB453 101141	CD24	19.72810882
	MB453 101141	CD24	19.72810882
	MB453 CON	GAPDH	16.35233034
	MB453 CON	GAPDH	16.31458474
	MB453 CON	GAPDH	16.29808585
	MB453 PAC	GAPDH	16.85210947
	MB453 PAC	GAPDH	16.85550118
	MB453 PAC	GAPDH	16.85210947
	MB453 101141	GAPDH	16.93056647
	MB453 101141	GAPDH	16.93056647
	MB453 101141	GAPDH	16.93581087
	MB453 CON	EPCAM	18.31776669
	MB453 CON	EPCAM	18.2638384
	MB453 CON	EPCAM	18.31776669
	MB453 PAC	EPCAM	18.12915561
	MB453 PAC	EPCAM	18.23686516
	MB453 PAC	EPCAM	18.12915561
	MB453 101141	EPCAM	18.29023184
	MB453 101141	EPCAM	18.29023184
	MB453 101141	EPCAM	18.30309168
	MB453 CON	ALDHA1A3	31.08427076
	MB453 CON	ALDHA1A3	31.22326329
	MB453 CON	ALDHA1A3	31.08427076
	MB453 PAC	ALDHA1A3	31.1355946
	MB453 PAC	ALDHA1A3	30.91680927
	MB453 PAC	ALDHA1A3	30.91680927
	MB453 101141	ALDHA1A3	32.4478898
	MB453 101141	ALDHA1A3	32.31573214
	MB453 101141	ALDHA1A3	32.31573214
	MB453 CON	CD36	26.07292328
	MB453 CON	CD36	26.07292328
	MB453 CON	CD36	26.06886094
	MB453 PAC	CD36	26.82244439
	MB453 PAC	CD36	26.82244439
	MB453 PAC	CD36	26.77884788
	MB453 101141	CD36	29.06865325
	MB453 101141	CD36	29.06865325
	MB453 101141	CD36	29.14595345
**Fig.S6A**	PLKO.1	GAPDH	14.72211328

	PLKO.1	GAPDH	14.65984196
	PLKO.1	GAPDH	14.85597385
	sh-HDGF-1	GAPDH	15.30219862
	sh-HDGF-1	GAPDH	15.41762986
	sh-HDGF-1	GAPDH	15.28708259
	sh-HDGF-2	GAPDH	15.40685928
	sh-HDGF-2	GAPDH	15.45246623
	sh-HDGF-2	GAPDH	15.24141879
	PLKO.1	HDGF	19.24397596
	PLKO.1	HDGF	19.42387724
	PLKO.1	HDGF	19.27904701
	sh-HDGF-1	HDGF	20.70734073
	sh-HDGF-1	HDGF	20.43116658
	sh-HDGF-1	HDGF	20.53795641
	sh-HDGF-2	HDGF	23.34801012
	sh-HDGF-2	HDGF	23.27109247
	sh-HDGF-2	HDGF	23.11602562
**Fig.S6B**	PLKO.1	GAPDH	23.22926478
	PLKO.1	GAPDH	23.37154448
	PLKO.1	GAPDH	23.1767095
	sh-CYP2S1-1	GAPDH	21.54111852
	sh-CYP2S1-1	GAPDH	21.47915175
	sh-CYP2S1-1	GAPDH	21.8945136
	sh-CYP2S1-2	GAPDH	22.31403348
	sh-CYP2S1-2	GAPDH	22.13621491
	sh-CYP2S1-2	GAPDH	22.12938237
	PLKO.1	CYP2S1	36.50464641
	PLKO.1	CYP2S1	36.56283717
	PLKO.1	CYP2S1	36.61775263
	sh-CYP2S1-1	CYP2S1	36.08185059
	sh-CYP2S1-1	CYP2S1	35.623572
	sh-CYP2S1-1	CYP2S1	36.35906197
	sh-CYP2S1-2	CYP2S1	37.08018002
	sh-CYP2S1-2	CYP2S1	37.09938725
	sh-CYP2S1-2	CYP2S1	36.05809305
